# HER2∆16 directs luminal cell identity and estrogen receptor signaling in HER2+ breast cancer

**DOI:** 10.1038/s41467-026-74435-9

**Published:** 2026-06-15

**Authors:** Hailey Proud, Elizabeth Podleszanski, Sherif S. Attalla, Ellie J. Massey, Tarek Taifour, Alexandra Eric, Dongmei Zuo, Chen Ling, Alain Pacis, Harvey W. Smith, Vasilios Papavasiliou, Virginie Sanguin-Gendreau, William J. Muller

**Affiliations:** 1 Dept. Biochemistry, Faculty of Medicine and Health Sciences, McGill University, Montreal, QC Canada; 2 Goodman Cancer Institute, Faculty of Medicine and Health Sciences, McGill University, Montreal, QC Canada; 3 Dept. Experimental Medicine, Faculty of Medicine and Health Sciences, McGill University, Montreal, QC Canada; 4 Canadian Centre for Computational Genomics, McGill University, Montreal, QC Canada

**Keywords:** Breast cancer, Tumour heterogeneity, Cancer models, Cancer genetics, Cancer therapeutic resistance

## Abstract

Co-expression of the estrogen receptor (ER) and human epidermal growth factor receptor 2 (HER2) contributes to breast cancer heterogeneity and therapeutic resistance. However, the molecular mechanisms promoting ER positivity within HER2+ cancers remains largely unknown. Here we show, across HER2+ transgenic mouse models the oncogenic HER2 splice variant lacking exon 16 (HER2∆16) promotes the development of aggressive luminal tumors by facilitating an ER-mediated transcriptional program which is sensitive to endocrine therapies. HER2∆16 is detected across human HER2+ breast tumors and cell lines with higher levels correlating with increased expression of ER and downstream transcriptional targets. Notably, in human cell lines HER2∆16 expression is elevated upon acquired resistance to HER2-targeted therapy and can sensitize cells to the ER-antagonist tamoxifen. Overall, these findings offer valuable insights into the role of HER2∆16 in promoting luminal cell identity and estrogen receptor positivity in breast cancer, providing a useful platform to model HER2+/ER+ disease.

## Abstract (web summary)

Breast cancers with HER2 overexpression can vary in whether they also express the estrogen receptor, influencing tumor behavior and treatment response. Here, the authors show that in transgenic mouse models, the HER2Δ16 splice variant promotes the development of aggressive luminal tumors by facilitating luminal cell differentiation and driving estrogen receptor signaling that is sensitive to endocrine therapy.

## Introduction

The identification of human epidermal growth factor receptor 2 (HER2/ERBB2) as a prognostic and predictive factor in breast cancer has profoundly impacted clinical outcomes in HER2-positive patients. HER2 is the preferred heterodimerization partner of the epidermal growth factor receptor (EGFR) family of receptor tyrosine kinases (RTK), with HER2-containing heterodimers typically achieving the most robust activation of the PI3K/AKT/mTOR and RAS/MAPK signaling cascades^1^. Approximately 15–20% of breast cancer cases overexpress HER2, predominantly through gene amplification, which leads to aggressive disease and poor prognosis^2,3^. Despite the introduction of the monoclonal anti-HER2 antibodies trastuzumab/pertuzumab and subsequent development of small molecule HER2 RTK inhibitors and antibody-drug conjugates, predicting responses and risk of relapse remains a major clinical challenge^4^. This is partially due to the extensive network of tumor extrinsic (immune infiltration, vascularization, stromal cell activation) and intrinsic events (genomic mutations, sustained signaling pathways, luminal/basal differentiation) which dictate therapeutic response and contribute to resistance^5–9^. Indeed, these cellular events lead to substantial biological heterogeneity across HER2-positive tumors, with varying molecular mechanisms driving tumor aggressiveness, something which is not currently considered when planning treatment strategies^10,11^.

Recent studies have further stratified HER2-positive breast cancer, identifying five molecular subtypes associated with disease recurrence based on gene expression profiling of tumor samples from patients receiving adjuvant trastuzumab: immune-enriched, proliferative/metabolic-enriched, mesenchymal/stroma-enriched, luminal, and ERBB2-dependent^12^. Of note, the luminal breast cancer subtype displayed increased estrogen receptor (ER) expression, encoded by the *ESR1* gene, and a lower probability of achieving complete response to trastuzumab despite being associated with better prognosis^12^. HER2+/ER+ tumors account for 10% of all breast cancer cases, and co-expression has been linked to worse response to either HER2-targeted or endocrine therapy^13–16^. These findings highlight the importance of understanding how crosstalk between ER and HER2 signaling pathways alters tumor behavior. A proportion of HER2+ /ER- tumors upregulate ER signaling as an adaptive survival mechanism post-HER2 therapy, suggesting that phenotypic plasticity can increase the subset of HER2+ tumors that can be stratified within the HER2+ /ER+ luminal subtype^17^. Determining the mechanisms directing this switch to ER-positivity could provide insights with the potential to improve predictions of clinical response and overcome therapeutic resistance by using endocrine therapies within this patient subset.

A splice variant of HER2 lacking exon 16 (HER2∆16) makes up 4-10% of HER2 transcript within breast tumors and is a driver of tumor aggressiveness and biological heterogeneity^18^. HER2∆16 promotes constitutive HER2-signaling primarily through the formation of disulfide-bonded homodimers and has been implicated in several solid malignancies including breast, lung and colorectal cancers^18–21^. Mammary epithelial-specific expression of HER2∆16 in transgenic mice results in the rapid development of metastatic multifocal tumors and confers resistance to both trastuzumab and trastuzumab-emtansine in vitro^18,22^. In breast cancer patients, HER2∆16 expression is strongly correlated with dissemination to the lymph nodes, a widely used predictor of poor prognosis, consistent with its involvement in metastatic progression^18^. Previous studies have shown that mammary tumors expressing HER2∆16 display a distinct proteomic profile which is associated with the generation of an immunosuppressive tumor microenvironment^23^. However, the mammary epithelial cell-autonomous effects of HER2∆16 and its impact on cellular differentiation within the HER2+ subtype remain unexplored. Furthermore, the lack of GEMMs that faithfully model human HER2+ /ER+ breast cancer has been a major limiting factor in the advancement of research within this area.

Here, we show that GEMMs expressing the HER2∆16 isoform display an aggressive luminal breast cancer phenotype, promoting an ER-driven transcriptional program and conferring increased sensitivity to endocrine therapy. By establishing a GEMM expressing endogenous levels of murine ErbB2∆16, we further demonstrate that the alternatively spliced isoform directs tumors towards a luminal ER-positive cellular identity which can be targeted effectively using endocrine therapies. Tumor-derived allografts from this model provide a preclinical platform for evaluating the use of single-agent endocrine or combination therapies within an immune-competent in vivo system, something currently lacking within the field^24^. In addition to its impact on ER signaling, HER2∆16 expression shifts towards a more luminal-differentiated cell state which correlates with increased expression of luminal cytokeratins (CK8) and differentiation markers such as EpCAM. Consistent with this luminal phenotype, we observe a corresponding increase in transcription factors driving luminal differentiation. In human breast cancer cells and tissue microarray data, elevated expression of HER2∆16 corresponds with increased ER levels and transcriptional activity as well as resistance to HER2 inhibition, with resistant cells exhibiting sensitization to endocrine therapy. Taken together, these data suggest a role for HER2∆16 in promoting estrogen receptor signaling and tamoxifen sensitivity by driving luminal cell fate while fulfilling an unmet need for a preclinical GEMM to model HER2+ /ER+ disease.

## Results

### Expression of HER2∆16 induces aggressive mammary tumors that exhibit an epithelial-differentiated phenotype

**Fig. 1:**
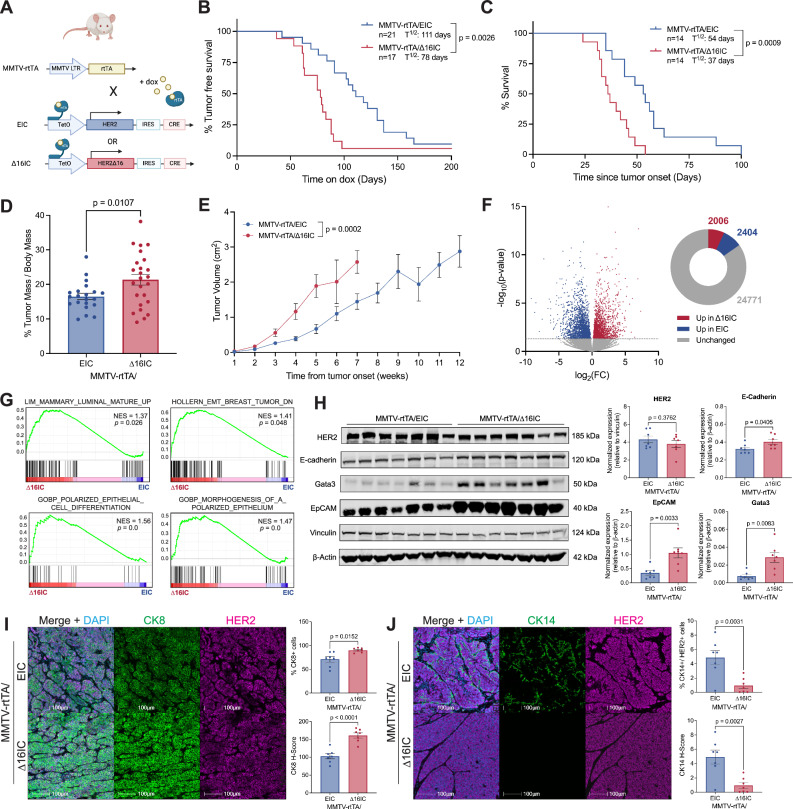
Mammary epithelial expression of HER2∆16 associates with an aggressive luminally differentiated breast cancer phenotype. **A** Schematic of MMTV-rtTA/EIC and ∆16IC genetically engineered mouse models. Created in BioRender. Muller, W. (2026) https://BioRender.com/cia744j. **B** Kaplan–Meier curve of mammary tumor onset in EIC (*n* = 21) and ∆16IC (*n* = 17) mice. Analysis by log-rank (Mantel-Cox) test. **C** Survival of tumor-bearing mice from day of tumor onset until humane endpoint in EIC (*n* = 14) and ∆16IC (*n* = 14) cohorts. Analysis by log-rank (Mantel–Cox) test. **D** Tumor burden as a percentage of total body mass occupied by the tumor(s) in EIC (*n* = 21) and ∆16IC (*n* = 25) mice. **E** Tumor outgrowth as determined by caliper measurement of tumor volume in EIC (*n* = 19) and ∆16IC (*n* = 15) cohorts at weekly timepoints. Statistical analysis performed by Compare Groups of Growth Curves (CGGC) permutation analysis. **F** Volcano plot of RNA-sequencing results from EIC and ∆ 16IC tumors (*n* = 5 per genotype) highlighting differentially expressed genes (adjusted *p* < 0.05). **G** GSEA for signatures of mammary luminal cells, epithelial cell differentiation and epithelial to mesenchymal transition in ∆16IC versus EIC tumors. **H** Immunoblots for HER2, Epithelial-cadherin, Gata3, and EpCAM from bulk tumor lysates of EIC and ∆16IC mice (*n* = 7 tumors per genotype) at tumor endpoint, normalized to vinculin or β-actin. **I** Immunofluorescent staining for HER2 and cytokeratin 8 (CK8) on 7 tumors per genotype, counterstained with DAPI. Percentage CK8+ cells and average fluorescent intensity (H-score) was quantified via HALO. **J** Immunofluorescent staining for HER2 and cytokeratin 14 (CK14) on 6 tumors per genotype, counterstained with DAPI. Percentage CK14 +/HER2+ cells and average fluorescent intensity (H-score) was quantified via HALO. All error bars are expressed as mean values  ±  SEM. All statistical analysis by unpaired, two-tailed Student’s *t*-test with Welch’s correction unless otherwise indicated. Source data are provided as a Source Data file.

Using two distinct GEMMs with doxycycline-inducible, mammary epithelial-specific expression of full-length human HER2 (EIC) or the HER2∆16 isoform (∆16IC) (Fig. 1A), we confirmed previous findings that expression of either isoform resulted in the development of multifocal mammary tumors (Supplementary Fig. 1A–C), with the average tumor latency significantly decreased in the ∆16IC strain relative to EIC (78 vs. 111 days, Fig. 1B)^23^. ∆16IC tumors also reached humane endpoint significantly faster than their EIC counterparts (37 vs. 54 days) while harboring a greater overall tumor burden (Fig. 1C–E)^23,25^. Both strains developed spontaneous metastases to the lung, exhibiting high levels of the epithelial cell adhesion marker, EpCAM, with ∆16IC lesions having a higher metastatic incidence (Supplementary Fig. 1D–F). Overall, these findings show that HER2∆16 expression promotes an aggressive breast tumor phenotype.

To investigate potential transcriptional changes driven by HER2∆16 which may impact HER2+ tumorigenesis, we performed mRNA-seq analyses on endpoint EIC and ∆16IC tumors. Each HER2 variant was associated with a distinct transcriptomic profile, consistent with the observed pathological differences (Fig. 1F). Interestingly, gene sets and pathways enriched in ∆16IC compared to EIC tumors were associated with mammary epithelial cell differentiation, polarization and luminal identity (Fig. 1G, Supplementary Fig. 2A), as well as stabilization and expansion of adherens junctions, a key mediator of epithelial signaling (Supplementary Fig. 2B). Validating these findings, we detected elevated protein expression of the epithelial/luminal cell markers GATA3, EpCAM and E-cadherin in ∆16IC tumors (Fig. 1H). Notably, there was no difference in HER2 protein expression, confirming that phenotypes could not be attributed to varying levels of HER2. The ∆16IC primary tumors also contained a higher percentage of cells expressing luminal epithelial cytokeratin 8 (CK8) with elevated average immunofluorescent staining intensity, consistent with increased CK8 expression (Fig. 1I). In contrast, EIC tumors retained expression of the basal cytokeratin 14 (CK14) within the HER2+ tumor cell population, which was completely absent in ∆16IC lesions (Fig. 1J). A loss of CK14-positive cells has previously been observed in the stromal cell compartment of MMTV-driven ∆16IC tumors, representing dissolution of the myoepithelial layer and acceleration of tumor progression^23^. Taken together, these findings argue that the rapid initiation and growth of HER2∆16-driven mammary tumors are associated with a luminal epithelial cell state.

### HER2∆16 facilitates estrogen-receptor mediated signaling

**Fig. 2:**
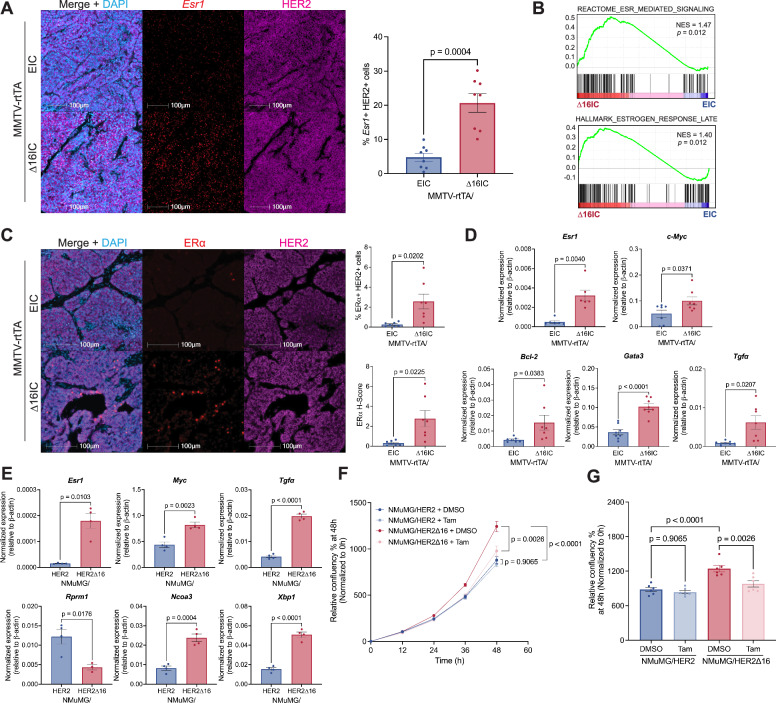
HER2∆16 promotes expression of Esr1 and transcription of downstream targets. **A** RNA ISH for *Esr1* transcript levels, followed by immunofluorescent HER2 protein staining in EIC and ∆16IC end-point tumors (*n* = 8 tumors per genotype). Percentage *Esr1* +/HER2+ cells was quantified via HALO. **B** GSEA for signatures of estrogen receptor signaling in ∆16IC versus EIC tumors. **C** Immunofluorescent staining for HER2 and Estrogen Receptor α (ERα) on 7 tumors per genotype, counterstained with DAPI. Percentage ERα + /HER2+ cells and average fluorescent intensity (H-score) was quantified via HALO. **D** Quantitative real-time polymerase chain reaction (qRT-PCR) for ER-target genes *Esr1* (*n* = 6), *c-Myc* (*n* = 7), *Bcl-2* (*n* = 7), *Gata3* (*n* = 8) and *Tgfα* (*n* = 8) in EIC and ∆16IC end-point tumors normalized to *β-actin*. **E** qRT-PCR for ER-target gene expression normalized to *β-actin* in NMuMG cells expressing either HER2 or HER2∆16 (*n* = 4 per genotype). **F** Proliferative capacity of NMuMG HER2 and HER2∆16 cells treated with either 2 μM tamoxifen (Tam) or dimethylsulfoxide (DMSO) assayed by Incucyte over 48 h (data normalized to confluency at t = 0). Each cell line was tested in sextuplicate. **G** Relative confluency of control or tamoxifen (Tam) treated NMuMG cells at 48 h, relative to t = 0 (*n* = 6 per treatment group), statistical significance determined by one-way ANOVA with Tukey’s post-hoc test. All error bars are expressed as mean values  ±  SEM. All statistical analysis by unpaired, two-tailed Student’s *t*-test with Welch’s correction unless otherwise indicated. Source data are provided as a Source Data file.

Within the HER2 molecular subtype, a luminal tumor phenotype has been linked to higher *ESR1* expression^12^. We found a significant increase in tumor cells expressing *Esr1* mRNA in ∆16IC compared to EIC tumors (Fig. 2A), correlating with enrichment of several transcriptional pathways linked to estrogen response and ER-mediated signaling in the ∆16IC model (Fig. 2B, Supplementary Fig. 2C), including significant pathway enrichment for both early and late estrogen responses (Supplementary Fig. 2B). Although both EIC and ∆16IC tumors display *Esr1* transcript within the primary lesions, the EIC model exhibits minimal ERα protein expression with <1% ER+ cells within the primary tumor (Fig. 2C). In contrast, ∆16IC tumors retain robust ER expression, up to 6% of the total HER2+ cell population, demonstrating that while EIC tumors remain stratified within the HER2+ molecular subtype the ∆16IC model more closely resembles a HER2+ /ER+ tumor type (Fig. 2C).

Validating the activation of ER-dependent signaling in ∆16IC tumors, we detected elevated transcript levels of *Esr1* itself and of several canonical ER target genes that contain estrogen response elements within their promoter regions or directly interact with ER, including *Myc, Tgfα, Bcl-2* and the luminal transcription factor *Gata3* (Fig. 2D). Further supporting the activation of ER-mediated transcriptional and signaling programs in ∆16IC tumors, we used a bioinformatic technique that infers biological activities from transcriptomic datasets (DecoupleR) to show activation of several ER-target genes which are upregulated within ∆16IC tumors (Supplementary Fig. 2D)^26^.

To determine whether expression of HER2∆16 is sufficient to drive *Esr1-*dependent gene expression in vitro, we used a murine mammary epithelial-like cell line (NMuMG) expressing either full-length human HER2 or HER2∆16 at comparable levels^22^. After confirming exclusive expression of the HER2 variants in these cells (Supplementary Fig. 3A–B), we detected significantly higher levels of *Esr1* and several canonical downstream targets, as well as a reduction in *Rprm1*, a gene negatively regulated by estrogen, in NMuMG/HER2∆16 cells (Fig. 2E)^27^. Interestingly, there is only partial overlap in the ER-targets enriched by both the GEMM strain and the immortalized cell line, highlighting the heterogeneity between the model systems (Fig. 2D, Fig. 2E). Of note, *Ncoa3*, a potent coactivator of ER was upregulated in NMuMG/HER2∆16 cells, implying that HER2∆16 may also facilitate expression of ER interactors in addition to *Esr1* itself (Fig. 2E)^28^.

Because expression of luminal cell markers correlates with better response to endocrine therapy in ER-positive breast cancer, we treated HER2 isoform-expressing cell lines with the selective estrogen receptor modulator (SERM), tamoxifen^29^. Tamoxifen treatment significantly impaired proliferation in HER2∆16-expressing cells, but not their full length HER2-expressing counterparts (Fig. 2F–G). These results underscore a reliance on ER-mediated signaling to support the growth of HER2∆16-expressing cells, revealing their underlying susceptibility to endocrine therapy. Overall, our findings establish that HER2∆16 expression is sufficient to drive a tumor cell-intrinsic *Esr1*-dependent transcriptional program^23^.

### Endogenous ErbB2∆16 expression is sufficient to drive *ErbB2* amplification and ER-positive tumorigenesis

**Fig. 3:**
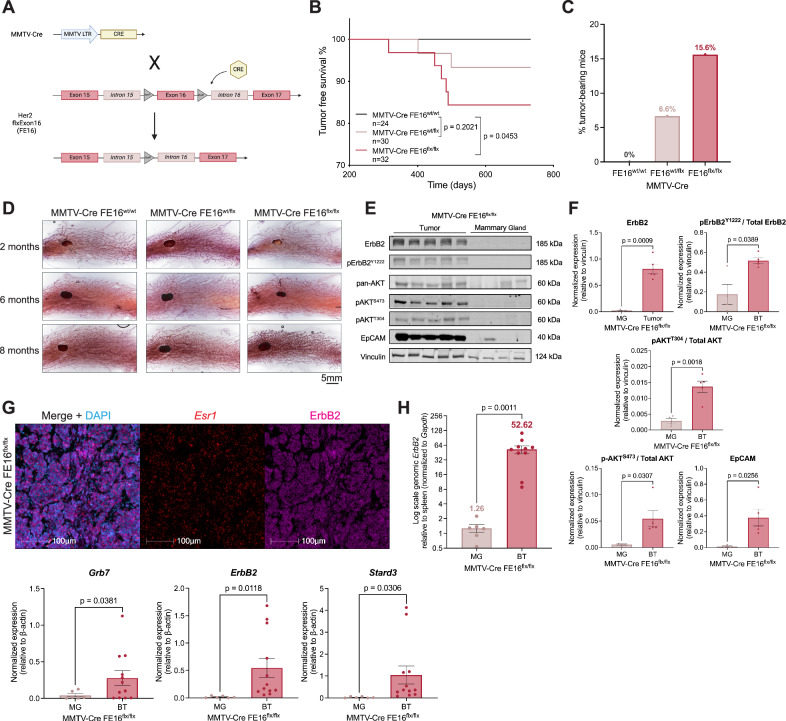
Endogenous expression of murine ErbB2∆16 is capable of driving HER2+/ER+ mammary tumorigenesis and promotes ErbB2 amplification. **A** Schematic of the MMTV-Cre FE16 genetically engineered mouse model, created in BioRender. Muller, W. (2026) https://BioRender.com/mvdul9u. **B** Kaplan–Meier curve of mammary tumor onset in MMTV-Cre FE16^wt/wt^ (*n* = 24), MMTV-Cre FE16^wt/flx^ (*n* = 30) and MMTV-Cre FE16^flx/flx^ (*n* = 32) mice. Analysis by log-rank (Mantel–Cox) test. **C** Incidence of tumor onset in transgenic mice, shown as percentage tumor penetrance within total population. **D** Representative hematoxylin-stained whole mounts of mammary glands from MMTV-Cre FE16^wt/wt^, MMTV-Cre FE16^wt/flx^, and MMTV-Cre FE16^flx/flx^ mice at 2-, 6- and 8-months of age. Images are representative of 8 independent tumors from each genotype. Scale bar represents 5 mm. **E**–**F** Immunoblots and relative quantification for ErbB2, pErbB2 (Tyr1222), pan-AKT, pAKT (Ser473), pAKT (Thr304) and EpCAM from bulk tumor lysate (*n* = 5) or age-matched mammary glands (*n* = 4) in MMTV-Cre FE16^flx/flx^ mice, normalized to vinculin. **G** Representative RNA ISH for *Esr1* transcript levels, followed by immunofluorescent ErbB2 protein staining in endpoint MMTV-Cre FE16^flx/flx^ tumors. **H** Genomic qPCR quantifying *ErbB2* copy number from age-matched mammary glands and endpoint tumors in MMTV-Cre FE16^flx/flx^ mice. Data was normalized to *Gapdh*, and copy number was normalized relative to spleen. **I** qRT-PCR for HER2/ErbB2 amplicon genes *ErbB2*, *Grb7*, and *Stard3* normalized to *β-actin* in MMTV-Cre FE16^flx/flx^ endpoint tumors (*n* = 12) compared to mammary glands from age-matched mice (*n* = 6). All error bars are expressed as mean values  ±  SEM. All statistical analysis by unpaired, two-tailed Student’s *t*-test with Welch’s correction unless otherwise indicated. Source data are provided as a Source Data file.

In murine models, mammary epithelial-specific expression of the *ErbB2* proto-oncogene often coincides with additional mutations within the juxtamembrane region, suggesting these mutations confer a selective advantage to the *ErbB2* transgene^20,30,31^. To assess whether expressing endogenous levels of the highly active ErbB2∆16 isoform is sufficient to promote HER2+/ER+ oncogenesis, we used genome editing to develop a GEMM with conditional expression of ErbB2∆16 (floxed exon 16 model - FE16) upon MMTV-Cre mediated recombination of LoxP sites inserted into the introns surrounding exon 16 of the murine *ErbB2* gene (Fig. 3A). This approach enables constitutive expression of ErbB2∆16 exclusively in the mammary epithelium from the endogenous locus. A subset of FE16 mice homozygous for the LOXP1 flanked allele (FE16^flx/flx^) developed mammary tumors after a long latency period (Fig. 3B). Mammary tumorigenesis was less penetrant in heterozygotes but occurred with comparable latency (Fig. 3C). Mammary glands from FE16^flx/flx^ mice exhibited hyperplasia beginning at 8 months of age, which was not detected in the heterozygous group, however this may be reflective of the small proportion of FE16^wt/flx^ mice which go on to develop mammary tumors (Fig. 3D). Consistent with findings from other HER2∆16 models, FE16^flx/flx^ primary tumors displayed elevated phosphorylation of both ErbB2 and AKT relative to untransformed FE16^flx/flx^ mammary glands, denoting increased ErbB2 signaling (Fig. 3E–F)^18,23,32^. These tumors also expressed high levels of the epithelial marker proteins EpCAM and CK8, as well as *Esr1* and the ER transcriptional targets Ccnd1 and Myc, indicating epithelial cell features akin to luminal ER+ tumors (Fig. 3E–G, Supplementary Fig. 4A–B). Transcript levels of *Esr1* and of estrogen-responsive genes in primary FE16^flx/flx^ tumors were comparable to those observed in MMTV-driven ∆16IC lesions (Supplementary Fig. 4C). Within the small proportion of arising FE16^wt/flx^ tumors, we observe similar elevation of ER expression as well as the epithelial markers CK8 and EpCAM (Supplementary Fig. 5A–B). Furthermore, we discerned no differences in downstream ER-transcriptional activity or the relative expression of *ErbB2∆16* between FE16^wt/flx^ and FE16^flx/flx^ endpoint tumors (Supplementary Fig. 5C). Overall, this suggests that expression of ErbB2∆16 is preferentially selected and sufficient to drive ER+ tumorigenesis within this model, although co-expression of both isoforms decreased the frequency of tumor formation relative to homozygous ErbB2∆16 expression.

Strikingly, we found that the arising FE16^flx/flx^ tumors display a 50-fold increase in genomic *ErbB2* copy number compared to untransformed mammary glands from age-matched FE16^flx/flx^ mice (Fig. 3H). Accordingly, we observed significantly elevated mRNA levels of *ErbB2* and genes found within the clinically observed ErbB2 amplicon, *Stard3* and *Grb7*, in FE16^flx/flx^ tumors (Fig. 3I). Thus, like previous murine ErbB2 knock-in models, genetic amplification of HER2Δ16 appears to be an obligate genetic event involved in both murine and human mammary tumor induction^31^. In this regard, both Stard3 and Grb7 have been shown to sustain the growth of HER2 breast cancer cells, highlighting the importance of genomic amplification events within the HER2/ErbB2 locus in supporting tumor progression^33^. Amplification of the *ErbB2* locus was not detected in either the EIC or ∆16IC GEMMs (Supplementary Fig. 7B), arguing that expression of ErbB2Δ16 under the control of the endogenous promoter may require gene amplification to achieve sufficient ErbB2Δ16 expression to drive tumorigenesis. FE16^flx/flx^ tumors exhibited activation of signaling pathways associated with ErbB2/HER2∆16 expression, including elevated levels of phosphorylated AMPK, Stat3 and the downstream Stat3-target, Chi3l1, the expression of which is consistent with the immune-suppressive signaling promoted by HER2∆16 (Supplementary Fig. 4A)^23^. Interestingly, FE16^wt/flx^ tumors share similar amplification of the *ErbB2* gene, further suggesting this is a driving event supporting tumor initiation within this model (Supplementary Fig. 5D). This data, coupled with the degree of ER activity and expression of epithelial cell markers shared by both models, suggests that in instances of co-expression of both HER2 isoforms, HER2∆16 is determinant in phenotypic outcome. Collectively, these findings establish the FE16^flx/flx^ GEMM as a model of ER-positive HER2 amplified breast cancer, which can be leveraged to study bi-directional ER-HER2 crosstalk in vivo.

### FE16^flx/flx^ tumors are responsive to tamoxifen, eliciting an anti-tumor immune response

A persistent issue with MMTV-driven GEMMs is that the activity of the MMTV promoter is modulated by steroid hormones such as progesterone and estrogen. Thus, using these GEMMs to evaluate the efficacy of endocrine therapies is problematic as it is impossible to discriminate between the direct impact on tumor cells and the indirect effects on MMTV-LTR-driven transcription^31,34^. However, given that expression of ErbB2Δ16 is driven by the murine *Her2*/*ErbB2* endogenous promoter in the FE16^flx/flx^ GEMM, we reasoned that this model could be leveraged as an alternative immune competent GEMM to evaluate the efficacy of endocrine based therapies.

**Fig. 4:**
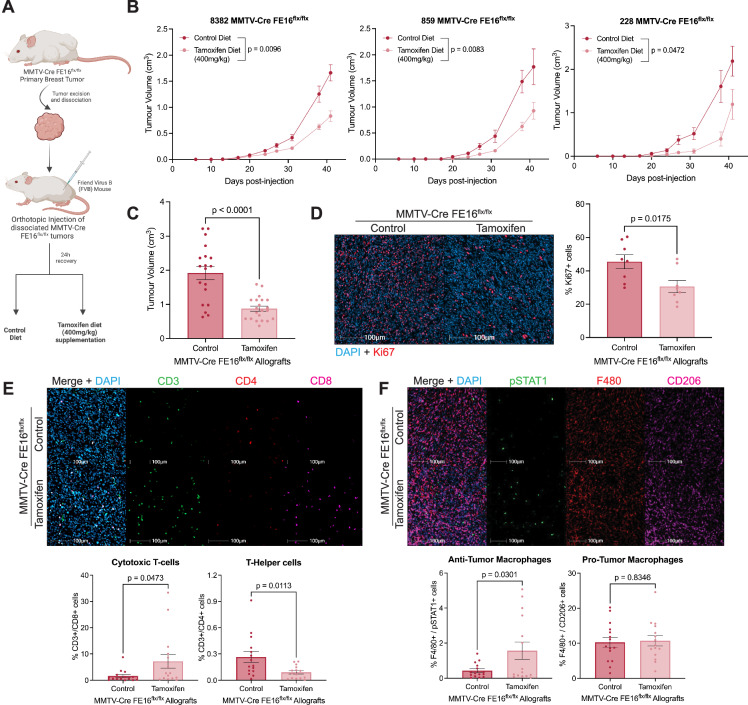
MMTV-Cre FE16^flx/flx^ tumors are sensitive to tamoxifen in vivo and display an anti-tumor immune response. **A** Schematic showing the experimental design of the tamoxifen diet orthotopic xenotransplant study in FVB mice, created in BioRender. Muller, W. (2026) https://BioRender.com/dfo3z85. **B** Tumor outgrowth in orthotopic MMTV-Cre FE16^flx/flx^ tumor xenografts as measured by twice weekly caliper measurement and calculation of tumor volume starting at first palpable mass. Mice were treated with either standard rodent or tamoxifen (400 mg/kg) diet (3 biological replicates, *n* = 5 per treatment group). Statistical analysis performed by Compare Groups of Growth Curves (CGGC) permutation analysis comparing all combinations of treatment groups. **C** Tumor burden as a percentage of total body mass occupied by the tumor(s) at endpoint in control (*n* = 19) and tamoxifen treated (*n* = 21) mice across all cohorts (*p* < 0.0001). **D** Immunofluorescent staining for Ki67 (*n* = 8 per treatment group), counterstained with DAPI. Percentage Ki67+ cells were quantified via HALO. **E** Immunofluorescent staining for CD3, CD4 and CD8 (*n* = 15 per treatment group), counterstained with DAPI. Percentage CD3+/CD4+ and CD3+/CD8+ cells were quantified via HALO. **F** Immunofluorescent staining for pSTAT1, F4/80, and CD206 (*n* = 15 per treatment group), counterstained with DAPI. Percentage pSTAT1/F4/80+ and CD206/F4/80+ cells were quantified via HALO. All error bars are expressed as mean values  ±  SEM. All statistical analysis by unpaired, two-tailed Student’s *t*-test with Welch’s correction unless otherwise indicated. Source data are provided as a Source Data file.

To test whether expressing ErbB2Δ16 from the endogenous promoter can overcome these limitations, we used an orthotopic, immunocompetent allograft model to study tumor development and endocrine therapy response (Fig. 4A). Allografts accurately recapitulated the primary tumors, with the resulting lesions expressing similar levels of both ErbB2 and the luminal epithelial marker CK8, while retaining ER expression (Supplementary Fig. 4B). In three independent tumor lines, tamoxifen significantly delayed tumor growth (Fig. 4B–C), associated with reduced proliferation (Ki67 expression) compared to controls (Fig. 4D). Both ErbB2 and ER levels remained unchanged between treatment groups, signifying that this phenotype is not due to variation in ErbB2 or ER expression (Supplementary Fig. 4D-E).

Although tamoxifen primarily works through reducing ER-mediated cell proliferation, it has also been shown to reprogram the immune landscape of tumors^35^. To investigate this possibility, we assessed the composition and activation status of the tumor immune microenvironment by using multiplexed immunostaining techniques to detect established markers of key immune cell populations. Tamoxifen treated tumors had increased infiltration of both CD8+ cytotoxic T cells and anti-tumor (pSTAT1+ F4/80+ ) macrophages (Fig. 4E–F). Within the CD8+ cytotoxic T cell population we observe an increase in cytotoxic activation as seen by increased levels of granzyme B (Supplementary Fig. 6A). Conversely, we see a decrease in the T-cell exhaustion marker PD1 within the CD3+ T-cell population (Supplementary Fig. 6B). Taken together, these data show that not only is T-cell recruitment increased in tamoxifen treated tumors, but they are polarized towards a robust anti-tumor immune response.

Despite the strong increase in T-cell activity, no differences in pro-tumorigenic macrophages (CD206+ F4/80+ ) or neutrophil populations were observed, arguing that tamoxifen was not sufficient to modulate these immune populations (Fig. 4F, Supplementary Fig. 6C). Unexpectedly, there was a notable decrease in CD4+ T helper cells, which may reflect differences in the effect of tamoxifen on the polarization of T-cell populations (Fig. 4E). Reduced CD4+ T-cell populations, despite the induction of an anti-tumor immune response, have also been reported in clinical samples from tamoxifen treated patients^36^. Collectively, these data establish the FE16^flx/flx^ model as a valuable platform to assess the use of endocrine therapies to target ER-positive, ErbB2+ breast cancer within an immunocompetent model system.

### Endogenous expression of HER2/ErbB2∆16 is sufficient to drive *Esr1*-dependent luminal differentiation in tumors expressing high levels of HER2

**Fig. 5:**
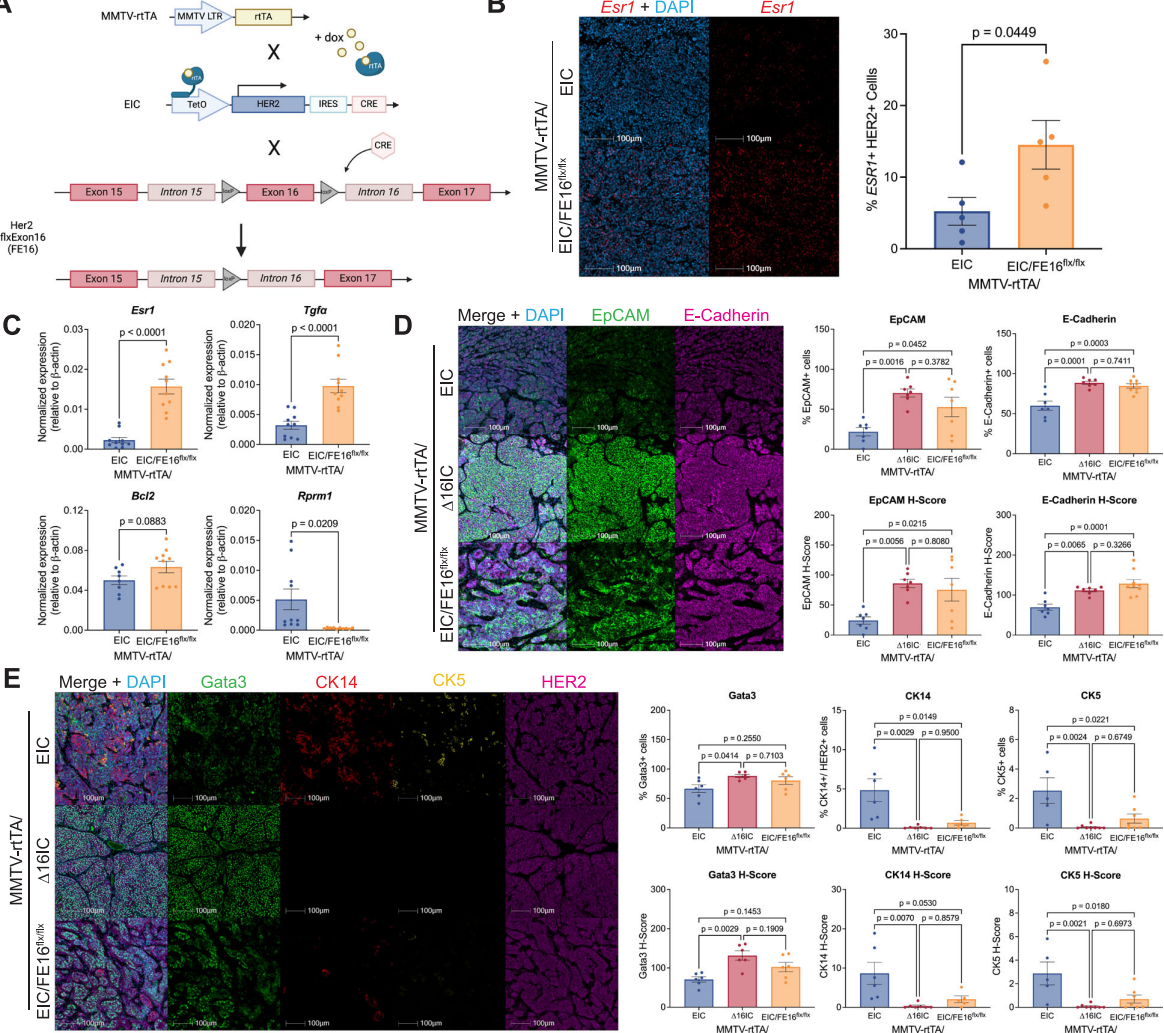
Partial expression of the ErbB2∆16 splice isoform in HER2-driven tumors is sufficient to promote ER expression and drive luminal cell identity. **A** Schematic of MMTV-rtTA/EIC FE16^flx/flx^ genetically engineered mouse models, created in BioRender. Muller, W. (2026) https://BioRender.com/3023xkm. **B** RNA ISH for *Esr1* transcript levels in EIC and EIC FE16^flx/flx^ end-point tumors (*n* = 5 tumors per genotype). Percentage *Esr1+* /HER2+ cells were quantified via HALO with statistical analysis by unpaired, two-tailed Student’s *t*-test with Welch’s correction. **C** Quantitative real-time polymerase chain reaction (qRT-PCR) for ER-target gene expression in EIC and EIC FE16^flx/flx^ end-point tumors normalized to *β-actin* (*n* = 5 tumors per genotype in duplicate). Statistical analysis by unpaired, two-tailed Student’s *t*-test with Welch’s correction. **D** Immunofluorescent staining for epithelial markers EpCAM and E-cadherin (*n* = 7 per genotype), counterstained with DAPI. Percentage EpCAM+ or E-cadherin+ cells and average fluorescent intensity (H-score) were quantified via HALO. Statistical significance determined by one-way ANOVA with Tukey’s post-hoc test. **E** Immunofluorescent staining for epithelial/basal markers Gata3, CK14, CK5 and HER2 (*n* = 6 tumors per genotype), counterstained with DAPI. Percentage positive cells and average fluorescent intensity (H-score) were quantified for each marker via HALO. Statistical significance determined by one-way ANOVA with Tukey’s post-hoc test. All error bars are expressed as mean values  ±  SEM. Source data are provided as a Source Data file.

In HER2∆16-positive breast cancer cases, HER2∆16 makes up 4-10% of total HER2 transcript^18,37,38^. Given that the progression of human HER2+ cancers is associated with expression of both full length and alternatively spliced forms of HER2, we characterized the phenotypes of mammary tumors co-expressing both isoforms. Due to the low penetrance of MMTV-Cre FE16^wt/flx^ tumors, we developed an alternate approach by crossing the MMTV-rtTA/EIC and FE16^flx/flx^ GEMMs, creating a model where doxycycline induction induces mammary epithelial-specific expression of full-length HER2 and Cre-mediated excision of exon 16 within the endogenous murine *ErbB2* gene, leading to co-expression of full-length HER2 and ErbB2∆16, with the latter under the control of its endogenous promoter (Fig. 5A). EIC mice hetero- or homozygous for the FE16 allele developed multifocal mammary tumors, but with a delayed onset relative to wild-type EIC mice (Supplementary Fig. 7A). Expression of full-length HER2 abrogated the requirement for amplification of the endogenous *ErbB2* locus, with all tumors displaying equivalent *ErbB2* copy number, in contrast to the amplification observed in the original FE16^flx/flx^ GEMM (Supplementary Fig. 7B). EIC/FE16^flx/flx^ tumors exhibited elevated levels of *Esr1* transcript compared to wild-type EIC tumors (Fig. 5B–C), with partial recapitulation of the ER-driven transcriptional program observed in the ∆16IC model as well as a similar metastatic incidence and burden (Fig. 5C, Supplementary Fig. 7C–D). Thus, co-expression of full-length and alternatively spliced ErbB2 in this model promotes ER expression with the induction of an ER-dependent transcriptional program that is less pronounced than in tumors expressing ErbB2∆16/HER2∆16 alone. Mammary epithelial expression of the endogenous *ErbB2* alternatively spliced isoform was sufficient to promote epithelial cell differentiation within EIC/FE16^flx/flx^ tumors as endpoint lesions display elevated expression of the epithelial markers EpCAM, E-cadherin and Cytokeratin 8 (Fig. 5D, Supplementary Fig. 7E). Like the MMTV-driven Δ16IC model, these tumors retain minimal expression of basal cytokeratins 5 and 14 (CK5/CK14), indicating a shift towards a luminal cell state (Fig. 5E). Collectively, these results indicate that HER2∆16 is a potent activator of a luminal transcriptional program in ErbB2-positive breast tumors, with coordinated expression of *Esr1* and epithelial cell markers.

### ERBB2∆16 transcript levels correlate with *ESR1* pathway activation and tamoxifen sensitivity in human breast cancer

**Fig. 6:**
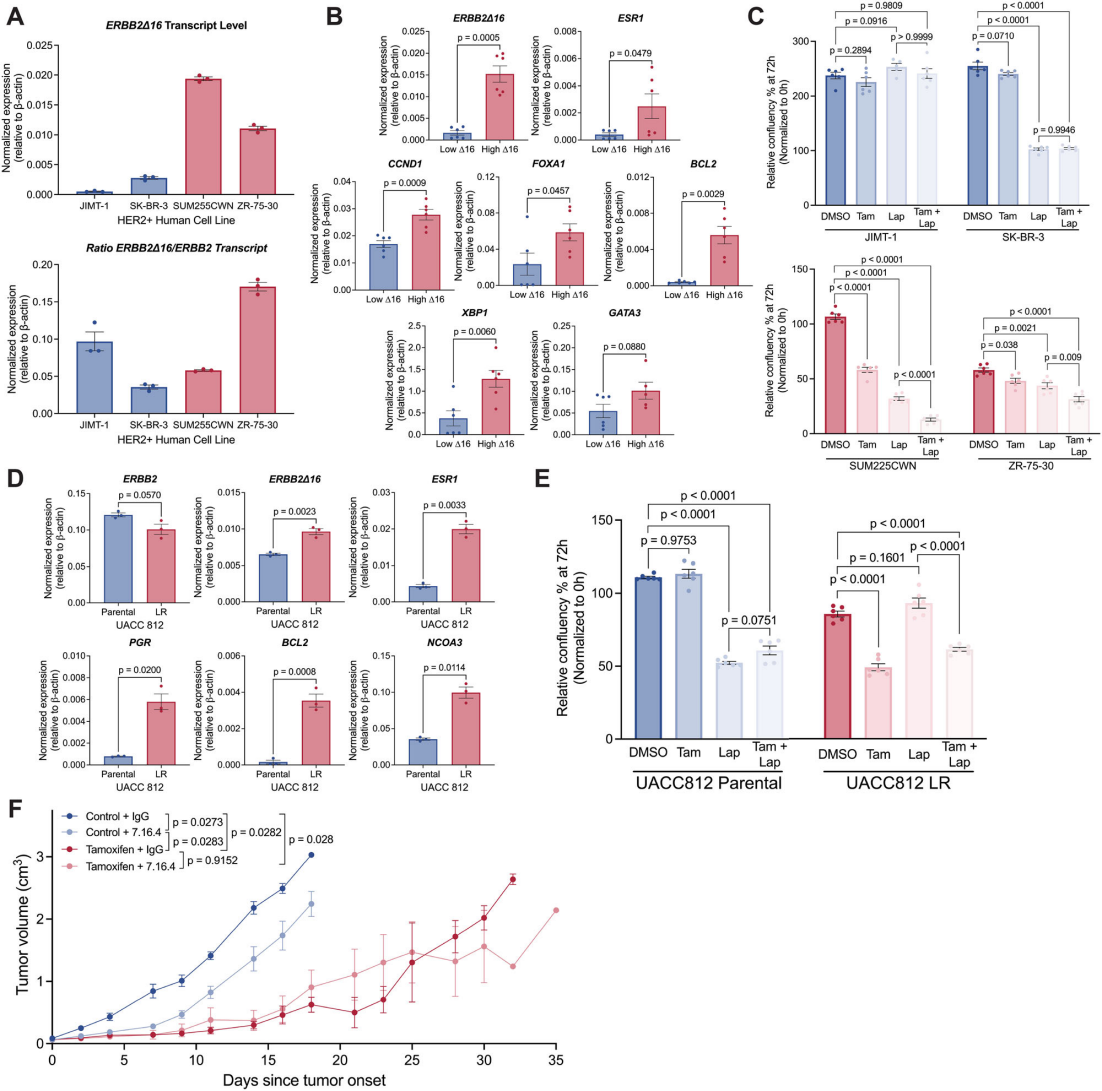
HER2∆16 expression correlates with ER pathway activation and tamoxifen sensitivity in both human breast cancer cell lines and HER2+ murine tumors. **A** qRT-PCR validation of total *ERBB2* and *ERBB2∆16* transcript levels in human HER2+ cell lines, normalized to *β-actin*. Each cell line was tested in triplicate. **B** qRT-PCR for ER-target gene expression normalized to *β-actin* in HER2∆16 low or high cell lines (2 biological groups per cohort, tested in triplicate). **C** Incucyte proliferation of HER2∆16 low (JIMT-1, SK-BR-3) or high (SUM225CWN, ZR-75-30) cells at 72 h (data normalized to confluency at t = 0) after treatment with either dimethylsulfoxide (DMSO), tamoxifen (2 μM) and/or lapatinib (1 μM). Each cell line was tested in sextuplicate. Statistical significance determined by one-way ANOVA with Tukey’s post-hoc test. D) qRT-PCR for ER-target gene expression and *ERBB2∆16* transcript levels in HER2+ parental and lapatinib-resistant (LR) UACC812 cells, normalized to *β-actin*. Each line was tested in triplicate. **E** Incucyte proliferation in parental and lapatinib-resistant (LR) UACC812 cells at 72 h (data normalized to confluency at t = 0) after treatment with either dimethylsulfoxide (DMSO), tamoxifen (2 μM) and/or lapatinib (1 μM). Each cell line was tested in sextuplicate, presented as mean ± SEM. Statistical significance determined by one-way ANOVA with Tukey’s post-hoc test. **F** Tumor outgrowth in orthotopic MMTV-Cre FE16^flx/flx^ tumor xenografts as measured by caliper measurement and calculation of tumor volume starting at first palpable mass. Mice were treated with either standard rodent or tamoxifen (400 mg/kg) diet combined with IgG (control Ab) or 7.16.4 (anti-HER2 Ab) (*n* = 4 per treatment group). Statistical analysis performed by Compare Groups of Growth Curves (CGGC) permutation analysis. All error bars are expressed as mean values  ±  SEM. All statistical analysis by unpaired, two-tailed Student’s *t*-test with Welch’s correction unless otherwise indicated. Source data are provided as a Source Data file.

To evaluate the clinical relevance of the correlation between HER2∆16 expression and ER pathway activation, we profiled a collection of human HER2-positive breast cancer cell lines expressing varying levels of *ERBB2∆16* (Supplementary Fig. 8A)^22^. Stratifying for both total *ERBB2∆16* transcript and expression relative to the full-length isoform, we selected the two cell lines with the highest (SUM225CWN, ZR-75-30) and lowest (JIMT-1, SK-BR-3) HER2∆16 expression for further characterization (Fig. 6A, Supplementary Fig. 8B). Elevated *ERBB2∆16* levels positively correlated with increased *ESR1* expression and ER target gene expression (Fig. 6B). Treatment with the HER2 tyrosine kinase inhibitor, lapatinib, decreased the proliferation of both HER2∆16 low and high cell lines, apart from JIMT-1 cells which are known to be innately resistant^39^. In contrast, tamoxifen treatment significantly impaired proliferation exclusively in HER2∆16 high cell lines, which was strongly augmented by combination with lapatinib (Fig. 6C, Supplementary Fig. 8C).

Human breast tumor microarray samples further demonstrate that *ERBB2∆16* is expressed within both HER2+ /ER- and HER2+ /ER+ tumor subtypes (Supplementary Fig. 9A–B). Variable expression of HER2∆16 was noted in about 55% of clinically scored HER2+ /ER- patients, consistent with what has been previously reported in other clinical datasets (Supplementary Fig. 9C)^18,23^. Interestingly, the prevalence of *ERBB2∆16* expression was higher in patients classified as HER2+ /ER+ with 67% showing variable HER2∆16 expression, suggesting a positive correlation with ER-positivity. Altogether, our findings suggest that expression of the HER2∆16 variant associates with ER expression in breast cancer, although larger clinical sample sizes are required to further explore this correlation (Supplementary Fig. 9C).

The contribution of HER2∆16 to HER2-targeted therapy resistance is contentious^40^. To determine whether tumor cells upregulate HER2 alternative splicing to overcome HER2 inhibition, we established lapatinib-resistant (LR) HER2-positive breast cancer cell lines through serial passaging in increasing concentrations of lapatinib until cells could proliferate at clinically observed concentrations of the drug. In two independent cell lines (UACC812 and SK-BR-3), lapatinib resistance was associated with retained expression of HER2, and increased levels of *ERBB2∆16* compared to the parental strain (Fig. 6D, Supplementary Fig. 10A). Moreover, lapatinib resistant UACC812 cells (UACC812-LR) displayed elevated expression of both *ESR1* and the ER target *BCL-2*, as well as the ER cofactor *NCOA3* and the progesterone receptor (Fig. 6D), while SK-BR-3 LR cells exhibited elevated transcript levels of the ER-responsive genes *BCL-2, MYC, CCND1* and *XBP1* compared to parental cells (Supplementary Fig. 10A). Lapatinib-resistant cells were sensitized to tamoxifen treatment, which reduced their proliferation compared to parental cells (Fig. 6E, Supplementary Fig. 8C).

Using our previously established MMTV-Cre FE16^flx/flx^ tumor allografts we sought to determine their relative sensitivity to either ER and/or HER2 inhibition using the monoclonal anti-HER2 antibody 7.16.4. Akin to previous experiments within this model system, the administration of the tamoxifen diet significantly decreased tumor growth compared to a standard rodent diet (Fig. 4B–C, Fig. 6F). More surprising however, was that tamoxifen was significantly more effective at reducing tumor growth within FE16^flx/flx^ expressing tumors, whereas HER2-inhibition exerted only a modest therapeutic effect (Fig. 6F). Despite the reduction in tumor growth prompted by both tamoxifen and 7.16.4 we failed to observe an additive effect of combining both treatments. These findings are consistent with the role of an ER-associated transcriptional program in mediating resistance to HER2-targeted therapy. Similar findings have been reported previously, where co-expression of ESR1 and Bcl-2 was observed in LR cells and primary patient tumors, in addition to an increased sensitivity to the selective ER-degrader fulvestrant in vitro, although the mechanism driving this transcriptional switch was unknown^17^.

### Estrogen receptor activity in HER2∆16 models is not mediated through *ErbB4* expression

Another HER family member, HER4/ErbB4, has been previously correlated with ER-positivity in breast cancer patients and is known to coregulate ER activity^41,42^. As ErbB4 activity was predicted to be upregulated in ∆16IC tumors relative to EIC controls we sought to evaluate the relative levels of ErbB4 expression across both murine and human HER2∆16 models (Supplementary Fig. 2D). Within murine model systems, ErbB4 transcript expression was detectable in ∆16IC tumors by RNA in situ hybridization (RNA ISH) but remains absent in EIC/FE16^flx/flx^ and FE16^flx/flx^ models (Supplementary Fig. 11A). This is consistent with bulk RNA-sequencing data showing that endpoint ∆16IC tumors display higher *ErbB4* mRNA expression relative to the EIC model (Supplementary Fig. 11B). It is important to note that no similar trend was observed in EIC/FE16^flx/flx^ or FE16^flx/flx^ tumors, with critically low read counts observed in both cases, suggesting this phenotype is limited to the ∆16IC model and not reflected across the multiple HER2∆16 models utilized within this study (Supplementary Fig. 11B). Within human breast cancer cell lines, only the SUM225CWN cell line expressed higher levels of *ERBB4* in combination with elevated *ErbB2∆16* levels, while this was not the case in either the ZR-75-30 or UACC812 cell lines (Supplementary Fig. 11C). Although these data suggest that *ErbB4* expression may be one such mechanism of increasing ER-activity in HER2+ tumors, it is non-uniformly expressed across both murine and human HER2∆16 models and is not directly responsible for the increased ER signaling and luminal identity observed in these tumors.

### The splice factor, SRSF3, is elevated in cell lines expressing high HER2∆16 and correlates with ESR1 expression in HER2+ breast cancer

The complete profile of splice factors which regulate the exclusion of exon 16 in HER2+ breast cancer is not fully understood. Previous studies have shown that the serine/arginine-rich splice factor 3 (SRSF3) is capable of binding to exon 15 of HER2 and promoting exon-16 skipping^43^. We evaluated SRSF3 expression across our various human HER2+ breast cancer cell lines and observed a significant increase in SRSF3 within HER2∆16 high cells, as well as an increase in SRSF1, another member of the SRSF family of splice factors (Supplementary Fig. 10B). SRSF3 transcript levels were similarly increased in SK-BR-3 lapatinib-resistant cells, coinciding with increased HER2∆16 isoform expression and ER-pathway activation (Supplementary Fig. 10A). No such correlation between SRSF1 expression and HER2∆16 isoform expression within lapatinib resistant cells was observed (Supplementary Fig. 10A). Interestingly, using TIMER2.0 to compare the expression of both SRSF3 and ESR1 within the TCGA database we show that SRSF3 expression positively correlates with ESR1 levels within the Luminal A/B and HER2+ breast cancer subtypes (Supplementary Fig. 10C). This association was not observed in basal breast cancer, or in other solid malignancies which have been known to upregulate SRSF3 expression (Supplementary Fig. 10C)^44^. Overall, this confirms that SRSF3 is a major splice factor associated with HER2∆16 expression and significantly correlates with ER-expression uniquely within the Luminal and HER2+ cancer subtypes.

### HER2∆16 tumors sustain HER2 signaling leading to phosphorylation of MAPK and ER

**Fig. 7:**
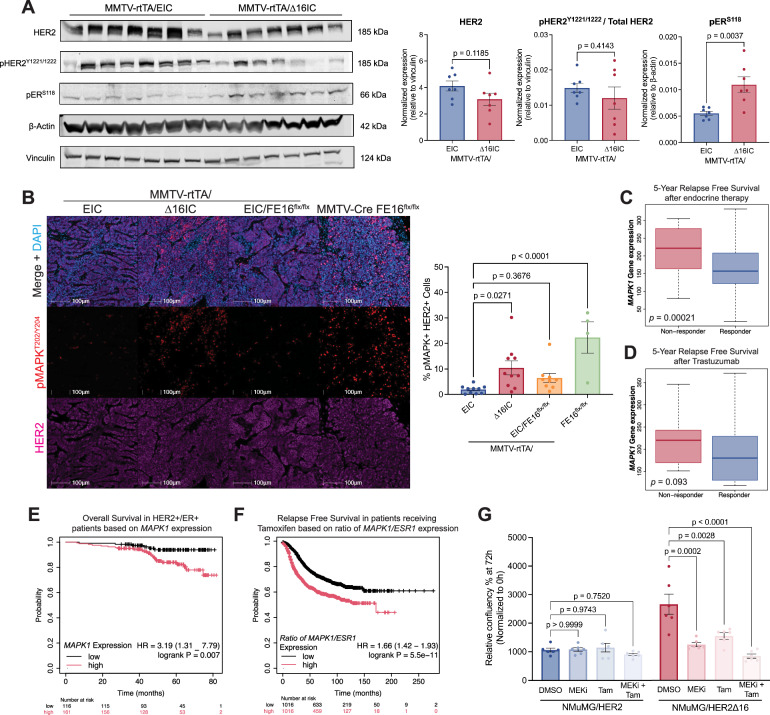
MAPK phosphorylation is associated with HER2∆16 expression and correlates with worse prognosis in HER2+ /ER+ breast cancer. **A** Immunoblots for HER2, pHER2 (Y1221/1222), and pER (S118) from bulk tumor lysates of EIC and ∆16IC mice at tumor endpoint (*n* = 7 tumors per genotype), normalized to vinculin or β-actin. Statistical analysis by unpaired, two-tailed Student’s *t*-test with Welch’s correction. **B** Immunofluorescent staining for HER2 and phosphorylated p42/44 MAPK (T202/Y204) in EIC (*n* = 7), ∆16IC (*n* = 7), EIC/FE16^flx/flx^ (*n* = 7) and FE16^flx/flx^ (*n* = 4) tumors, counterstained with DAPI. Percentage pMAPK+/HER2+ and pMAPK+/HER2- cells were quantified via HALO. Statistical significance determined by one-way ANOVA with Tukey’s post-hoc test. **C** ROC-plotter analysis of MAPK1 gene expression and 5-year relapse free survival in endocrine therapy patient non-responder and responders (*n* = 907, ROC *p *= 2.1e-04). Box plot with center line = median, box = 25th–75th quartile, whiskers = maxima/minima, outliers = open circle. **D** ROC-plotter analysis of MAPK1 gene expression and 5-year relapse free survival in Trastuzumab patient non-responder and responders (*n* = 50, ROC *p* = 0.093). Box plot with center line = median, box = 25th–75th quartile, whiskers = maxima/minima, outliers = open circle. **E** Kaplan–Meier plot indicating the overall survival in patients bearing HER2+ /ER+ breast tumors with low or high MAPK1 expression (*n* = 277, *p* = 0.007). **F** Kaplan–Meier plot indicating relapse free survival in patients receiving tamoxifen with low or high *MAPK1* expression relative to *ESR1*, as determined by the ratio of *MAPK1* to *ESR1* transcript (*n* = 2032, *p*-value = 5.5e-11). **G** Relative confluency of NMuMG HER2 and HER2∆16 cells at 72 h, normalized to t = 0 after treatment with either dimethylsulfoxide (DMSO), tamoxifen (2 μM) and/or the selective MEK inhibitor PD98059 (10 μM). Each cell line was tested in sextuplicate. Statistical significance determined by one-way ANOVA with Tukey’s post-hoc test. All error bars are expressed as mean values  ±  SEM unless otherwise indicated. Source data are provided as a Source Data file.

Expression of HER2∆16 corresponds with constitutive HER2 signaling and has been shown to activate AKT, FAK and SRC family kinases^18,22,23,32^. Bioinformatic analysis of pathways upregulated in the transcriptomes of ∆16IC tumors predicted increased KRAS- and HER2-mediated signaling, consistent with sustained HER2 pathway activity (Supplementary Fig. 2B). Expression of HER2∆16 led to decreased tyrosine phosphorylation of HER2, consistent with previous findings, but increased the activity of ERK (p42/44 MAP kinase) (Fig. 7A-B)^23^. The RAS-MAPK pathway plays a critical role in both HER2 and ER-mediated signaling and confers resistance to HER2 and endocrine therapies^45,46^. Active ERK directly phosphorylates ER at Ser118, facilitating non-canonical signaling independent of estrogen^47^. Accordingly, we observed a significant increase in ER phosphorylation at Ser118 in ∆16IC tumors, corresponding with increased ERK activation (Fig. 7A).

The expression of MAPK, specifically p44 MAPK (MAPK1), corresponds with worse 5-year relapse free survival in breast cancer patients treated with endocrine therapy (Fig. 7C). Patients expressing high levels of MAPK1 also appear to have slightly worse 5-year relapse free survival when treated with the anti-HER2 monoclonal antibody Trastuzumab, although this was not significant (Fig. 7D). Within HER2+/ER+ patient tumors, high MAPK1 expression significantly correlates with worse overall survival (Fig. 7E). This is particularly evident in cases when MAPK1 expression is high relative to ESR1, where tamoxifen treated patients demonstrate significantly worse relapse-free survival, which may be attributed to the estrogen-independent ER-signaling driven by MAPK1 phosphorylation, which is known to confer tamoxifen resistance (Fig. 7F)^46,48^. Using murine NMuMG cell lines expressing either the full-length HER2 or HER2∆16 isoforms, we show that targeting MAPK expression through inhibition of the upstream kinase MEK, effectively inhibits cell growth specifically in HER2∆16 expressing cells (Fig. 7G). Furthermore, we observe an additive effect when combining MEK inhibition with tamoxifen, suggesting this combination is highly effective in reducing HER2∆16 cell proliferation (Fig. 7G). Collectively, these data suggest that sustained HER2 signaling via HER2∆16 promotes activation of MAPK/ERK, which in turn mediates ER phosphorylation. This provides an alternate mechanism of non-canonical ER signaling within HER2∆16 tumors, promoting HER2+/ER+ tumor progression. MAPK pathway activation, driven by constitutive HER2∆16 signaling, may negatively impact therapeutic response within HER2∆16-expressing tumors, conferring estrogen-independence and resistance to ER-antagonists such as tamoxifen. However, this signaling axis may be effectively targeted through combination treatment approaches incorporating MEK/MAPK inhibitors with endocrine therapies.

Taken together, our findings suggest that HER2∆16 is a potent driver of mammary tumorigenesis which promotes luminal cell fate within HER2+ tumors. Expression of HER2∆16 increases ER expression and activates signaling pathways including RAS-MAPK to sustain ER-dependent signal transduction and promote epithelial cell differentiation. Clinically, expression of MAPK within HER2+/ER+ tumors leads to worse overall prognosis and response to endocrine therapy but can be effectively targeted in HER2∆16-expressing cell lines via MEK inhibition and synergizes with tamoxifen in vitro.

**Fig. 8:**
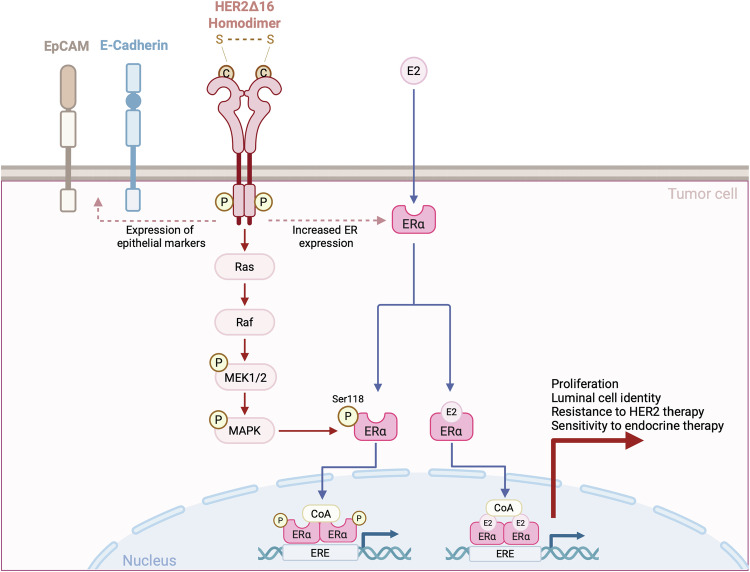
HER2∆16 directs luminal cell identity and estrogen receptor signaling in HER2+ breast cancer. Schematic illustrating the role of HER2∆16 in facilitating the HER2/ER signaling axis and sensitivity to endocrine therapy. Expression of HER2∆16 promotes constitutive HER2 signaling which activates the Ras/MAPK signal transduction pathway and expression of epithelial cell surface markers, EpCAM and E-cadherin. Active MAPK mediates ER-phosphorylation enabling non-canonical signaling independent of estrogen stimulus, further directing luminal cell identity and proliferation in HER2+ cells. As HER2∆16 cells may sustain ER-signaling to overcome HER2 inhibition, this confers sensitivity to endocrine therapies which can be exploited to treat resistant HER2-positive breast tumors. Created in BioRender. Muller, W. (2026) https://BioRender.com/d877crt.

HER2∆16 expression is sufficient to promote HER2+/ER+tumorigenesis in GEMMs and confers sensitivity to the ER antagonist tamoxifen. In human cell lines, expression of HER2∆16 drives an ER-associated transcriptional program which is increased during acquisition of resistance to lapatinib, sensitizing HER2+ cells to endocrine therapy. These results offer valuable insights into a mechanism by which HER2+ tumors evolve to acquire luminal molecular features and reveal an ER-dependent survival mechanism that facilitates resistance to anti-HER2 therapies (Fig. 8).

## Discussion

Co-expression of ER and HER2, occurring in up to 10% of breast cancers, contributes to the biological heterogeneity of HER2-positive tumors and alters their clinical behavior, including response to treatment^49^. However, our understanding of these cancers has been limited by the absence of physiologically relevant models^24^. Furthermore, the molecular mechanisms which permit HER2+ cells to acquire luminal features and upregulate both ER expression and transcriptional activity remain poorly understood. Here, using a panel of GEMMs, we revealed that HER2/ErbB2∆16 expression in the mammary epithelium promotes ER expression and an ER-dependent transcriptional program, correlating with an aggressive, luminally differentiated phenotype and tamoxifen sensitivity (Figs. 1–2, Supplementary Fig. 1).

We further demonstrated the ability of HER2∆16 to promote a luminal transcriptional program by developing a conditional floxed exon 16 (FE16^flx/flx^) model, where ErbB2∆16 is expressed under its endogenous promoter and sufficient to promote HER2+/ER+ tumorigenesis (Fig. 3). Within FE16^flx/flx^ tumors, which retain luminal cytokeratin expression as well as upregulation of *Esr1*, we detected genomic *ErbB2* amplification as seen in patients (Fig. 3, Supplementary Fig. 6). This is consistent with previous studies where tumors expressing an activated rat ErbB2/Neu oncogene under the endogenous *ErbB2* promoter amplified the transgene^31^. Overall, these data suggest that a threshold level of ErbB2 expression required for transformation of mammary epithelial cells is met in MMTV-driven and doxycycline-inducible systems but requires *ErbB2* gene amplification when transcription is under the control of the endogenous promoter. The murine *ErbB2* amplicon closely recapitulates that of human *ERBB2*-amplified breast cancer, including co-amplification of *Stard3* and *Grb7* which contribute to the growth of HER2+ breast cancer cells^33^. This may indicate an additional requirement for co-expression of *Grb7*, *Stard3*, and potentially other co-amplified genes to facilitate transformation and tumor progression^50–52^.

Additionally, we show that co-expression of both HER2/ErbB2 isoforms, as seen in patients^18^, causes tumors to retain the luminal differentiation and ER-driven transcriptional profile of tumors expressing HER2∆16 (Fig. 5, Supplementary Fig. 7). This confirms the dynamic influence of HER2∆16 on intracellular signaling, as human carcinomas often co-express both these HER2 isoforms within the primary tumor^18^. However, the threshold of HER2∆16 expression required to polarize this shift, or the exact downstream molecular pathways catalyzing this change remains to be explored. The delayed onset of these tumors relative to those expressing full length HER2 alone may be linked to their more differentiated phenotype, which is typically associated with less aggressive features^53^. However, EIC/FE16^flx/flx^ tumors show an increased incidence of metastasis similar to ∆16IC mice (Supplementary Fig. 1, 7), possibly reflecting tumor-extrinsic effects of HER2∆16 on the immune system (Fig. 4) and vasculature which ultimately allow tumors to overcome this initial delay^23^.

We found that increased *ERBB2∆16* expression in human HER2+ breast cancer cell lines, or a higher proportion of *ERBB2∆16* relative to full length *ERBB2*, correlated with increased expression of *ESR1*, ER target genes, and ER cofactors (*FOXA1, NCOA3* and progesterone receptor) (Fig. 6). This was reaffirmed in patient tumor microarray samples where expression of HER2∆16 was higher in patients expressing both HER2 and ER as opposed to HER2+ /ER- patients (Supplementary Fig. 9). HER2+ cells with acquired resistance to HER2 tyrosine kinase inhibition showed tandem upregulation of HER2∆16 and *ESR1*, with concomitant sensitization to endocrine therapy (Fig. 6, Supplementary Fig. 10). Collectively, these data suggest that alternative *ERBB2* splicing may allow HER2+ breast cancer cells to overcome HER2 inhibition through HER2∆16-dependent ER signaling. Accordingly, analysis of samples from a recent phase II trial showed that ER and its target BCL-2 were upregulated in breast cancer biopsies after 2 weeks of neoadjuvant lapatinib treatment, with ~20% of tumors converting from ER-negative to ER-positive, demonstrating that expression of ER and ER-target genes acts as an escape mechanism for HER2-inhibition^17^.

The complete profile of splice factors which regulate HER2∆16 expression remains poorly understood. However, here we provide evidence for the role of SRSF3, specifically in promoting ER-positivity within Luminal and HER2+ breast cancer (Supplementary Fig. 10). Although we believe this impact of SRSF3 is predominantly through exclusion of exon 16, its wide-ranging effect on alternative splicing merits further exploration. Additionally, it’s relationship with fellow SRSF-family member SRSF1 should be considered, as these two show mutual dependency and both appear to be upregulated in HER2∆16 high cell lines (Supplementary Fig. 10)^54^. Future studies will aim to determine whether overexpression of SRSF3 alone or in combination with SRSF1 is sufficient to drive HER2∆16 splicing and ER-positivity within human breast cancer cell lines.

Previous studies have established HER2∆16 as a potent oncogenic driver in a multitude of cancers arising in the breast, lung and gastric epithelium which are characterized by hyperactive HER2 signaling through stabilized expression of HER2∆16 homodimers at the cell surface^19,21,23,40^. Estrogen receptor upregulation may serve as a compensatory process to mitigate this imposed reliance on sustained HER2-signaling. This further highlights the necessity of model systems which faithfully recapitulate HER2+ /ER+ breast cancer, as modeling ER-compensation in vitro remains challenging due to frequent loss of ER expression within cell lines, the inadequacy of two-dimension cultures in recapitulating the in vivo microenvironment and fluctuating hormonal levels found in patients^24^. Furthermore, the presence of a complete tumor immune microenvironment is an important advantage of GEMMs compared to the majority of ER+ breast cancer models, which are xenografts relying on the use of immunodeficient mice^24^. That being said, we acknowledge there are limitations of these transgenic models when establishing the human relevance of HER2∆16. These transgenic systems, although valuable in modeling key molecular pathways are unable to fully mimic human disease and the genetic heterogeneity of HER2+ tumors. Primarily, as HER2∆16 expression varies between patients, it is difficult to determine the critical threshold of HER2∆16 expression required to illicit intracellular or microenvironmental changes in HER2+ tumors. Furthermore, HER2+ breast cancer often coincides with amplification of the *ERBB2* locus, making it difficult to uncouple the effects of HER2∆16 expression and other genetic amplification events. However, the MMTV-Cre FE16^flx/flx^ model which can recapitulate *ErbB2* amplification under the endogenous promoter offers one avenue for future investigation. Generation of blocking antibodies or antisense oligonucleotides specific for HER2∆16 would allow us to define the contribution of HER2∆16 in HER2+ tumor phenotypes irrespective of *ERBB2* amplification and functionally assess its relevance across solid malignancies using both murine models and patient derived xenografts which better reflect the genetic landscape of HER2+ tumors.

Despite these limitations, there remains an unmet need for preclinical GEMMs that accurately model HER2+/ER+ disease, as this tumor subtype is poorly represented by the current available model systems. In this respect, the MMTV-Cre FE16^flx/flx^ model is indispensable in its ability to model the HER2+/ER+ breast cancer subtype to further understand this signaling axis. The ability to readily allograft FE16 tumors into immune-competent hosts, while avoiding reliance on the viral MMTV-LTR promoter which is modulated by steroid hormones, establishes a preclinical platform to evaluate therapeutic approaches in vivo, as we demonstrate using both tamoxifen and the anti-HER2 antibody 7.16.4 (Fig. 4, Fig. 6F)^31^. Although the contribution of HER2∆16 to HER2-targeted therapy resistance remains contentious, we show that MMTV-Cre FE16^flx/flx^ display only a modest response to HER2-inhibition, and are more effectively treated through antagonizing ER-pathway activity (Fig. 6F)^40^. Additionally, we were unable to observe an additive effect of combining both tamoxifen and 7.16.4 in vivo. Although we hypothesize that this is due to the increased sensitivity of HER2∆16 to ER-antagonism, which is sufficient to reduce tumor growth and therefore cannot be further augmented by HER2-inhibition, we cannot exclude the fact that a tumor co-expressing both HER2 isoforms may be more receptive to ER/HER2 co-inhibition. Subsequent experiments may benefit from establishing MMTV-Cre FE16^wt/flx^ allografts, which would allow us to examine the contribution of both the full-length and HER2∆16 isoforms in dictating therapeutic response, and better recapitulate human tumors. In the future, the MMTV-Cre FE16 model can be leveraged to evaluate clinically relevant treatment strategies such as the use of immunotherapy in combination with ER-inhibition as well as the use of CDK4/6 inhibitors, both of which are currently in clinical trials for the treatment of HER2+ /ER+ breast cancer^55,56^.

Complex bi-directional crosstalk between ER and HER2 pathways may underlie tumor progression during tamoxifen therapy (Fig. 4), representing a potential transition to hormone insensitivity through increased reliance on HER2 or Ras/MAPK-driven non-canonical ER signaling. In this regard, our observation of elevated MAPK/ERK signaling and ERK-dependent ER phosphorylation on Ser118 in HER2∆16-expressing breast cancers (Fig. 7) raises an interesting possibility, since this promotes non-canonical, ligand-independent ER signaling while conferring resistance to anti-estrogens^48^. While this demonstrates one mechanism of HER2-ER crosstalk that is influenced by HER2 isoform expression, considering that ER transcript expression is also increased in HER2∆16 tumors, MAPK activity alone cannot fully explain this phenotype. The mechanisms driving increased ER expression in HER2∆16 tumors remain to be elucidated however, the divergent transcriptional profiles in either HER2 isoform may implicate epigenetic changes which influence cell differentiation (Fig. 1).

Although we mainly investigate the role of HER2∆16 on ER-signaling, it remains intriguing how the splice isoform promotes both luminal and EMT features within cancer cells, such as metastasis. Moreover, a dominant effect seems to occur in conditions where both full-length and HER2Δ16 are expressed which suggests that even partial expression of HER2∆16 is sufficient to dictate phenotypic outcomes in HER2+ tumors (Fig. 5, Supplementary Fig. 6, 7). The duality of HER2∆16 in promoting both luminal and mesenchymal features may again point towards a multifocal role of MAPK in sustaining ER-signaling while simultaneously directing cell migration. This may be facilitated via modulation of the tumor microenvironment through diverse pathways such as tumor vascularization or cell surface signaling events through epithelial-cadherin or β1-integrins (Supplementary Fig. 2B)^57^. We have previously reported disruptions in tumor vasculature as a consequence of HER2∆16 expression within our model systems which may aid in promoting cancer cell migration however it is unknown whether this is directly due to the observed increased MAPK pathway activity or other molecular mechanisms^23^.

Overall, our findings suggest a role of HER2∆16 in promoting an ER-dependent transcriptional program and a luminal-like cellular state in HER2-positive breast cancer. Thus, while HER2∆16 is known to promote an aggressive tumor phenotype and immune-cold microenvironment, it simultaneously confers sensitivity to endocrine therapy targeting ER (Fig. 8). Although the exact mechanisms by which HER2∆16 promotes luminal cell identity remain to be elucidated, these results offer valuable insights into how HER2+ tumors may promote a switch to a luminal cellular identity within either primary tumors or as acquired by lapatinib resistant cells. Furthermore, the use of HER2∆16 as a tool to model HER2+/ER+breast cancer provides an invaluable preclinical model which can be exploited to further interrogate this understudied signaling axis to better understand HER2+/ER+ disease.

## Materials and methods

### Transgenic mice

All animal experiments in this study comply with the ethical standards outlined by the Canadian Council of Animal Care (CCAC) and approved by the Animal Care Committee of McGill University. Mice were maintained within the specific pathogen-free (SPF) animal research facility at the Goodman Cancer Institute, housed in autoclaved cages with *ad libitum* access to food and water as well as sufficient nesting and enrichment materials. Cages were kept on ventilated racks under a 12 h light cycle at a temperature of 20–24 °C and relative humidity of 45-65%.

The TetO-ErbB2/Her2(human)-IRES-Cre (EIC), TetO-ErbB2∆16IC/HER2∆16(human)-IRES-Cre (∆16IC), MMTV-Cre and MMTV-rtTA (MTB) have been published previously^22,23,58,59^. EIC or ∆16IC mice were mated as hemizygotes to the MTB strain to generate bigenic animals with doxycycline-inducible HER2 expression. The genetic background of experimental mice was validated by PCR using primers specific for MTB, HER2/HER2∆16 and Cre transgenes (Supplementary Table S1)^22,23,60^. At 8-12 weeks of age, doxycycline (2 mg/mL, Wisent) was administered to bigenic mice via the drinking water to activate the reverse tetracycline transactivator (rtTA) transgene and drive expression of EIC or ∆16IC downstream from the TetO promoter specifically in the mammary epithelium.

The HER2flxExon16 (FE16) transgenic strain was developed by gene targeting of endogenous *neu/erbb2* to contain exon 16 flanked by loxP sites^23^. FE16 mice were maintained as either hemi- or homozygotes and interbred with mice expressing Cre recombinase under the transcriptional control of the MMTV promoter (MMTV-Cre) leading to mammary-specific excision of exon 16. To generate the EIC/FE16^flx/flx^ strain, MMTV-rtTA/EIC mice were interbred and maintained as homozygotes for FE16 allowing for simultaneous excision of murine *Erbb2* exon 16 with the expression of the human HER2 oncogene when induced by doxycycline administration.

All animals were maintained on a pure FVB/N background (Charles River, FVB/Ncrl; Strain code: 207). Experimental mice were monitored for tumor formation by weekly palpation and caliper measurements post-doxycycline induction. Tumor onset was defined as the first palpable mass and mice were allowed to reach clinical endpoint as defined by the animal care guidelines as a single tumor of 2.5 cm^3^ or a total volume of 6 cm^3^ for multifocal tumors. Maximal tumor size/burden were not exceeded during this study. Mice were weighed at dissection and tumors were removed and weighed to calculate tumor burden. Tumor tissue and lungs were formalin-fixed for 24–36 h and paraffin-embedded, and tumor pieces were flash frozen in liquid nitrogen and stored long term at −80 °C.

### Orthotopic tumor transplant and in vivo tamoxifen treatment

Endpoint tumors dissected from transgenic mice were dissociated using a tissue chopper as previously described^61^. Briefly, cells were suspended in a solution of Dulbecco’s Modified Eagle Medium (DMEM) (Wisent 319-005-CL) containing collagenase B (2.4 mg/mL, Roche 11088831001) and dispase II (2.4 mg/mL, Roche 4942078001) for 1.5 hours at 37 °C in a rotating incubator. The suspension was then centrifuged for 10 min at 200 × *g* before being washed with PBS/0.01 M EDTA. Red blood cells were lysed for 10 min at room temperature using ammonium-chloride-potassium (ACK) lysis buffer (0.15 M ammonium chloride, 0.1 M potassium bicarbonate, 0.1 mM EDTA). Cells were resuspended in PBS and 0.5  × 10^6^ cells were injected into the mammary fat pad of 8–10-week-old female syngeneic FVB/N mice (Charles River, FVB/Ncrl; Strain code: 207) and monitored for tumor formation.

Mice were kept on a standard rodent diet (Teklad, Inotiv 2920X) to allow appropriate recovery post-surgery before half of the group were switched to the *ad libitum* tamoxifen diet (400 mg/kg, Inotiv TD.130860). Tumors were monitored 2–3 times weekly post-onset and measured with a caliper. Mice were weighed at dissection and tumors were removed and weighed to calculate tumor burden at clinical endpoint.

For in vivo therapeutic studies utilizing tamoxifen in combination with HER2-inhibition, female mice were randomly assigned to treatment groups and monitored for tumor growth. Treatments began once tumors reached a size of 5 × 5 mm (approximately 65 mm^3^), with half the cohort being kept on a standard rodent diet (Teklad, Inotiv 2920X), and the other switched to the *ad libitum* tamoxifen diet (400 mg/kg, Inotiv TD.130860). 7.16.4 mAb (Bio X Cell, BE0277) and a mouse isotype-matched control antibody IgG1 mAb (Bio X Cell, BE0083) were administered in doses of 100 μg via intraperitoneal injection, 2 times a week and monitored via three-times weekly caliper measurements. All therapeutic treatments used received ethical approval by CCAC (AUP 5518).

### Lung metastasis quantification

Formalin-fixed paraffin-embedded (FFPE) lung sections were stained with hematoxylin and eosin and scanned at 20X magnification using an Aperio-XT slide scanner (Leica Biosystems). HALO software (Indica Labs, v3.5.3577) was used to quantify the area of metastatic lesions and show as a percentage of total lung area.

### Mammary gland wholemounts

No. 4 inguinal mammary glands from FE16 transgenic mice were collected at 2-, 6- or 8-month timepoints and spread on glass before overnight fixation in acetone^62^. Mammary glands were then incubated in Harris Modified Hematoxylin stain (Fischer Chemical, SH30-4D) overnight and destained in 1% HCl in 70% ethanol. Mammary glands were washed with ethanol and cleared in xylene for 24 hours before mounting with ClearMount (StatLab, MMC0126).

### Primary cell culture

Cells were cultured in a humidified, 5% CO_2_, 37 °C incubator in complete media. UACC812 (ATCC, CRL-1897), SK-BR-3 (ATCC, HTB-30), JIMT-1 (Accegen, ABC-TC504S) and NMuMG (ATCC, CRL1636) cells were grown and maintained in DMEM (Wisent, 511-016-UG), supplemented with 10% fetal bovine serum (FBS) and 1% penicillin/streptomycin (Wisent, 450-200 EL), as well as 5 µg/ml human insulin. ZR-75-30 (ATCC, CRL-1504) cells were maintained in RPMI 1640 (Wisent, 350-060 CL), supplemented with 10% FBS and 1% penicillin/streptomycin. SUM225CWN cells (BioIVT, HUMANSUM-000301) were maintained in HAM’s F-12 (Wisent, 318-011 CL), supplemented with 5% FBS, 10 mM HEPES, 1 µg/ml hydrocortisone and 5 µg/ml insulin. NMuMG/HER2 and NMuMG/HER2∆16 cell lines were transfected with either pMSCV-HER2 or pMSCV-HER2∆16 plasmids using Genejuice (Novagen 70967), and selected with 2 μg/ml puromycin (Clontech 631305) as published previously^22^. UACC812 and SK-BR-3 lapatinib resistant (LR) cells were established by exposing parental cells to increasing concentrations of lapatinib over multiple passages, until no further cell death was observed, and maintained in 2 µM lapatinib in culture. Cell cultures were routinely monitored for contamination, passaged once they reached 80–90% confluency and used at early passages. Regular testing of cells for mycoplasma using the MycoAlert Kit (Lonza, LT07-118) confirmed that all cell lines used in this study were negative for mycoplasma contamination.

### Transcriptomic analysis

RNA from flash frozen tumor tissue using FavorPrep Tissue Total RNA Mini Kit (Favorgen, FATRK001) according to the manufacturers protocol. Total RNA concentration and purity was assessed using a Nanodrop 2000 (Thermo Fisher Scientific, ND2000CLAPTOP). RNA sequencing was performed by Novogene (Beijing, China) on RNA from EIC and ∆16IC tumors at endpoint. Differential expression analysis was performed, and volcano plots were generated, using the DEGseq2 R package (2_1.6.3) with *p*-values adjusted using Benjamini and Hochberg’s method^60^. Gene set enrichment analysis (GSEA) was performed using individual gene counts from each genotype and mapped against the MSigDB mouse gene set collection (Broad Institute, GSEA v 4.3.3). All gene sets shown have a *p* < 0.05. Upregulated genes in ∆16IC tumors were then analyzed by EnrichR, using genes with a minimum 2-fold change difference between genotypes and a minimum of 50 reads^63–65^. Transcription factor activity scores for each sample were obtained using DecoupleR and a list of activated or deactivated Esr1 target genes were generated^26^.

### Quantitative reverse transcriptase PCR

Total RNA extracted from cultured cells or flash frozen tumors was reverse transcribed into cDNA using the TransScript All-in-One First-Strand cDNA Synthesis SuperMix for qPCR (Transgen Biotech, AT341-01). Real time quantitative PCR (qRT-PCR) was performed using the LightCycler 480 Sybr Green 1 MasterMix (Roche, 04887352001), run on the Lightcycler 480 instrument (Roche) and analyzed using the corresponding software (Lightcycler 480 SW, v1.5.1.62). Relative expression (RQ = 2^∆Ct^) and normalization was done using β-actin. Primer sequences are in Supplementary Table S2.

### Genomic PCR

Genomic DNA was extracted from either endpoint tumors, mammary gland or spleens from transgenic mice using the Monarch Spin gDNA Extraction Kit (New England Biolabs, T3010S) as per the manufacturer’s protocol. Total DNA concentration and purity was assessed using a Nanodrop 2000 (Thermo Fisher Scientific, ND2000CLAPTOP). qPCR was performed using the same primers and conditions as previously described^23,66^. Reaction mixture of diluted DNA (6.25 ng/µL), Sybr Green 1 MasterMix (Roche, 04887352001), and primers for murine *ErbB2* or *Gapdh* were run in duplicate per biological replicate in the Lightcycler 480 (Roche). Gapdh was used to normalize all the samples and the ∆C_t_ of the spleen was used to normalize the copy number of the mammary gland and tumor samples.

### Protein extraction and immunoblotting

Tumor tissue was flash-frozen and crushed using a mortar and pestle under liquid nitrogen. The powdered tissue was suspended in RIPA lysis buffer (10 mM Tris-HCl pH 8, 1 mM EDTA, 1% NP-40, 0.1% sodium deoxycholate, 0.1% SDS, 140 mM NaCl) containing protease inhibitors; 0.5 mM AEBSF (Santa Cruz, sc-202041), 25 mM beta-glycerophosphate (Sigma, G5422), 1 mM sodium orthovanadate (BioShop, SOV664), and 10 mM sodium fluoride (Sigma, S7920). Samples were left to rotate at 4 °C for 1.5 h before being cleared by centrifugation at 4 °C, 15,000 × *g* for 10 min. Protein concentrations were determined from the resulting lysate by Bradford assay (Bio-Rad, 5000006) and 40 µg of total protein was analyzed by SDS-PAGE followed by fluorescent immunoblotting using the Li-COR Odyssey system. Quantification was performed using the associated software, Image Studio Lite (Li-COR Biosciences, v5.2.1). Primary and secondary antibodies are detailed in Supplementary Table S3. Target proteins were normalized to loading controls on the same membrane.

### Multiplex fluorescent immunohistochemistry

Staining was performed on 4 µm thick formalin-fixed paraffin-embedded tumor sections taken at endpoint or human tumor microarray samples purchased from US Biomax (BR1504b)^67^. Sections were deparaffinized using successive 3 min washes in 100% xylene, before hydrating using decreasing ethanol concentrations (3×100% EtOH, 95% EtOH, 70% EtOH). Antigen retrieval was performed using either pH 6.0 citrate buffer (Vector Laboratories, H-3300-250) or pH 9.0 EDTA buffer (Vector Laboratories, H-3301-250) by incubating in a pressure cooker for 10 min. Sections were then incubated in 3% hydrogen peroxide and blocked in casein-based buffer (Vector Laboratories, SP-5020) for 5 min. Primary antibodies were diluted in 2% bovine serum albumin (BSA) dissolved in 0.05% TBS-T and incubated for 30 min at room temperature in a humidity chamber. A complete list of primary antibodies used, and their working concentrations can be found in Supplementary Table S3. Slides were then washed in 0.1% TBS-T three times and incubated in ImmPRESS HRP Polymer secondary antibodies (Vector Laboratories, VECTMP745250 or VECTMP745150) for 30 min at room temperature in a humidity chamber. Signal detection was performed using OPAL fluorophores with varying excitation/emission spectra (Akoya Biosciences, OPAL 520: OP-001001, OPAL 570: OP-001003, OPAL 620: OP-001004, OPAL 690: OP-001006) in a working solution containing tyramide signal amplification substrates and incubated for 10 min at room temperature in a humidity chamber. For primary antibodies raised against ER*α* (sc-542), signal amplification was performed using Aluora 555 spatial amplification dye (Invitrogen, AS555HRP). Slides were then antigen retrieved, and the process was repeated for up to 4 colors per section. Samples were counterstained with DAPI (Sigma, D9542) and mounted for imaging using Epredia Immu-Mount (Thermo Fisher, 9990402). For traditional immunohistochemistry, following secondary antibodies, the sections were incubated in 3,30 -diaminobenzidine (DAB) (Cell Signaling, 8059) for the empirically determined time. Slides were then counterstained with hematoxylin for 30 s before dehydrating using increasing ethanol concentrations (70% EtOH, 95% EtOH, 3×100% EtOH) and three 100% xylene washes. Slides were mounted using Epredia Cytoseal XYL (Thermo Fisher, 8312-4).

Imaging was performed using a Zeiss AxioScan Z1 digital slide scanner, and analyzed using HALO software (Indica Labs, v3.5.3577) using the algorithms “HighPlex FL v4.0.4” for immunofluorescent staining or “Multiplex IHC v2.3.4” for immunohistochemistry. The entire tissue section was analyzed for all staining experiments. Staining intensity was quantitatively defined on HALO using an algorithm recognizing the individual fluorescent intensities (scored at 0, +1, +2 and +3) of single cells to determine a relative H-score representing the degree of staining intensity across the entire section.

### RNA in-situ hybridization

RNAscope in situ hybridization was performed on paraffin-embedded tumor sections from endpoint lesions using RNAscope 2.5 HD Assay-RED Kit (ACD, 322360) as per the manufacturer’s protocol. Probes against murine *Esr1* (ACD, 478201), murine *ErbB4* (ACD, 318721) and the control, *Ppib* (ACD, 313911) were used, and the protocol was followed with fluorescent immunohistochemistry as described above. Imaging was performed using a Zeiss AxioScan Z1 digital slide scanner, and analyzed using HALO software (Indica Labs, v3.5.3577).

### BaseScope

The BaseScope assay for human wild-type Her2 (ACD, 70112) and human Her2∆16 (ACD, 701111) was carried out as per the manufacturer’s protocol using the BaseScope Detection Kit v2 (ACD, 323910). NMuMG, ZR-75-30, JIMT-1, SK-BR-3 or SUM225CWN cells were seeded at a density of 5 × 10^4^ per well in a Lab-Tek II Chamber Slide (Thermo Scientific, 154526) overnight at 37 °C before being fixed with 10% neutral buffered formalin, dehydrated with increasing concentrations of ethanol and stored at −20 °C as per the manufacturer’s recommendations. Cells were rehydrated using decreasing ethanol concentrations and covered with 3% hydrogen peroxide for 10 min at room temperature. Cells were permeabilized for 10 min using protease III (ACD) and probes were left to hybridize for a period of 3 hours at 40 °C. Signal was detected using Fast-Red alkaline phosphatase substrate (ACD, 323900) and slides were scanned using the Zeiss AxioScan Z1 digital slide scanner. Similarly, Basescope was performed on human tumor microarray samples purchased from US Biomax (BR1504b) following the same protocol as outlined above following deparaffinization and hydration with decreasing ethanol concentrations (3×100% EtOH, 95% EtOH, 70% EtOH).

### IncuCyte cell proliferation assays

Tamoxifen (MedChem Express, HY-13757A), Lapatinib (MedChem Express, HY-50898) and PD98059 (MedChem Express, HY-12028) were dissolved in DMSO to an initial concentration of 2 mM, 1 mM or 10 mM respectively. For proliferation assays, 1000 NMuMG cells, 5000 SK-BR-3, JIMT-1, SUMM225CWN and ZR-75-30 cells, and 10,000 UACC812 parental or LR cells were seeded per well in sextuplicate in 96-well optical-bottom plates (Nunc, 167008). After 24 h, cells received either a DMSO control, the selective MEK inhibitor PD98059 (10 µM), tamoxifen (2 µM) and/or lapatinib (1 µM). Imaging was performed using the IncuCyte S3 system (ESSEN BioSciences). Cells were imaged at 10x magnification every 6 hours over a period of 48–72 h with 4 images taken per well, per timepoint. Confluence was determined using the IncuCyte S3 Analysis software (ESSEN BioSciences, v2019A), and percentage confluence relative to the initial (0 h) timepoint was calculated. Data were plotted for each time course as the mean ± SEM.

### Analysis of transcriptomic data from publicly available datasets

Receiver Operating Characteristic (ROC) plots were generated from ROC plotter (https://rocplot.org) using Breast Cancer datasets identified in the Gene Expression Omnibus (GEO) (http://www.ncbi.nlm.nih.gov/gds). The GEO platform IDs used included, “GPL96” (for HG-U133A), “GPL570” (for HG-U133 Plus 2.0), “GPL571” (for HU-U133A_2), the keywords “breast”, “cancer” and “therapy” and datasets with fewer than 30 samples were excluded (*n*  =  3104)^68^. These datasets were selected as they are widely used and utilize the same probes for measuring the same genes. “ROC Plotter for breast cancer” tool was selected and *MAPK1* was entered as the gene symbol. ROC plots were created for relapse-free survival (RFS) to any endocrine therapy or the anti-HER2 therapy, Trastuzumab. Kaplan-Meier Plotter (https://kmplot.com) was used to assess the prognostic value of *MAPK1* in terms of both overall and relapse-free survival. HER2+ /ER+ and tamoxifen treated patient samples were stratified into two groups, high and low, based on median cut-offs. The two patient cohorts were compared via Kaplan–Meier survival plots, and the hazard ratios, calculated using the Cox proportional-hazard model with 95% confidence intervals (CIs), and log-rank *p* values were displayed on the figure^69^.

To correlate *SRSF3* expression with *ESR1* levels across various solid malignancies, we used the ‘cancer exploration gene correlation module’ from the web tool TIMER2.0 (http://timer.cistrome.org). Results were analyzed in an unbiased manner by considering all algorithms integrated, including TIMER, xCell, MCP-counter, CIBERSORT, EPIC, and quanTIseq. Results were provided as a heat map table showing the tumor purity-adjusted Spearman correlations between *SRSF3* and *ESR1* estimated by all six algorithms, with red indicating a statistically significant positive association, blue indicating a significant negative association, and gray a nonsignificant result^70^. Linear regression models were generated for the relevant cancer datasets with both the Rho and *p*-values displayed on the figure.

### Statistical analysis

All graphs and statistical analyses, unless otherwise indicated, were generated using GraphPad Prism 9.0 software and compiled on Adobe Illustrator (v25.2.3). Statistical significance was determined by unpaired, two-tailed Student’s t-tests, one-way ANOVA with Tukey’s post-hoc tests for multiple comparisons, and Kaplan–Meier analysis with logrank tests (Mantel–Cox), as appropriate. Data is presented as the mean ± standard error of the mean (SEM), with *p* values less than 0.05 considered to be statistically significant. The methods for statistical tests and sample sizes (*n*) are indicated in figure legends. Exact *p*-values (for *p  <  0.05, **p  <  0.01, ***p  <  0.001, ****p  <  0.0001) are indicated in all display figures.

### Reporting summary

Further information on research design is available in the Nature Portfolio Reporting Summary linked to this article.

## Supplementary information

Supplementary Information

Reporting Summary

Transparent Peer Review file

## Source data

Source Data

## Data Availability

Bulk RNA sequencing data that support the findings of this study have been deposited in NCBI GEO with the accession code GSE301629. The remaining data are available in the Article, Supplementary Information and Source Data file. Source data are provided with this paper.
