# Supplementary material for: HER2∆16 directs luminal cell identity and estrogen receptor signaling in HER2+ breast cancer
Source: Nat Commun. 2026 Jun 15;17:7532. doi: 10.1038/s41467-026-74435-9 (PMC13408682; doi:10.1038/s41467-026-74435-9)
Supplement: Supplementary file 1 — Supplementary Information [file 41467_2026_74435_MOESM1_ESM.pdf]

Supplementary Information

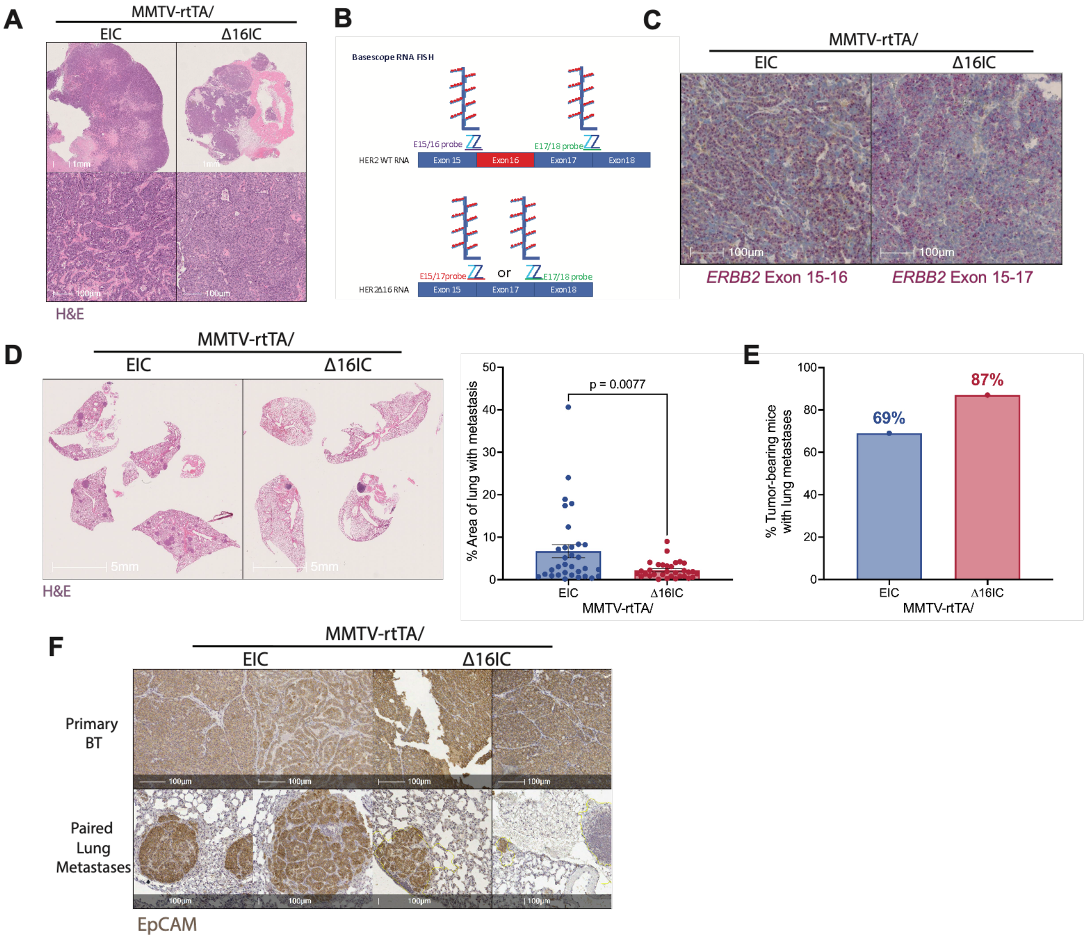

Supplementary Figure 1. EIC and Δ16IC tumors develop spontaneous metastases displaying epithelial marker EpCAM. A) Hematoxylin and eosin staining on representative primary tumors from MMTV-rtTA EIC and Δ16IC tumors (n = 10). B) Schematic depicting unique Z probes for BaseScope assay to detect either the exon 15-16 junction in *HER2* or exon 15-17 junction in *HER2Δ16*, exposed by red substrate. C) Validation of either full-length or splice isoform expression in endpoint EIC and Δ16IC tumors using BaseScope detection of either the full-length or exon 16 splice junctions. D) Hematoxylin and eosin staining on lungs from EIC (n = 30) and Δ16IC (n =32) mice at tumor endpoint. Percentage of lung area with spontaneous metastatic lesions was quantified using the HALO classifier function and shown as mean ± SEM. Statistical analysis by unpaired, two-tailed Student's t-test with Welch's correction. E) Incidence of metastasis to the lung in Δ16IC and EIC mice (n = 32). F) Representative images of immunohistochemical staining for the epithelial marker EpCAM on spontaneous lung metastases in EIC and Δ16IC mice (n = 5).

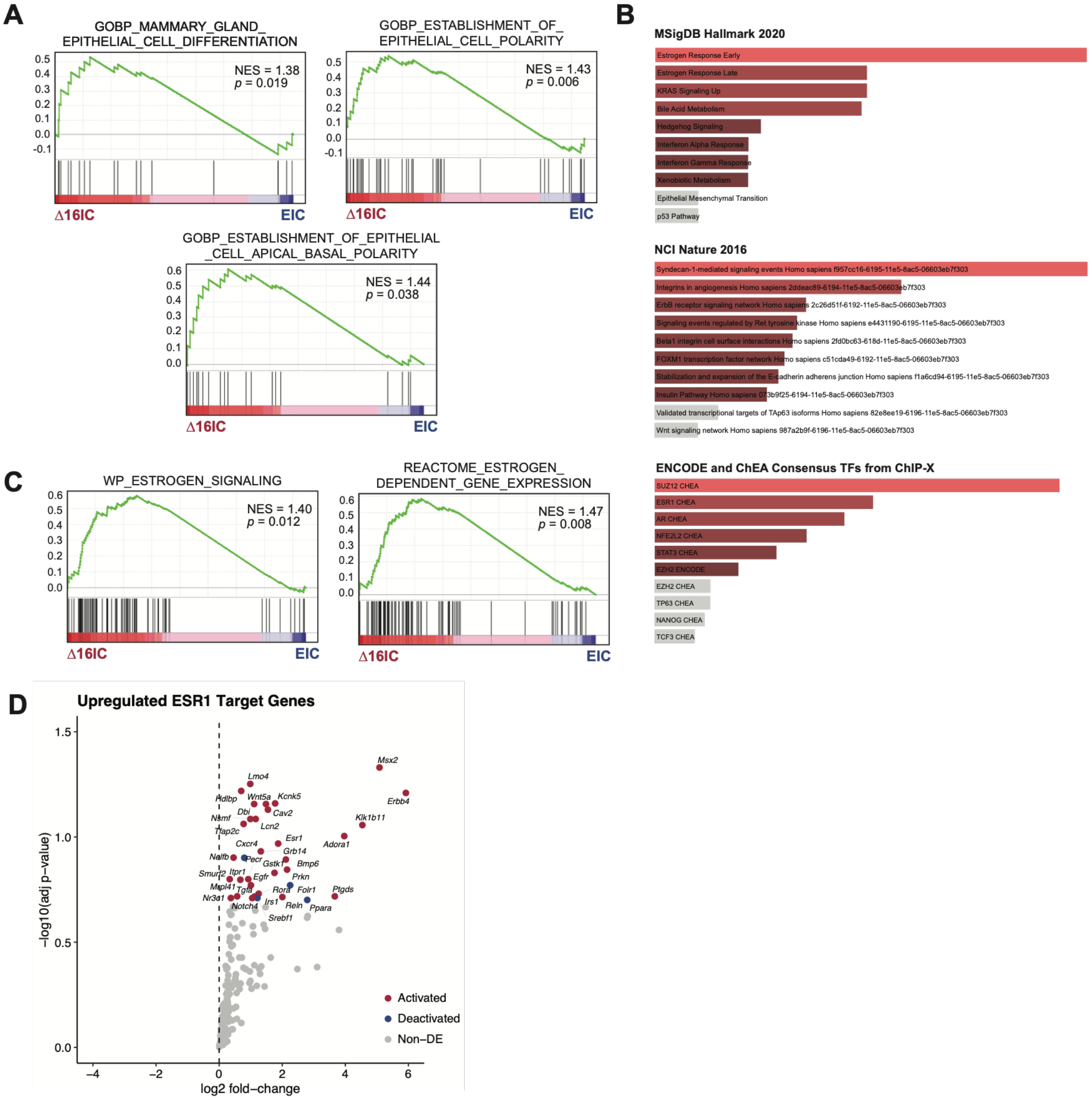

Supplementary Figure 2. Δ16IC tumors have increased expression of luminal gene signatures. A) GSEA for signatures of mammary gland epithelial cell differentiation and cell polarity in Δ16IC versus EIC tumors. B) EnrichR gene set enrichment analysis on enriched pathways in Δ16IC tumors using MSigDB Hallmark, NCI Nature and ENCODE/ChEA datasets. Significant gene signatures ($p<0.05$) denoted in red. C) GSEA for signatures of estrogen receptor signaling and estrogen dependent gene expression in Δ16IC versus EIC tumors. D) Volcano plot depicting differentially expressed Esr1 target genes. Red dots represent DEGs activated within Δ16IC tumors, while blue dots represent deactivated genes. Y-axis denotes -log10 adjusted p-values while x-axis shows log2 fold change.

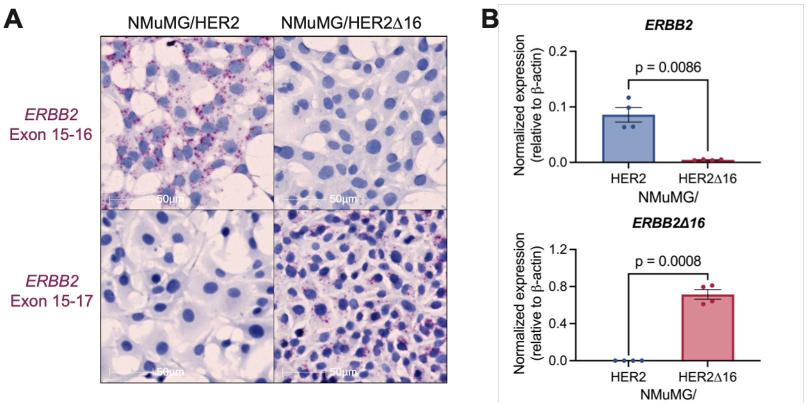

Supplementary Figure 3. Basescope validation of HER2 isoforms in NMuMG murine cell lines. A) Basescope detecting full-length or exon 16 splice isoforms of HER2 in NMuMG cell lines transfected to express human HER2 or HER2Δ16, counterstained with hematoxylin. Unique probes detect either the exon 15-16 junction (HER2) or exon 15-17 junction (HER2Δ16). B) qRT-PCR quantification of *ERBB2* or *ERBB2Δ16* transcript levels in NMuMG cells normalized to *β-actin* (n = 4), shown as mean ± SEM. Statistical analysis by unpaired, two-tailed Student's t-test with Welch's correction.

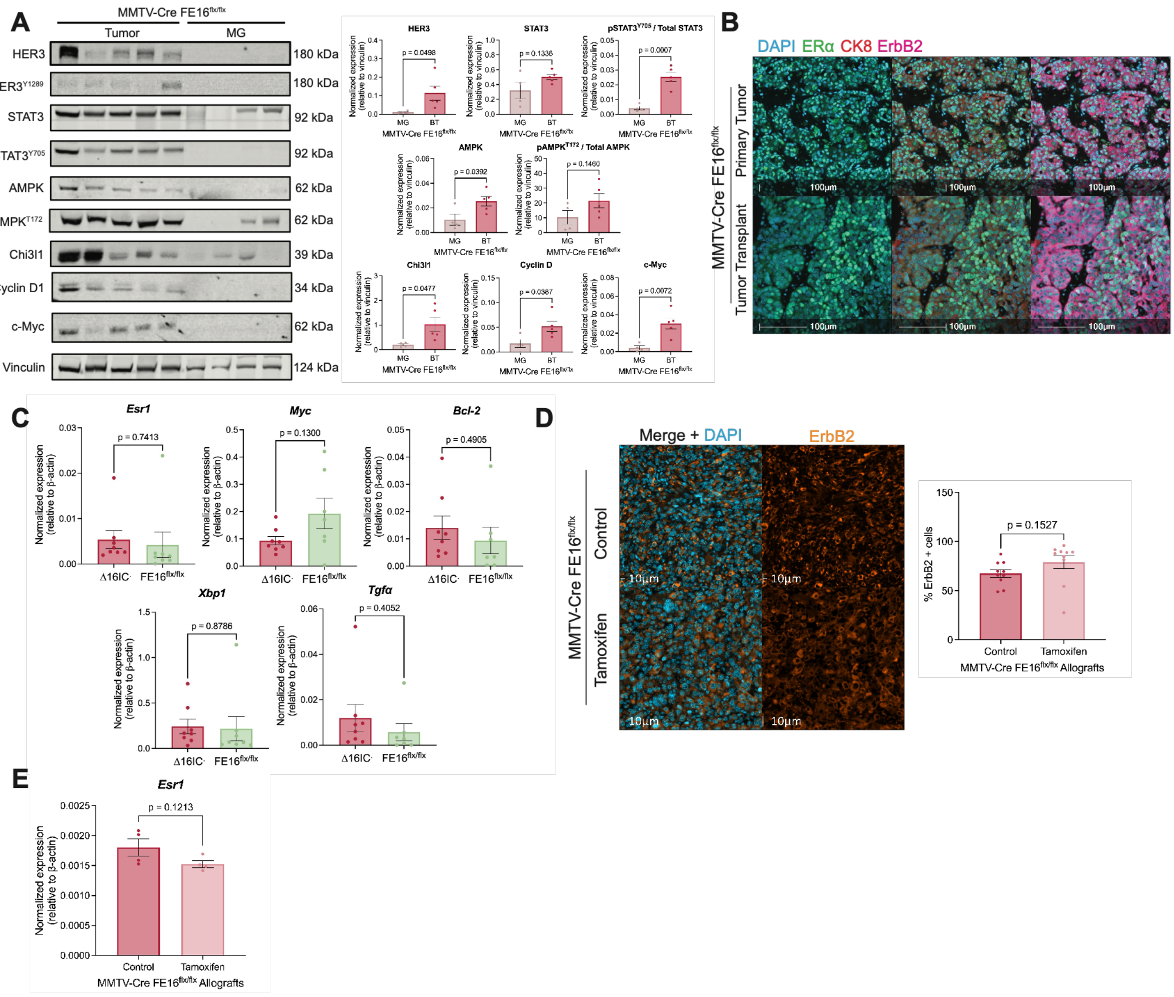

Supplementary Figure 4. HER2/ER receptor status of MMTV-Cre FE16$^{flx/flx}$ primary tumors and tamoxifen treated orthotopic tumor transplants. A) Immunoblots for Her2 signaling pathways, various immune modulators and estrogen receptor targets in primary MMTV-Cre FE16$^{flx/flx}$ tumors (n = 5) and age-matched mammary glands (n = 4), normalized to vinculin. B) Representative images of immunofluorescent staining for ESR1/ERα, CK8 and ErbB2 on primary or orthotopic tumor transplants, counterstained with DAPI. Data shown as mean ± SEM. C) qRT-PCR for ER-target gene expression in Δ16IC and FE16$^{flx/flx}$ end-point tumors normalized to *β-actin* (n = 4 tumors per genotype in duplicate). D) Immunofluorescent staining for ErbB2 on endpoint MMTV-Cre FE16$^{flx/flx}$ tumor transplants treated with either standard rodent or tamoxifen (400mg/kg) diet, counterstained with DAPI. E) qRT-PCR for *Esr1* expression in control and tamoxifen treated MMTV-Cre FE16$^{flx/flx}$ allograft tumors normalized to *β-actin* (n = 4 tumors per treatment group in duplicate). All error bars are expressed as mean values ± SEM. All statistical analysis by unpaired, two-tailed Student's t-test with Welch's correction unless otherwise indicated.

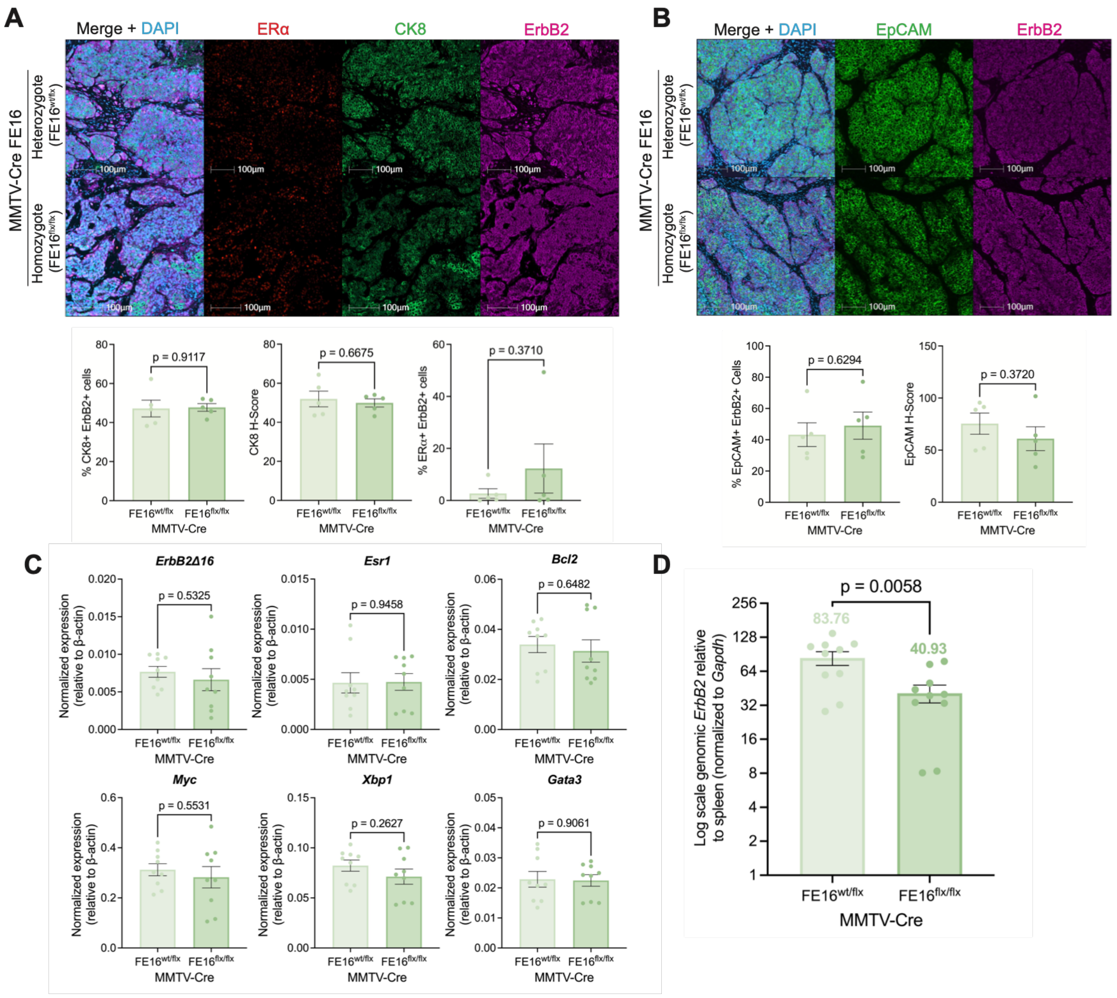

Supplementary Figure 5. Heterozygous deletion of HER2Δ16 is sufficient to drive ER+ mammary tumorigenesis in MMTV-Cre FE16 mice. A) Immunofluorescent staining for epithelial marker cytokeratin-8 (CK8), ErbB2 and Estrogen Receptor α (ER α) (n = 5 tumors per genotype), counterstained with DAPI. Percentage ERα+ or CK8+ tumor cells and average fluorescent intensity (H-score) were quantified via HALO. B) Immunofluorescent staining for the epithelial marker, EpCAM and ErbB2 (n = 5 tumors per genotype), counterstained with DAPI. Percentage EpCAM+ tumor cells and average fluorescent intensity (H-score) were quantified via HALO. C) qRT-PCR for ErbB2Δ16 and ER-target gene expression in $FE16^{wt/flx}$ and $FE16^{flx/flx}$ end-point tumors normalized to β-actin (n = 3 tumors per genotype in triplicate). D) Genomic qPCR quantifying ErbB2 copy number from endpoint tumors in MMTV-Cre $FE16^{wt/flx}$ and $FE16^{flx/flx}$ mice (n = 10). Data was normalized to Gapdh, and copy number was normalized relative to spleen. All error bars are expressed as mean values ± SEM. All statistical analysis by unpaired, two-tailed Student's t-test with Welch's correction unless otherwise indicated.

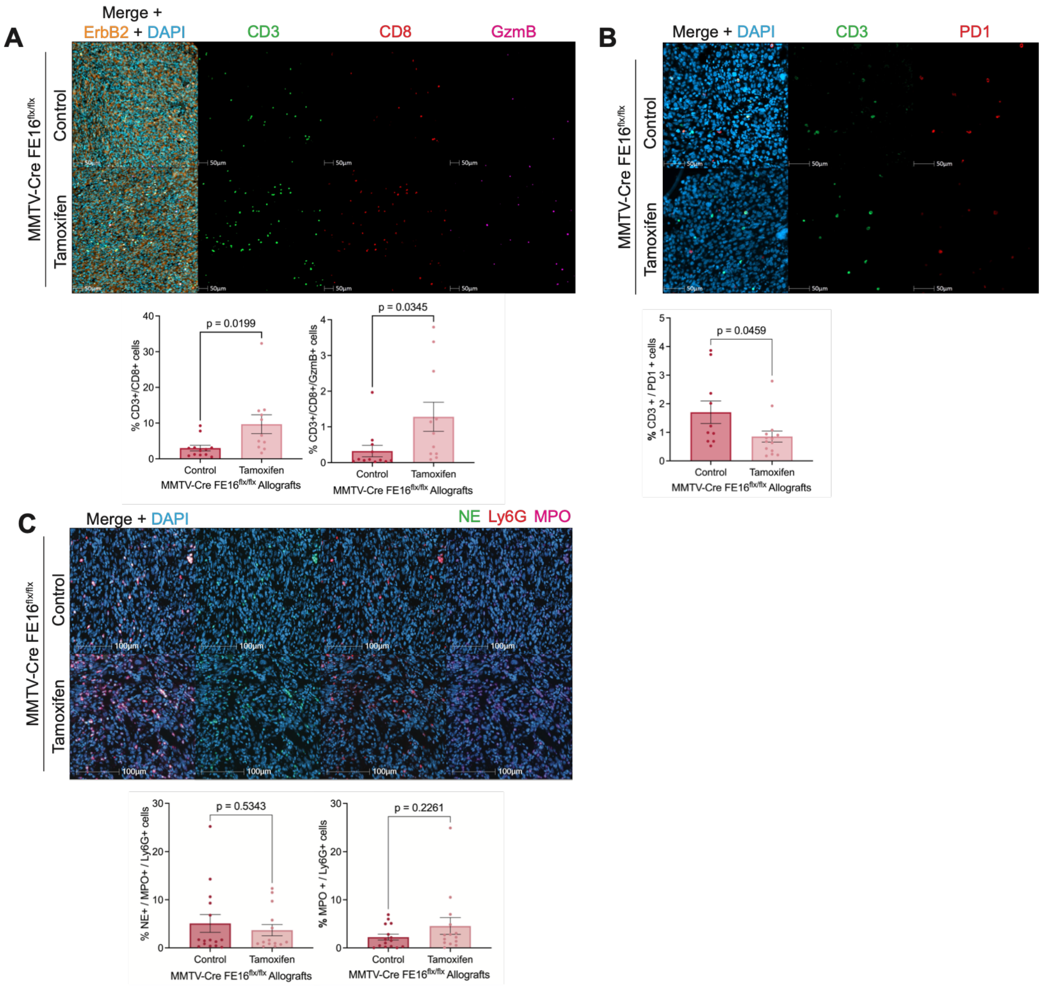

Supplementary Figure 6. Immune characterization of MMTV-Cre FE16$^{flx/flx}$ control and tamoxifen treated orthotopic tumor transplants. A) Immunofluorescent staining for ErbB2, CD3, CD8 and T-cell activation marker, Granzyme B on endpoint MMTV-Cre FE16$^{flx/flx}$ tumor transplants treated with either standard rodent or tamoxifen (400mg/kg) diet, counterstained with DAPI. B) Immunofluorescent staining for CD3, and T-cell exhaustion marker, PD1 on endpoint MMTV-Cre FE16$^{flx/flx}$ tumor transplants treated with either standard rodent or tamoxifen (400mg/kg) diet, counterstained with DAPI.C) Immunofluorescent staining for neutrophil elastase (NE), Ly6G and myeloperoxidase (MPO) on endpoint MMTV-Cre FE16$^{flx/flx}$ tumor transplants treated with either standard rodent or tamoxifen (400mg/kg) diet, counterstained with DAPI. All error bars are expressed as mean values ± SEM. All statistical analysis by unpaired, two-tailed Student's t-test with Welch's correction unless otherwise indicated.

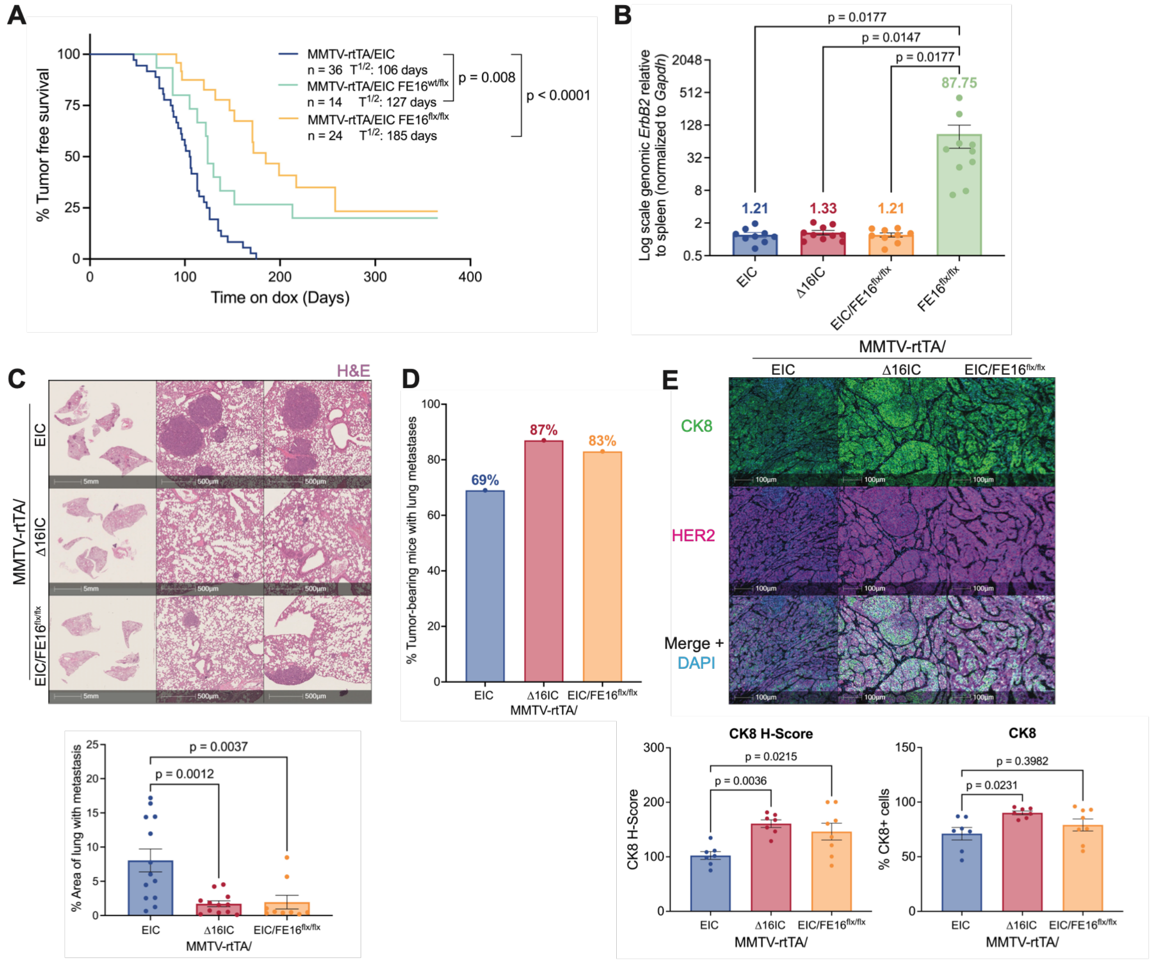

Supplementary Figure 7. EIC FE16$^{flx/flx}$ tumors show elevated CK8 expression and decreased metastatic capacity relative to EIC tumors but lack HER2 amplification. A) Kaplan-Meier curve of mammary tumor onset in EIC (n = 36), EIC FE16$^{wt/flx}$ (n = 15) and EIC FE16$^{flx/flx}$ (n = 24) mice. Analysis by log-rank (Mantel-Cox) test. B) Genomic qPCR quantifying *ErbB2* copy number from endpoint tumors in EIC (n =9) , Δ16IC (n = 10), EIC FE16$^{flx/flx}$ (n= 9) and MMTV-Cre FE16$^{flx/flx}$ mice (n = 10). Data was normalized to *Gapdh*, and copy number was normalized relative to spleen. Statistical analysis by unpaired, two-tailed Student's t-test with Welch's correction. C) Hematoxylin and eosin staining on lungs from EIC (n = 13), Δ16IC (n = 12) and EIC FE16$^{flx/flx}$ (n = 9) mice at tumor endpoint. Percentage of lung area with spontaneous metastatic lesions was quantified using the HALO classifier function. Statistical significance determined by one-way ANOVA with Tukey's post-hoc test. D) Incidence of metastasis to the lung in EIC (n = 32), Δ16IC (n =32) and EIC FE16$^{flx/flx}$ (n = 12) mice. E) Immunofluorescent staining for epithelial marker cytokeratin-8 (CK8) (n = 7 tumors per genotype), counterstained with DAPI. Percentage CK8+ cells and average fluorescent intensity (H-score) were quantified via HALO. Statistical significance determined by one-way ANOVA with Tukey's post-hoc test. All error bars are expressed as mean values ± SEM.

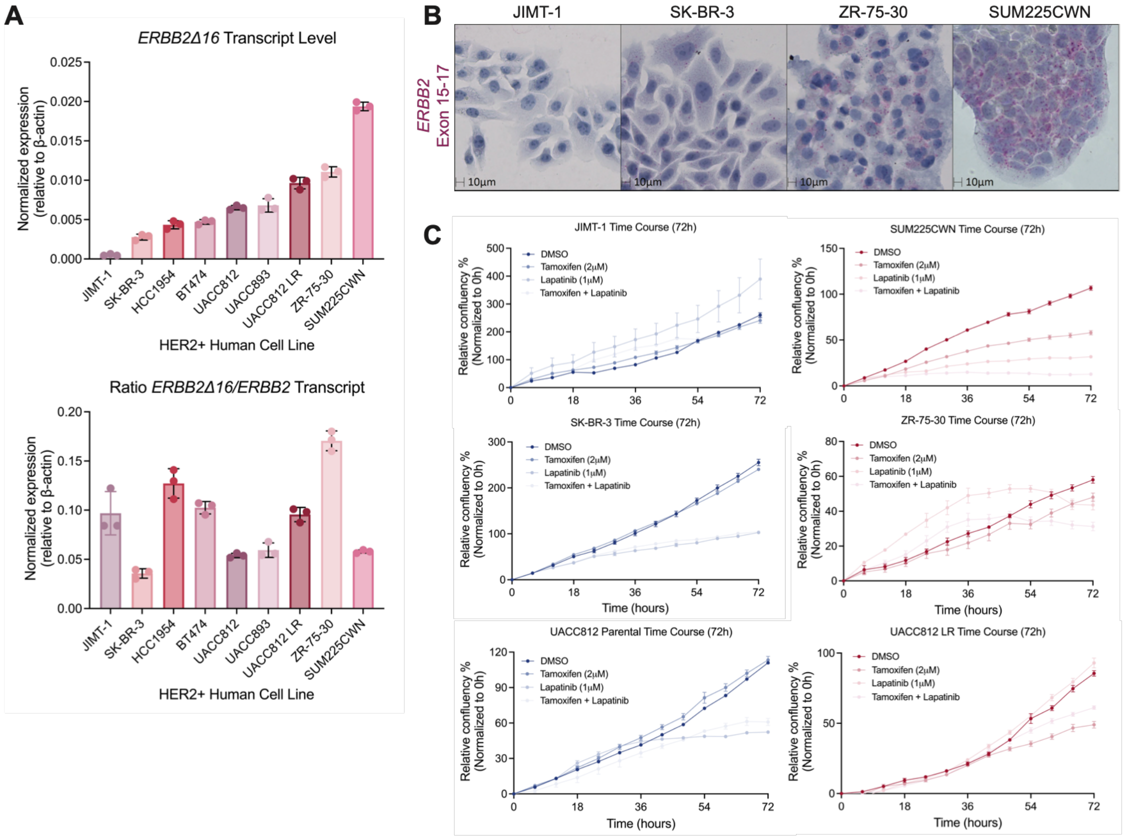

Supplementary Figure 8. Characterization of ERBB2Δ16 transcript levels and tamoxifen/lapatinib sensitivity across human HER2+ breast cancer cell lines. A) qRT-PCR validation of total *ERBB2* and *ERBB2Δ16* transcript levels in human HER2+ cell lines, normalized to β-actin. Each cell line was tested in triplicate. B) Basescope detecting exon 16 splice isoforms of HER2 in human breast cancer cell lines, tested in triplicate, counterstained with hematoxylin. Unique probes detect the exon 15-17 junction (HER2Δ16). C) Individual time course Incucyte proliferation assays on HER2Δ16 low (JIMT-1, SK-BR-3, UACC812 Parental) and high (SUM225CWN, ZR-75-30, UACC812 Lapatinib resistant) cell lines over 72h (data normalized to confluency at t = 0) after treatment with either dimethylsulfoxide (DMSO), tamoxifen (2μM) and/or lapatinib (1μM). Each cell line was tested in sextuplicate. All error bars are expressed as mean values ± SEM.

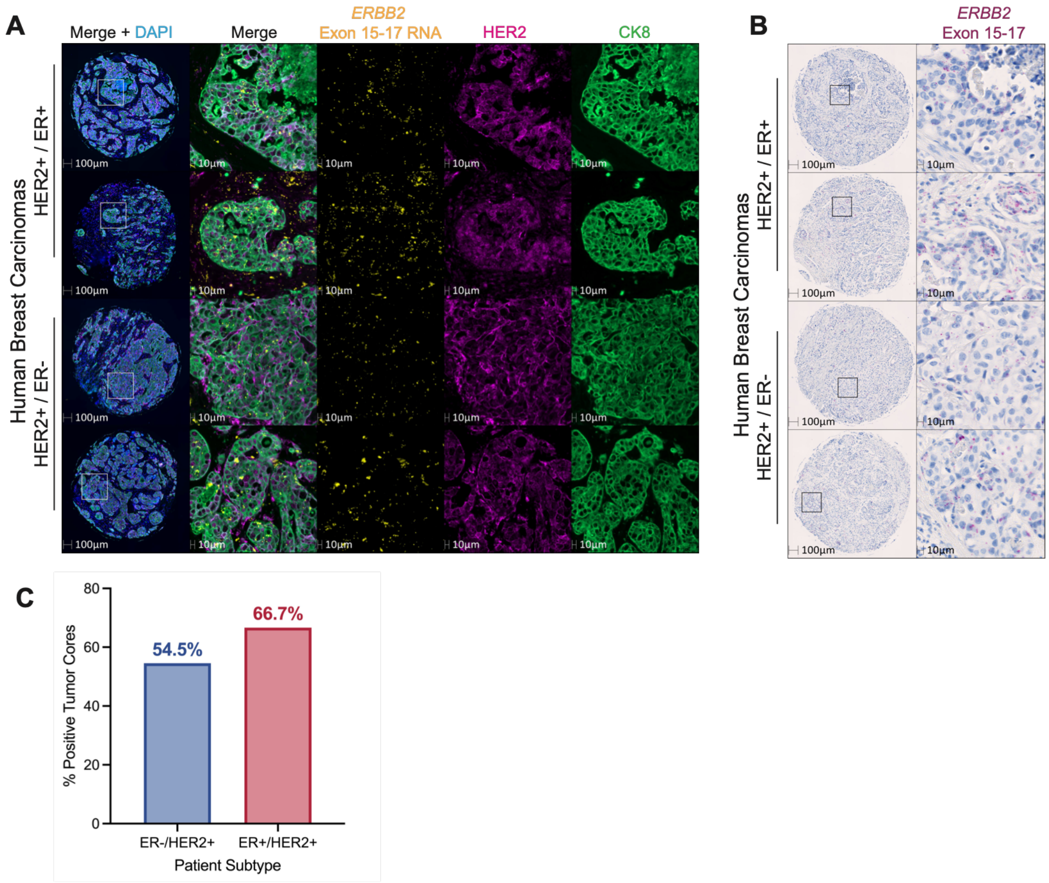

Supplementary Figure 9. HER2Δ16 levels correlate with ER-positivity in human breast tissue samples. A) Representative patient tumor cores from commercial tissue microarray (US Biomax, BR1504b) scored for *ERBB2Δ16* transcript levels along with epithelial markers CK8 and HER2 within the primary tumor as measured by immunofluorescent staining, counterstained with DAPI (n = 75 patients, in duplicate). B) Basescope detecting exon 16 splice isoforms of HER2 in patient tumor cores from commercial tissue microarray (US Biomax, BR1504b) counterstained with hematoxylin (n = 75 patients, in duplicate). Unique probes detect the exon 15-17 junction (HER2Δ16). C) Percentage *ERBB2Δ16* positive tumor cores in patients stratified as HER2+/ER+ (n = 12) or HER2+/ER- (n = 33) by immunohistochemistry.

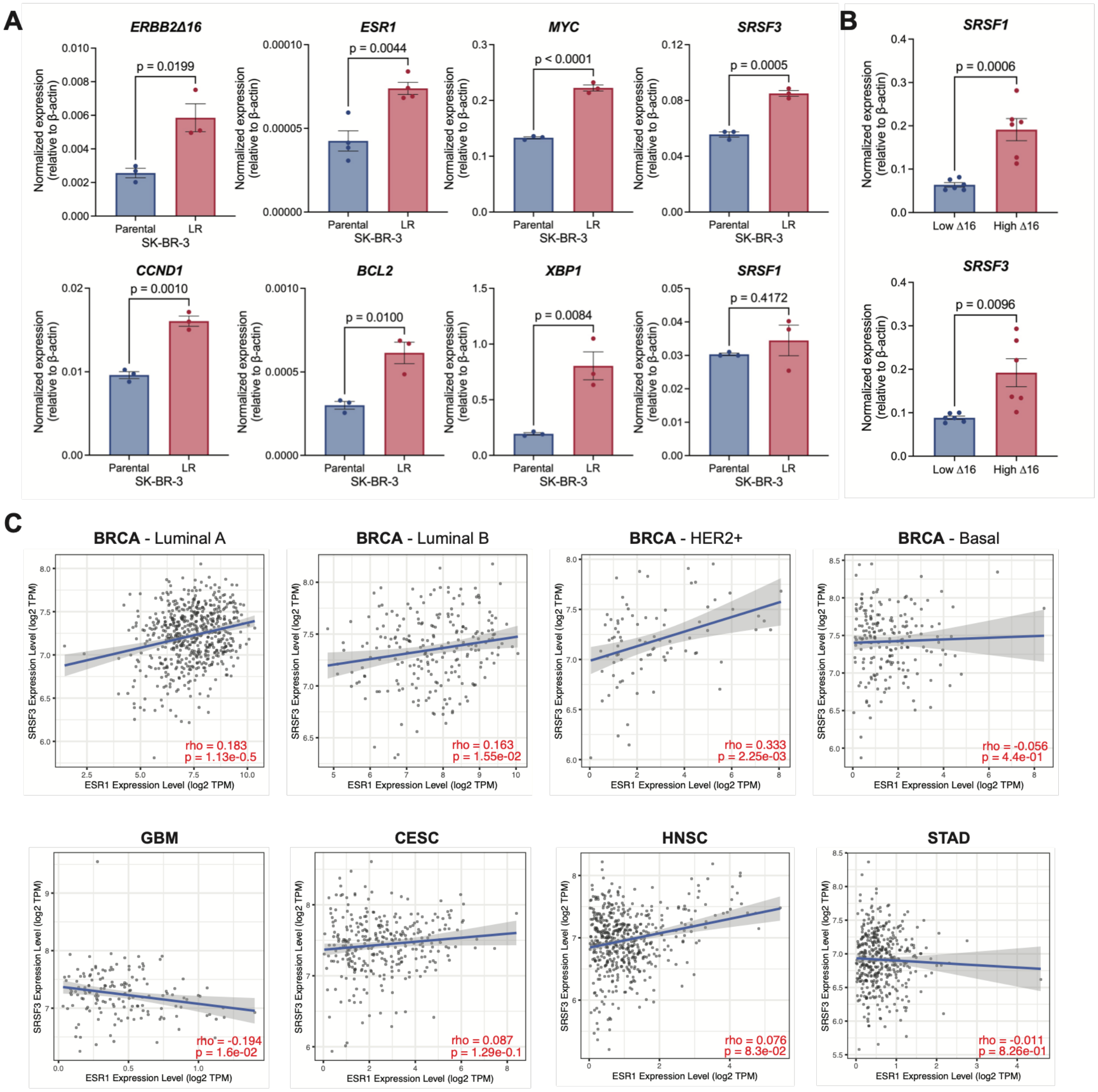

Supplementary Figure 10. SRSF3 expression correlates with ER pathway activation and HER2Δ16 levels in human breast cancer. A) qRT-PCR for ER-target gene expression, *SRSF1/3* and *ERBB2Δ16* transcript levels in HER2+ parental and lapatinib-resistant (LR) SK-BR-3 cells, normalized to β-actin (n = 3), shown as mean ± SEM. B) qRT-PCR for *SRSF3* and *SRSF1* transcript levels in HER2Δ16 low or high cell lines (2 biological groups per cohort, tested in triplicate) normalized to β-actin, shown as mean ± SEM. C) Association between SRSF3 and ESR1 levels across breast cancer subtypes and other solid malignancies (glioblastoma, cervical squamous cell carcinoma, head and neck squamous cell carcinoma, stomach adenocarcinoma) within the TCGA database using TIMER2.0.

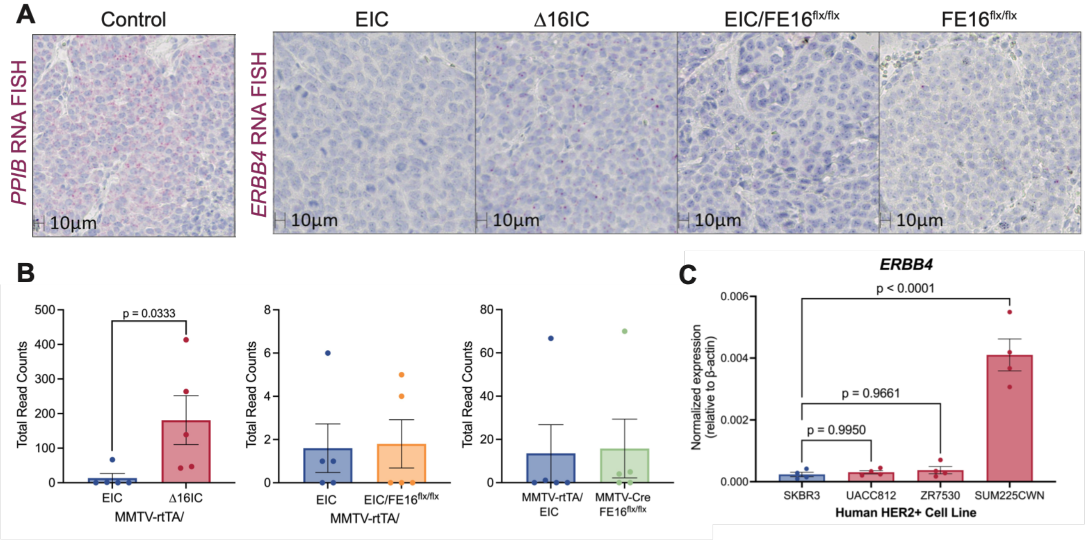

Supplementary Figure 11. ERBB4 expression is not uniformly upregulated across murine or human models of HER2Δ16 positive breast cancer. A) RNA ISH detecting *ERBB4* or control *PPIB* RNA expression across endpoint tumors from transgenic models of HER2+ breast cancer, counterstained with hematoxylin. B) Total read counts from RNA-sequencing of endpoint tumors from EIC, Δ16IC, EIC/FE16$^{flx/flx}$ or FE16$^{flx/flx}$ transgenic models (n = 5 per genotype). Differential expression analysis performed, and adjusted using Benjamini and Hochberg's method C) qRT-PCR quantification of *ERBB4* transcript levels across human HER2+ cell lines normalized to *β-actin,* shown as mean ± SEM. Each cell line was tested in quadruplicate. Statistical significance determined by one-way ANOVA with Tukey's post-hoc test.

Supplementary Tables

Supplementary Table S1: Genotyping Primers

Gene	Sequence	Species	Source
MTB Forward Primer	5'-ACCGTACTCGTCAATTCCAAGGG-3'	Mouse	Gunther *et al*, 2002
MTB Reverse Primer	5'-TGCCGCCATTATTACGACAAGC-3'	Mouse	Gunther *et al*, 2002
Cre Forward Primer	5'- TGCTCTGTCCGTTTGCCG-3'	Mouse	Attalla, *et al*, 2023
Cre Reverse Primer	5'-ACTGTGTCCAGACCAGGC-3'	Mouse	Attalla, *et al*, 2023
Her2 Forward Primer 1	5'-GTGACCTGTTTTGGACCGGA-3'	Human	Attalla, *et al*, 2023
Her2 Forward Primer 2	5'-TGTGTGGACCTGGATGACAAGG-3'	Human	Attalla, *et al*, 2023
Her2 Reverse Primer	5'-TCTCCGCATCGTGTACTTCC-3'	Human	Attalla, *et al*, 2023
FE16 Forward Primer	5'-GATCTGCTCAGGATGCTTTGG-3'	Mouse	Attalla, *et al*, 2023
FE16 Forward Primer	5'-AAGCTGCCACTGCACTCTGC-3'	Mouse	Attalla, *et al*, 2023

Supplementary Table S2: Quantitative RT-PCR primer sequences

Gene	Sequence	Species
Esr1 Forward Primer	5’- CCTCCCGCCTTCTACAGGT-3’	Mouse
Esr1 Reverse Primer	5’- CAGACGGCACAGTAGCGAG-3’	Mouse
ESR1 Forward Primer	5’- GGGAAGTATGGCTATGGAATCTG-3’	Human
ESR1 Reverse Primer	5’- TGGCTGGACACATATAGTCGTT-3’	Human
Myc Forward Primer	5’- ATGCCCCTCAACGTGAACTTC-3’	Mouse
Myc Reverse Primer	5’- GTCGCAGATGAAATAGGGCTG-3’	Mouse
MYC Forward Primer	5’- GGCTCCTGGCAAAAGGTCA-3’	Human
MYC Reverse Primer	5’-CTGCGTAGTTGTGCTGATGT-3’	Human
Tgfα Forward Primer	5’- GGAACCTGCCGGTTTTTGG-3’	Mouse
Tgfα Reverse Primer	5’- CACAGCGAACACCCACGT-3’	Mouse
Bcl2 Forward Primer	5’-ATGCCTTTGTGGAACTATATGGC-3’	Mouse
Bcl2 Reverse Primer	5’-GGTATGCACCCAGAGTGATGC-3’	Mouse
BCL2 Forward Primer	5’-GGTGGGGTCATGTGTGTGG-3’	Human
BCL2 Reverse Primer	5’-CGGTTCAGGTACTCAGTCATCC-3’	Human
Gata3 Forward Primer	5’-CTCGGCCATTCGTACATGGAA-3’	Mouse
Gata3 Reverse Primer	5’-GGATACCTCTGCACCGTAGC-3’	Mouse
GATA3 Forward Primer	5’-GCCCCTCATTAAGCCCAAG-3’	Human
GATA3 Reverse Primer	5’-TTGTGGTGGTCTGACAGTTCG-3’	Human
Rprm1 Forward Primer	5’-GTGGTGCAGATCGCAGTCAT-3’	Mouse
Rprm1 Reverse Primer	5’-CGGTCCTTCACTAGGAAGTTGA-3’	Mouse
Xbp1 Forward Primer	5’-AGCTTTTACGGGAGAAAACTCAC-3’	Mouse
Xbp1 Reverse Primer	5’-CCTCTGGAACCTCGTCAGGA-3’	Mouse
XBP1 Forward Primer	5’-CCCTCCAGAACATCTCCCCAT-3’	Human
XBP1 Reverse Primer	5’-ACATGACTGGGTCCAAGTTGT-3’	Human
Ncoa3 Forward Primer	5’-AGTGGACTAGGCGAAAGCTCT-3’	Mouse
Ncoa3 Reverse Primer	5’-GTTGTCGATGTCGCTGAGATTT-3’	Mouse
NCOA3 Forward Primer	5’-ACATAAACGCCAGTCCTGAAAT-3’	Human
NCOA3 Reverse Primer	5’-CCTTCCTCCATCATAGCTCGT-3’	Human
Her2 Forward Primer	5’-TCTGGAAGTACCCGGATGAG-3’	Mouse
Her2 Reverse Primer	5’-GGATTCCAATGACCACCACT-3’	Mouse
HER2 Forward Primer	5’-GTGGACCTGGATGACAAGGG-3’	Human
HER2 Reverse Primer	5’-TGCTGCCGTCGCTTGATGAG-3’	Human
HER2Δ16 Forward Primer	5’-CCATCAACTGCACCCACTCCC-3’	Human
HER2Δ16 Reverse Primer	5’-GCCTACGCATGGTATACTTCCGG-3’	Human
HER4 Forward Primer	5’ -GCAGATGCTACGGACCTTACG-3’	Human
HER4 Reverse Primer	5’- GACACTGAGTAACACATGCTCC-3’	Human
Grb7 Forward Primer	5’-GCCTCTACTACTCTACCAAGGG-3’	Mouse
Grb7 Reverse Primer	5’-CAGGTCCGACTCTGCTCAT-3’	Mouse
Stard3 Forward Primer	5’-TCCCTTCTCTGGATCATCGAG-3’	Mouse

Stard3 Reverse Primer	5'-TGGAGACGGAGCACAGCATA-3'	Mouse
PGR Forward Primer	5'-ACCCGCCCTATCTCAACTACC-3'	Human
PGR Reverse Primer	5'-AGGACACCATAATGACAGCCT-3'	Human
CCND1 Forward Primer	5'-GCTGCGAAGTGGAAACCATC-3'	Human
CCND1 Reverse Primer	5'-CCTCCTTCTGCACACATTTGAA-3'	Human
FOXA1 Forward Primer	5'-GCAATACTCGCCTTACGGCT-3'	Human
FOXA1 Reverse Primer	5'-TACACACCTTGGTAGTACGCC-3'	Human
SRSF1 Forward Primer	5'-CCGCAGGGAACAACGATTG-3'	Human
SRSF1 Reverse Primer	5'-GCCGTATTTGTAGAACACGTCCT-3'	Human
SRSF3 Forward Primer	5'-TGGCAACAAGACGGAATTGGA-3'	Human
SRSF3 Reverse Primer	5'-CAAAGCCGGGTGGGTTTCTA-3'	Human
β-Actin Forward Primer	5'- TCCATCATGAAGTGTGACGT-3'	Mouse
β-Actin Reverse Primer	5'- GAGCAATGATCTTGATCTTCAT-3'	Mouse
β-ACTIN Forward Primer	5'-AGAGCTACGAGCTGCCTGAC-3'	Human
β-ACTIN Reverse Primer	5'-AGCACTGTGTTGGCGTACAG-3'	Human
Genomic *Her2* Forward Primer	5'-GAGGGTCCATCCAGTTGCTA-3'	Mouse
Genomic *Her2* Reverse Primer	5'-AGGCCTTCTTGGAACCTTGT-3'	Mouse
Genomic *Gapdh* Forward Primer	5'-CTCTTCCACCTTCGATGCCG-3'	Mouse
Genomic *Gapdh* Reverse Primer	5'-AGGTTTCCCATCCCCACATACC-3'	Mouse

All primer sequences were generated utilizing the PrimerBank database except genomic sequences which were published by Attalla *et al*, 2023.

Supplementary Table S3: Primary and Secondary Antibodies

**Antibody**	**Source**	**Catalog #**	**Dilution**
β-Actin	Millipore	A5441	IB: 1/2000
pan-AKT	Cell Signaling	2920	IB: 1/1000
pAKT (S473)	Cell Signaling	9018	IB: 1/500
pAKT (T304)	Cell Signaling	4056	IB: 1/500
AMPK	Cell Signaling	5831	IB: 1/500
pAMPK (T172)	Cell Signaling	2535	IB: 1/500
CD206 / MRC1	Cell Signaling	24595	IF: 1/400
CD3-epsilon	Abcam	16669	IF: 1/200
CD4	Cell Signaling	25229	IF: 1/200
CD8*α*	Cell Signaling	98941	IF:1/200
Chi3l1	Invitrogen	PA5-37357	IB: 1/500
Cyclin D1	Cell Signaling	2978	IB: 1/500
Cytokeratin 5	Biolegend	905504	IF: 1/100
Cytokeratin 8	Biolegend	904804	IF: 1/100
Cytokeratin 14	Ventana	760-4805	IF: 1/10
E-Cadherin	BD Biosciences	610182	IF: 1/200

			IB: 1/1000
Erα*	Santa Cruz	sc-542	IF: 1/200
pER (S118)	Invitrogen	PA5-99347	IB: 1/500
EpCAM	Cell Signaling	93790	IF: 1/400<br>IB: 1/1000
F4/80	Cell Signaling	70076	IF: 1/400
GATA3	Santa Cruz<br>Cell Signaling	sc-268<br>5852	IF: 1/100<br>IB: 1/1000
Granzyme B	Cell Signaling	44153	IF: 1/100
HER2	DAKO<br>Cell Signaling	A0485<br>2165	IF: 1/400<br>IB: 1/500
pHER2 (Y1221/1222)	Cell Signaling	2243	IB: 1/500
HER3	Cell Signaling	12708	IB: 1/500
pHER3 (Y1289)	Cell Signaling	4791	IB: 1/500
Ki67	Cell Signaling	12202	IF: 1/400
Ly6G	Cell Signaling	87048	IF: 1/100
MAPK	Cell Signaling	9102	IB: 1/1000
pMAPK (T202/Y204)	Cell Signaling	4370	IF: 1/400<br>IB: 1/500
c-Myc	Cell Signaling	18583	IB: 1/500
Myeloperoxidase	Abcam	208670	IF: 1/500
Neutrophil Elastase	Cell Signaling	90120	IF: 1/200
PD1	Cell Signaling	84651	IF: 1/200
pSTAT1 (Y701)	Cell Signaling	9167	IF: 1/200
STAT3	Cell Signaling	9139	IB: 1/1000
pSTAT3 (Y705)	Cell Signaling	9145	IB: 1/500
Vinculin	Millipore	MAB3574	IB: 1/2000
IRDye 800CW Donkey anti-Rabbit	Li-COR Biosciences	925-32213	IB: 1/10000
IRDye 680RD Donkey anti-Mouse	Li-COR Biosciences	926-68073	IB: 1/10000

**Discontinued*
