# Supplementary material for: HER2∆16 directs luminal cell identity and estrogen receptor signaling in HER2+ breast cancer
Source: Nat Commun. 2026 Jun 15;17:7532. doi: 10.1038/s41467-026-74435-9 (PMC13408682; doi:10.1038/s41467-026-74435-9)
Supplement: Supplementary file 2 — Reporting Summary [file 41467_2026_74435_MOESM2_ESM.pdf]

nature portfolio

Corresponding author(s): William J. Muller

Last updated by author(s): June 3, 2026

Reporting Summary

Nature Portfolio wishes to improve the reproducibility of the work that we publish. This form provides structure for consistency and transparency in reporting. For further information on Nature Portfolio policies, see our Editorial Policies and the Editorial Policy Checklist.

Statistics

For all statistical analyses, confirm that the following items are present in the figure legend, table legend, main text, or Methods section.

n/a	Confirmed	
☐	☒	The exact sample size (*n*) for each experimental group/condition, given as a discrete number and unit of measurement
☐	☒	A statement on whether measurements were taken from distinct samples or whether the same sample was measured repeatedly
☐	☒	The statistical test(s) used AND whether they are one- or two-sided<br>*Only common tests should be described solely by name; describe more complex techniques in the Methods section.*
☒	☐	A description of all covariates tested
☐	☒	A description of any assumptions or corrections, such as tests of normality and adjustment for multiple comparisons
☐	☒	A full description of the statistical parameters including central tendency (e.g. means) or other basic estimates (e.g. regression coefficient) AND variation (e.g. standard deviation) or associated estimates of uncertainty (e.g. confidence intervals)
☐	☒	For null hypothesis testing, the test statistic (e.g. *F*, *t*, *r*) with confidence intervals, effect sizes, degrees of freedom and *P* value noted<br>*Give P values as exact values whenever suitable.*
☒	☐	For Bayesian analysis, information on the choice of priors and Markov chain Monte Carlo settings
☒	☐	For hierarchical and complex designs, identification of the appropriate level for tests and full reporting of outcomes
☐	☒	Estimates of effect sizes (e.g. Cohen's *d*, Pearson's *r*), indicating how they were calculated

Our web collection on statistics for biologists contains articles on many of the points above.

Software and code

Policy information about availability of computer code

Data collection

Tumor, mammary gland wholemounts and lung metastasis: All solid organ tissues stained with H&E were scanned using Scanscope XT Digital Slide Scanner (Aperio Technologies) and analyzed using HALO 2.0 software (Indica Lab).

Fluorescent IHC and, RNA Scope: Imaging was performed using a Zeiss AxioScan Z1 digital slide scanner, and analyzed using HALO software (Indica Labs, v3.5.3577) using the algorithms “HighPlex FL v4.0.4” for IHF or “Multiplex IHC v2.3.4”. The entire tissue section was analyzed for all staining experiments. Staining intensity was quantitatively defined on HALO using an algorithm recognizing the individual fluorescent intensities (scored at 0, +1, +2 and +3) of single cells to determine a relative H-score representing the degree of staining intensity across the entire section. RNAscope in situ hybridization was performed on paraffin-embedded tumor sections from endpoint lesions using RNAscope 2.5 HD Assay-RED Kit (ACD, 322360) as per the manufacturer’s protocol. Probes against murine Esr1 (ACD, 478201) and the control, Ppib (ACD, 313911) were used, and the protocol was followed with fluorescent immunohistochemistry. Imaging was performed using a Zeiss AxioScan Z1 digital slide scanner, and analyzed using HALO software (Indica Labs, v3.5.3577).

RNA extraction and RT-qPCR: Flash-frozen pieces of tumors were crushed in liquid nitrogen. Total RNA was isolated using FavorPrep™ Tissue Total RNA Mini Kit (Cat Number FATRK 001) according to manufacturer’s protocol. RNA quantity was determined using NanoDrop Spectrophotometer ND-1000 (NanoDrop Technologies, Inc.). cDNA was synthesized by reverse transcription using the TranScript all-in-one first strand cDNA synthesis kit (Transgen Biotech). Real-time qPCR was performed using LightCycler 480 SYBR Green I Master Reagents (Roche). Data were normalized to B-actin to generate the relative transcript levels. Primer sequences are provided in Supplementary Table S2.

Genomic PCR: Genomic DNA was extracted from either endpoint tumors, mammary gland or spleens from transgenic mice using the Monarch Spin gDNA Extraction Kit (New England Biolabs, T3010S) as per the manufacturer’s protocol. Reaction mixture of diluted DNA (6.25ng/µL), Sybr Green 1 MasterMix (Roche, 04887352001), and primers for murine ErbB2 or Gapdh were run in duplicate per biological replicate in the

Lightcycler 480 (Roche). Gapdh was used to normalize all the samples and the ΔCt of the spleen was used to normalize the copy number of the mammary gland and tumor samples.

Immunoblot: Images were acquired using Li-Cor Odyssey Scanner. Band intensity quantification was done using Image Studio Lite software (Li-Cor). Primary and secondary antibodies are detailed in Supplementary Table S3. Target proteins were normalized to loading controls on the same membrane.

BaseScope: The BaseScope assay for human wild-type Her2 (ACD, 70112) and human Her2Δ16 (ACD, 701111) was carried out as per the manufacturer's protocol using the BaseScope Detection Kit v2 (ACD, 323910). Signal was detected using Fast-Red alkaline phosphatase substrate (ACD, 323900) and slides were scanned using the Zeiss AxioScan Z1 digital slide scanner.

Incucyte Cell Proliferation Assays: 1000-10000 cells/well were seeded in sextuplicate in 96-well optical-bottom plates (Nunc, 167008), drugs or vehicle controls were added after 24h, and the IncuCyte S3 system (ESSEN BioSciences, Ann Arbor, MI, USA) was used for live cell imaging. Cells were imaged at 10x magnification every 6hours over a period of 48-72 hours with 4 images taken per well, per timepoint. Confluence was determined using the IncuCyte S3 Analysis software (ESSEN BioSciences, v2019A), and percentage confluence relative to the initial (0 h) timepoint was calculated.

Data analysis

Statistical Analysis was performed on Prism 9.0 (Broad Institute, v4.i0) and compiled on Adobe Illustrator (v25.2.3). HALO Software Image Analysis (Indica Labs, v3.5.3577), Image Studio Lite (v5.2.1, Li-COR Biosciences), Microsoft Excel (v16.66.1, Microsoft), and IncuCyte S3 system (ESSEN BioSciences) were performed for relevant experiments. Within HALO Software Image Analysis, the algorithm "HighPlex FL v4.0.4" was used for quantification of immunofluorescent staining, and "Multiplex IHC v2.3.4" for immunohistochemistry. Gene set enrichment analysis (GSEA) was performed using individual gene counts from each genotype and mapped against the MSigDB mouse gene set collection (Broad Institute, GSEA v 4.3.3). Differentially expressed genes were then analyzed by EnrichR, using genes with a minimum 2-fold change difference between genotypes and a minimum of 50 reads. Transcription factor activity scores for each sample were obtained using DecoupleR and a list of activated or deactivated Esr1 target genes were generated33. BioRender was used to created schematic diagrams and figures (https://www.biorender.com/). More details are provided in the manuscript and figures.

For manuscripts utilizing custom algorithms or software that are central to the research but not yet described in published literature, software must be made available to editors and reviewers. We strongly encourage code deposition in a community repository (e.g. GitHub). See the Nature Portfolio guidelines for submitting code & software for further information.

Data

Policy information about availability of data

All manuscripts must include a data availability statement. This statement should provide the following information, where applicable:

- Accession codes, unique identifiers, or web links for publicly available datasets
- A description of any restrictions on data availability
- For clinical datasets or third party data, please ensure that the statement adheres to our policy

Bulk RNA sequencing data that support the findings of this study have been deposited in NCBI GEO with the accession code GSE301629 (https://www.ncbi.nlm.nih.gov/geo/query/acc.cgi?acc=GSE301629). The remaining data are available in the Article, Supplementary Information and Source Data file. Source Data are provided with this paper.

Research involving human participants, their data, or biological material

Policy information about studies with human participants or human data. See also policy information about sex, gender (identity/presentation), and sexual orientation and race, ethnicity and racism.

Reporting on sex and gender	Our research takes into consideration that breast cancer predominately affects women. To reflect the patient demographic of breast cancer, we predominately use female mouse models to accurately depict the epidemiology of the disease, as woman are at a much higher risk of developing breast cancer. However, we also recognize that while rare, 1% of breast cancers are made up of men.
Reporting on race, ethnicity, or other socially relevant groupings	This study does not take into account race, ethnicity, or other socially relevant groupings.
Population characteristics	This study does not take into account population characteristics.
Recruitment	This study does not involve recruitment.
Ethics oversight	n/a

Note that full information on the approval of the study protocol must also be provided in the manuscript.

Field-specific reporting

Please select the one below that is the best fit for your research. If you are not sure, read the appropriate sections before making your selection.

☒ Life sciences ☐ Behavioural & social sciences ☐ Ecological, evolutionary & environmental sciences

Life sciences study design

All studies must disclose on these points even when the disclosure is negative.

Sample size	Sample sizes are indicated in each figure and figure legend. No power analysis or statistical method were used to calculate the sample size, they were determinated based on our previous experience. All experiments utilizing mouse specimens included enough replicates to accommodate for biological heterogeneity between samples while limiting unnecessary over-treatment and use of live models. This method of sample size generation was published previously (Nandi et al, 2024: https://www.nature.com/articles/s41467-024-50998-3). Data from all experiments were analyzed and p-values from statistical tests used to assess statistical significant and appropriateness of sample sizes.
Data exclusions	No data was excluded from the analysis and the study.
Replication	For all in vivo experiments, n is indicated for each in the manuscript for independent biological replicates.Multiple samples were obtained per experimental mouse at their respective experimental endpoints experiments and each data point indicates one replicate on graphs. All attempts are replication were successful. In vivo experiments were not replicated for logistic and ethical reasons. Replicates are shown in the figure and outlined in the figure legends.
Randomization	For all in vivo treatment experiments, experimental female mice were randomly assigned to a treatment arm.
Blinding	For all in vivo therapeutic treatment experiments, drug administration and tumor measurements were performed by different individuals, the latter blinded with respect to the treatment arms.

Reporting for specific materials, systems and methods

We require information from authors about some types of materials, experimental systems and methods used in many studies. Here, indicate whether each material, system or method listed is relevant to your study. If you are not sure if a list item applies to your research, read the appropriate section before selecting a response.

Materials & experimental systems

n/a	Involved in the study	
☐	☒	Antibodies
☐	☒	Eukaryotic cell lines
☒	☐	Palaeontology and archaeology
☐	☒	Animals and other organisms
☒	☐	Clinical data
☒	☐	Dual use research of concern
☒	☐	Plants

Methods

n/a	Involved in the study	
☒	☐	ChIP-seq
☒	☐	Flow cytometry
☒	☐	MRI-based neuroimaging

Antibodies

Antibodies used	Fluorescent IHC: The following antibodies were used for fluorescent IHC on mouse tissue: CD206 (Cell Signaling Technology (CST), 24595, 1:400), CD3-epsilon (Abcam, 16669, 1:200), CD4 (CST, 25229, 1:200), CD8α (CST, 98941, 1:200), Cytokeratin 5 (Biolegend, 905504, 1:100), Cytokeratin 8 (Biolegend, 904804, 1:100), Cytokeratin 14 (Ventana, 760-4805, 1:10), E-Cadherin (BD Biosciences, 610182, 1:200), Erα (discontinued, Santa Cruz, sc-542, 1:200), EpCAM (CST, 93790, 1:400), F4/80 (CST, 70076, 1:400), GATA3 (Santa Cruz, sc-268, 1:100), Granzyme B (CST, 44153, 1:100) HER2 (DAKO, A0485, 1:400), Ki67 (CST, 12202, 1:400), Ly6G (CST, 87048, 1:100), pMAPK/T202/Y204 (CST, 4370, 1:400), Myeloperoxidase (Abcam, 208670, 1:500), Neutrophil Elastase (CST, 90120, 1:200), pSTAT1/Y701 (CST, 9167, 1:200), PD1 (CST, 84651, 1:200).<br><br>Immunoblot: The following antibodies were used for immunoblots: β-Actin (Millipore, A5441, 1:2000), pan-AKT (CST, 2920, 1:1000), pAKT/S473 (CST, 9018, 1:500), pAKT/T304 (CST, 4056, 1:500), AMPK (CST, 5831, 1:500), pAMPK/T172 (CST, 2535, 1:500), Chi3l1 (Invitrogen, PA5-37357, 1:500), Cyclin D1 (CST, 2978, 1:500), E-cadherin (BD Biosciences, 610182, 1:1000), pER/S118 (Invitrogen, PA5-99347, 1:500), EpCAM (CST, 93790, 1:1000), GATA3 (CST, 5852, 1:1000), HER2 (CST, 2165, 1:500), pHER2/Y1221/Y1222 (CST, 2243, 1:500), HER3 (CST, 12708, 1:500), pHER3/Y1289 (CST, 4791, 1:500), MAPK (CST, 9102, 1:1000), pMAPK/T202/Y204 (CST, 4370, 1:500), c-Myc (CST, 18583, 1:500), STAT3 (CST, 9139, 1:1000), pSTAT3/Y705 (CST, 9145, 1:500), Vinculin (Millipore, MAB3574, 1:2000), IRDye 800CW Donkey anti-Rabbit (Li-COR Biosciences, 925-32213, 1:10000), IRDye 680RD Donkey anti-Mouse (Li-COR Biosciences, 926-68073, 1:10000).
Validation	Validations have been performed by the Manufacturer and can be seen on the Manufacturer's website or in the provided technical sheets. Positive and negative cell lines, Activator and Inhibitor Treatment, Tissue Type and Protocol Optimization has been conducted by the manufacturer and our lab to ensure the validity and the application of the following antibodies. (IF: Immunofluorescence and IB: Immunoblot analysis). Multiple lot numbers were used for antibodies over the course of the manuscript with no variation observed between lots. Cell Signaling Technology (CST) employs a set of strategies for antibody validation in each given application based on the approaches outlined in Uhlen, et al., Nature Methods, 2016 (https://www.nature.com/articles/nmeth.3995). This includes positive and negative cell lines/tissues, CRISPR/Knockdown, tissue arrays, treatments to induce/inhibit protein expression, comparison to other antibodies and purified recombinant proteins. Millipore validations done through immunoblot on human

fibroblast cells. Abcam validation done by a combination of chromogenic multiplexing, peptide blocking assays and knockdown/knockout studies in addition to BOND RX Validation. Invitrogen validation of pER (S118) antibody performed on human breast carcinoma tissues (IHC), HepG2 cells (IF) and MCF-7 cell lines (Immunoblot). Invitrogen Chi3l1 antibody underwent additional advanced validation within THP1 cells, mouse liver, ovary and spleen samples. Each lot of Biolegend antibodies are quality control tested by immunohistochemistry and validated on human tissue (skin, colon) and HepG2/THP1 cell lines

B-Actin, IB: 1/2000. Monoclonal Anti β-Actin antibody recognizes an epitope located on the N-terminal end of the β-isoform of actin. The antibody specifically labels β-actin in a wide variety of tissues and species using immunoblotting (42 kDa), immunofluorescent staining of cultured cell lines, and immunohistochemistry. Monoclonal Anti-β-Actin (mouse IgG1 isotype) is derived from the AC-15 hybridoma produced by the fusion of mouse myeloma cells and splenocytes from an immunized mouse. Species Reactivity: sheep, carp, feline, chicken, rat, mouse, Hirudo medicinalis, rabbit, canine, pig, human, bovine, guinea pig.

pan-AKT, IB: 1/1000. Akt (pan) (40D4) Mouse mAb detects endogenous levels of total Akt protein. This antibody does not cross-react with other related proteins. Monoclonal antibody is produced by immunizing animals with a synthetic peptide at the carboxy-terminal sequence of human Akt. Species Reactivity: Human, Mouse, Rat, Monkey.

pAKT (Ser473), IB: 1/500. Phospho-Akt1 (Ser473) (D7F10) XP® Rabbit mAb recognizes endogenous levels of Akt1 protein only when phosphorylated at Ser473. It does not detect Akt2 protein when phosphorylated at Ser474. Monoclonal antibody is produced by immunizing animals with a synthetic phosphopeptide corresponding to residues surrounding Ser473 of human Akt1 protein. Species reactivity: Human, Mouse, Rat

pAKT (Thr304), IB: 1/500. Phospho-Akt (Thr308) (244F9) Rabbit mAb detects endogenous levels of Akt only when phosphorylated at threonine 308. Monoclonal antibody is produced by immunizing animals with a synthetic phosphopeptide corresponding to residues around Thr308 of mouse Akt. Species Reactivity: Human, Mouse, Rat, Monkey.

AMPK, IB: 1/500. AMPKα (D5A2) Rabbit mAb detects endogenous levels of AMPKα protein. The antibody detects both the α1 and α2 isoforms of the catalytic subunit. Monoclonal antibody is produced by immunizing animals with a synthetic peptide corresponding to residues surrounding Arg21 of human AMPKα. Species reactivity: Human, Mouse, Rat, Monkey, Bovine.

pAMPK (Thr172), IB: 1/500. Phospho-AMPKα (Thr172) (40H9) Rabbit mAb detects endogenous AMPKα1 only when phosphorylated at threonine 183 and endogenous AMPKα2 only when phosphorylated at threonine 172. The antibody does not detect the regulatory β or γ subunits. Monoclonal antibody is produced by immunizing animals with a synthetic peptide corresponding to residues surrounding Thr172 of human AMPKα2 protein. Species Reactivity: Human, Mouse, Rat, Hamster, Monkey, D. melanogaster, S. cerevisiae. It is predicted to react with Chicken, Zebrafish, Bovine, and Pig based on 100% sequence homology.

CD206/ MRC1, IF: 1/400. Monoclonal antibody is produced by immunizing animals with a synthetic peptide corresponding to residues near the carboxy terminus of mouse CD206/MRC1 protein.CD206/MRC1 (E6T5J) XP® Rabbit mAb recognizes endogenous levels of total CD206/MRC1 protein. This antibody recognizes mouse CD206/MRC1 protein and is also reactive with human CD206/MRC1; however, this antibody is not suggested for immunohistochemical analysis of human tissues. Instead, CD206/MRC1 (E2L9N) Rabbit mAb #91992 is recommended for IHC analysis of human tissue samples. Species Reactivity:Human, Mouse, Rat, Monkey.

CD3-epsilon, IF: 1/200. Monoclonal antibody is produced by immunizing animals with a synthetic peptide corresponding to residues surrounding Val31 of mouse CD3ε protein.CD3ε (D4V8L) Rabbit mAb recognizes endogenous levels of total mouse CD3ε protein. Non-specific staining in mouse pancreas has been observed. CD3ε (D4V8L) Rabbit mAb may react weakly with human CD3ε, but is not suggested for use in immunohistochemical analysis of human tissues. Instead, CD3ε (D7A6E™) XP® Rabbit mAb #85061 is recommended for IHC analysis of human tissue samples. Species Reactivity: Mouse

CD4, IF: 1/200. Monoclonal antibody is produced by immunizing animals with a synthetic peptide corresponding to residues surrounding Ala232 of mouse CD4 protein.CD4 (D7D2Z) Rabbit mAb recognizes endogenous levels of total mouse and rat CD4 protein. Non-specific staining in mouse kidney and liver has been observed. Species Reactivity: Mouse, Rat, Hamster

CD8-alpha, IF: 1/200. Monoclonal antibody is produced by immunizing animals with a synthetic peptide corresponding to residues surrounding Asp42 of mouse CD8α protein.CD8α (D4W2Z) XP® Rabbit mAb recognizes endogenous levels of total CD8α protein. Species Reactivity: Mouse

Chi3l1, IB: 1/500. Recombinant fusion protein containing a sequence corresponding to amino acids 22-220 of human YKL-40/CHI3L1 (NP_0012672). Immunogen sequence: YKLVCYYTSW SQYREGDGSC FPDALDRFLC THIIYSFANI SNDHIDTWEW NDVTLYGMLN TLKNRNPNLK TLLSVGGWNF GSQRFSKIAS NTQSRRTFIK SVPPFLRTHG FDGLDLAWLY PGRRDKQHFT TLIKEMKAEF IKEAQPGKKQ LLLSAALSAG KVTIDSSYDI AKISQHLDFI SIMTYDFHGA WRGTTGHHS. Species Reactivity: Human, Mouse, Rat.

Cyclin D1, IB: 1/500. Cyclin D1 (92G2) Rabbit mAb detects endogenous levels of total cyclin D1 protein. Monoclonal antibody is produced by immunizing animals with a synthetic peptide corresponding to the carboxy-terminus of human cyclin D1. Species Reactivity: Human, Mouse, Rat.

Cytokeratin 5, IF: 1/100. This monospecific polyclonal antibody was raised against a peptide sequence derived from the C-terminus of the mouse keratin 5 protein. Each lot of this antibody is quality control tested by formalin-fixed paraffin-embedded immunohistochemical staining. Species reactivity: Human, Mouse, Rat.

Cytokeratin 8, IF: 1/100. This antibody was raised against the 12 carboxy-terminal amino acids of HK-8 preceded by a cysteine, CKLVSESSDVLPK. Each lot of this antibody is quality control tested by formalin-fixed paraffin-embedded immunohistochemical staining. Species Reactivity: Human, Mouse.

Cytokeratin 14, IF: 1/10. anti-Cytokeratin 14 (SP53) Rabbit Monoclonal Primary Antibody (this antibody) may be used as the primary antibody for immunohistochemical staining of formalin-fixed, paraffin-embedded tissue sections. Cytokeratin 14 (SP53) Rabbit Monoclonal Primary Antibody is intended for laboratory use in the detection of the Cytokeratin 14 protein in formalin-fixed, paraffin-embedded human tissue stained in qualitative immunohistochemistry (IHC)

on BenchMark IHC/ISH instruments but has been validated for use in immunofluorescent staining on mouse tissue by our lab.

E-cadherin, IB: 1/1000, IF: 1/200. The 36/E-Cadherin monoclonal antibody recognizes the cytoplasmic domain of E-Cadherin regardless of phosphorylation status. The peptide immunogen was generated from human E-Cadherin aa. 735-883. Investigators are advised that this antibody has some degree of cross-reactivity to P-Cadherin. Species Reactivity: Human (QC Testing), Mouse, Rat, Dog (Tested in Development). Validated in 293F cell lines via transfection using both IHF and WB.

ERα, IF: 1/200. ERα Antibody (MC-20) is a rabbit polyclonal IgG with the epitope mapping at the C-terminus of ERα of mouse origin. This antibody has since been discontinued and replaced with Estrogen Receptor alpha (F-10): sc-8002.

pER (Ser118), IB: 1/500. A synthesized peptide derived from human ESR1(Accession P03372), corresponding to amino acid residues around phosphorylated Ser118. Antibody detects endogenous levels of Estrogen Receptor alpha only when phosphorylated at Serine 118. Species Reactivity: Human, Mouse.

EpCAM, IF: 1/400, IB: 1/1000. EpCAM (E6V8Y) XP® Rabbit mAb (Mouse Preferred) recognizes endogenous levels of total EpCAM protein. This antibody is preferred for immunohistochemical analysis of mouse tissues. For human tissue analysis, EpCAM (D9S3P) Rabbit mAb (IHC Preferred) #14452 is suggested. Monoclonal antibody is produced by immunizing animals with a synthetic peptide corresponding to residues surrounding Arg311 of mouse EpCAM protein. Species Reactivity: Human, Mouse, Rat, Hamster.

F4/80, IF: 1/200. Monoclonal antibody is produced by immunizing animals with recombinant mouse F4/80 protein.F4/80 (D2S9R) XP® Rabbit mAb recognizes endogenous levels of total F4/80 protein. Species Reactivity: Mouse

GATA3 (Santa Cruz), IF: 1/100. GATA3 Antibody (HG3-31) is a mouse monoclonal IgG1 κ GATA3 antibody, raised against recombinant GATA-3 of human origin. Species Reactivity: Mouse, Rat, Human and Avian origin. Validated in Jurkat and MOLT-4 cells for both IHC and WB.

GATA3 (Cell Signalling), IB: 1/1000. GATA-3 (D13C9) XP® Rabbit mAb recognizes endogenous levels of total GATA-3 protein. Monoclonal antibody is produced by immunizing animals with a synthetic peptide corresponding to residues surrounding Tyr63 of human GATA-3 protein. Species Reactivity: Human, Mouse. Predicted to react with Monkey based on 100% sequence similarity.

Granzyme B, IF: 1/100. Granzyme B (E5V2L) Rabbit Monoclonal Antibody recognizes endogenous levels of total mouse Granzyme B protein. This antibody does not cross-react with human Granzyme B proteins. Non-specific staining was observed in mouse kidney. Species Reactivity: Mouse.

HER2 (DAKO), IF: 1/400. Synthetic human c-erbB-2 oncoprotein peptide from the intracytoplasmic part of the c-erbB-2 oncoprotein. The peptide was coupled to keyhole limpet hemocyanin (KLH). The antibody labels an intracellular domain of c-erbB2 oncoprotein. Species Reactivity: Human, but has also been observed to react with mouse using immunofluorescent staining within our lab.

HER2 (Cell Signalling), IB: 1/500. HER2/ErbB2 (29D8) Rabbit mAb detects endogenous levels of total ErbB2 protein. This antibody does not cross-react with related kinases. Monoclonal antibody is produced by immunizing animals with a synthetic peptide corresponding to residues surrounding tyrosine 1248 of human ErbB2 protein. Species Reactivity: Human, Mouse. Predicted to react with rat based on 100% sequence similarity.

pHER2 (Tyr1221/1222), IB: 1/500. Phospho-HER2/ErbB2 (Tyr1221/1222) (6B12) Rabbit mAb detects endogenous levels of ErbB2 only when phosphorylated at tyrosines 1221/1222. The antibody does not detect other activated Erb family members or other tyrosine-phosphorylated proteins. Monoclonal antibody is produced by immunizing animals with a synthetic phosphopeptide corresponding to residues surrounding tyrosines 1221/1222 of human ErbB2 protein. Species Reactivity: Human.

HER3, IB: 1/500. HER3/ErbB3 (D22C5) XP® Rabbit mAb recognizes endogenous levels of total HER3/ErbB3 protein. This antibody does not cross-react with other HER family proteins. Monoclonal antibody is produced by immunizing animals with recombinant protein corresponding to the carboxy terminus of human ErbB3 protein. Species Reactivity: Human, Mouse.

pHER3 (Tyr1289), IB: 1/500. Phospho-HER3/ErbB3 (Tyr1289) (21D3) Rabbit mAb detects endogenous HER3/ErbB3 proteins only when phosphorylated at tyrosine 1289. This antibody cross-reacts with overexpressed EGFR. This antibody may cross-react with overexpressed receptor tyrosine kinases in IHC. Monoclonal antibody is produced by immunizing animals with a synthetic phosphopeptide corresponding to residues surrounding Tyr1289 of human HER3/ErbB3. Species Reactivity: Human, Mouse. Predicted to react with Rat and Dog based on 100% sequence similarity.

Ki67, IF: 1/200. Monoclonal antibody is produced by immunizing animals with a recombinant protein specific to the amino terminus of Ki-67 protein.Ki-67 (D3B5) Rabbit mAb (IHC Formulated) recognizes endogenous levels of murine Ki-67 protein. It will also detect endogenous levels of human Ki-67 protein; however, Ki-67 (D2H10) Rabbit mAb #9027 is recommended for the detection of human Ki-67 protein in paraffin-embedded tissues. Species Reactivity: Mouse.

Ly6G, IF: 1/100. Ly-6G (E6Z1T) Rabbit mAb recognizes endogenous levels of total Ly-6G protein. This antibody does not cross-react with Ly-6C1, Ly-6C2, Ly-6F, or Ly-6I. Non-specific staining was observed in mouse gastrointestinal epithelium by immunohistochemistry. Monoclonal antibody is produced by immunizing animals with mouse Ly-6G recombinant protein. Species Reactivity: Mouse.

MAPK, IB: 1/1000. p44/42 MAPK (Erk1/2) Antibody detects endogenous levels of total p44/42 MAP kinase (Erk1/Erk2) protein. In some cell types, this antibody recognizes p44 MAPK more readily than p42 MAPK. The antibody does not recognize either JNK/SAPK or p38 MAP kinase. Polyclonal antibodies are produced by immunizing animals with a synthetic peptide corresponding to a sequence in the C-terminus of rat p44 MAP Kinase. Antibodies are purified by protein A and peptide affinity chromatography. Species Reactivity: Human, Mouse, Rat, Hamster, Monkey, Mink, Zebrafish, Bovine, Pig, S. cerevisiae.

pMAPK (Thr202/Tyr204), IF: 1/400, IB: 1/500. Phospho-p44/42 MAPK (Erk1/2) (Thr202/Tyr204) (D13.14.4E) XP® Rabbit mAb detects endogenous levels of p44 and p42 MAP Kinase (Erk1 and Erk2) when dually phosphorylated at Thr202 and Tyr204 of Erk1 (Thr185

and Tyr187 of Erk2), and singly phosphorylated at Thr202. This antibody does not cross-react with the corresponding phosphorylated residues of either JNK/SAPK or p38 MAP kinases. Monoclonal antibody is produced by immunizing animals with a synthetic phosphopeptide corresponding to residues surrounding Thr202/Tyr204 of human p44 MAP kinase. Species Reactivity: Human, Mouse, Rat, Hamster, Monkey, Mink, D. melanogaster, Zebrafish, Bovine, Dog, Pig, S. cerevisiae. Predicted to react with Chicken, and C. elegans based on 100% sequence homology.

c-Myc, IB: 1/500. c-Myc (E5Q6W) Rabbit mAb recognizes endogenous levels of total c-Myc protein. Monoclonal antibody is produced by immunizing animals with recombinant protein specific to the amino terminus of human c-Myc protein. Species Reactivity: Human, Mouse, Rat.

Myeloperoxidase, IF: 1/500. Anti-Myeloperoxidase antibody [EPR20257] is a rabbit recombinant monoclonal antibody that is used to detect Myeloperoxidase in Flow cytometry (Intra), ICC/IF, IHC-P, Western blot. Specific to Myeloperoxidase heavy chain but the exact immunogen used to generate this antibody is proprietary information. Species Reactivity: Human, Mouse, Rat.

Neutrophil Elastase, IF: 1/200. Neutrophil Elastase (E8U3X) Rabbit mAb recognizes endogenous levels of total neutrophil elastase protein. This antibody does not cross-react with human neutrophil elastase protein. Non-specific staining was observed in the vas deferens by immunohistochemistry. Monoclonal antibody is produced by immunizing animals with full-length recombinant protein specific to mouse neutrophil elastase protein. Species Reactivity: Mouse.

p-Stat1 (Y701), IF: 1/200. Monoclonal antibody is produced by immunizing animals with a synthetic phosphopeptide corresponding to residues surrounding Tyr701 of human Stat1.Phospho-Stat1 (Tyr701) (58D6) Rabbit mAb detects endogenous levels of Stat1 only when phosphorylated at tyrosine 701. The antibody detects phosphorylated tyrosine 701 of p91 Stat1 and also the p84 splice variant. It does not cross-react with the corresponding phospho-tyrosines of other Stat proteins. Species Reactivity: Human, Mouse

Stat3, IB: 1/1000. Stat3 (124H6) Mouse mAb detects endogenous levels of total Stat3 protein. Monoclonal antibody is produced by immunizing animals with a synthetic peptide centered around amino acid Gln692 of human Stat3. Species Reactivity: Human, Mouse, Rat, Monkey.

pStat3 (Tyr705), IB: 1/500. Phospho-Stat3 (Tyr705) (D3A7) XP® Rabbit mAb detects endogenous levels of Stat3 only when phosphorylated at tyrosine 705. This antibody does not cross-react with phospho-EGFR or the corresponding phospho-tyrosines of other Stat proteins. Monoclonal antibody is produced by immunizing animals with a synthetic phosphopeptide corresponding to residues surrounding Tyr705 of mouse Stat3. Species Reactivity: Human, Mouse, Rat, Monkey. Predicted to react with Hamster, Bovine, Pig, and Horse based on 100% sequence homology.

PD1, IF: 1/200. PD-1 (Intracellular Domain) (D7D5W) Rabbit Monoclonal Antibody recognizes endogenous levels of total PD-1 protein. Reactivity: Mouse. Predicted to react with Hamster and Rat based on on 100% sequence homology.

Vinculin, IB: 1/5000. Mouse Monoclonal. Anti-Vinculin Antibody, clone VIIF9 (7F9) is an antibody against Vinculin for use in IP, WB, IC, IH(P). Species reactivity: Mouse, human, pig, rabbit, monkey, bovine

IRDye 800CW Donkey anti-Rabbit, IB: 1/10000. Rabbit IgG. The antibody was isolated by affinity chromatography using antigens coupled to agarose beads. Based on ELISA, this antibody reacts with the heavy and light chains of rabbit IgG, and with the light chains common to most rabbit immunoglobulins. This antibody was tested by ELISA and/or solid-phase adsorbed to ensure minimal cross-reactivity with bovine, chicken, goat, guinea pig, hamster, horse, human, mouse, rat, and sheep serum proteins, but may cross-react with immunoglobulins from other species. The conjugate has been specifically tested and qualified for Western blot and In-Cell Western™ Assay applications.

IRDye 680RD Donkey anti-Mouse, IB: 1/10000. Mouse IgG. The antibody was isolated by affinity chromatography using antigens coupled to agarose beads. Based on immunoelectrophoresis, this antibody reacts with the heavy chains of mouse IgG, and with the light chains common to most mouse immunoglobulins. No reactivity was detected against non-immunoglobulin serum proteins. This antibody was tested by ELISA and/or solid-phase adsorbed to ensure minimal cross-reactivity with bovine, chicken, goat, guinea pig, Syrian hamster, horse, human, rabbit, and sheep serum proteins, but may cross-react with immunoglobulins from other species. The conjugate has been specifically tested and qualified for Western blot and In-Cell Western™ Assay applications.

All antibodies were tested on the mouse (species) samples. Additionally, CK8 and HER2 antibodies were also tested on human samples.

Eukaryotic cell lines

Policy information about cell lines and Sex and Gender in Research

Cell line source(s)

Cells were cultured in a humidified, 5% CO_2, 37°C incubator in complete media. UACC812 (ATCC, CRL-1897), SK-BR-3 (ATCC, HTB-30), JIMT-1 (Accegen, ABC-TC504S) and NMuMG (ATCC, CRL1636) cells were grown and maintained in DMEM (Wisent, 511-016-UG), supplemented with 10% fetal bovine serum (FBS) and 1% penicillin/streptomycin (Wisent, 450-200 EL), as well as 5µg/ml human insulin. ZR-75-30 (ATCC, CRL-1504) cells were maintained in RPMI 1640 (Wisent, 350-060 CL), supplemented with 10% FBS and 1% penicillin/streptomycin. SUM225CWN cells (BioIVT, HUMANSUM-000301) were maintained in HAM's F-12 (Wisent, 318-011 CL), supplemented with 5% FBS, 10mM HEPES, 1µg/ml hydrocortisone and 5µg/

	ml insulin. NMuMG/HER2 and NMuMG/HER2Δ16 cell lines were transfected with either pMSCV-HER2 or pMSCV-HER2Δ16 plasmids using Genejuice (Novagen 70967), and selected with 2µg/ml puromycin (Clontech 631305) as published previously22. UACC812 lapatinib resistant (LR) cells were established by exposing UACC812 parental cells to increasing concentrations of lapatinib over multiple passages, until no further cell death was observed, and maintained in 2µM lapatinib in culture. Cell cultures were routinely monitored for contamination, passaged once they reached 80-90% confluency and used at early passages. Regular testing of cells for mycoplasma using the MycoAlert Kit (Lonza, LT07-118) confirmed that all cell lines used in this study were negative for mycoplasma contamination.
Authentication	NMuMG cells transfected with human HER2 or HER2Δ16 and developed within our lab undergo confirmation of transgene expression through qRT-PCR and/or BaseScope detection with species-specific primers recognizing either the wildtype or splice-isoform exon-junctions (validation can be observed in Figure 2). Human cell lines purchased through ATCC (ZR7530, JIMT, SKBR3, UACC812) were not authenticated by our lab but by the provider, however expression of HER2 was confirmed via qRT-PCR and/or BaseScope detection using species-specific primers for the wildtype or splice-isoform exon-junctions (validation can be observed in Supplementary Figure 8). SUM225CWN cells received authentication by BioIVT prior to purchase, are grown in antibiotic free medium and monitored for bacterial and/or mycoplasma contamination. JIMT-1 cells received authentication by Accegen prior to purchased are grown in antibiotic free medium and monitored for bacterial and/or mycoplasma contamination.<br><br>Lapatinib resistant cell lines are confirmed to retain resistance via exposing cells to increasing concentrations of lapatinib over multiple passages, until no further cell death is observed, and maintaining cells in 2µM lapatinib in culture.
Mycoplasma contamination	All cells used in this study were negative for mycoplasma contamination tested using the MycoAlert Kit (Lonza, LT07-118).
Commonly misidentified lines<br>(See ICLAC register)	This study did not use any commonly misidentified lines.

Animals and other research organisms

Policy information about studies involving animals; ARRIVE guidelines recommended for reporting animal research, and Sex and Gender in Research

Laboratory animals	Mice were maintained within the specific pathogen-free (SPF) animal research facility at the Goodman Cancer Institute, housed in autoclaved cages with ad libitum access to food and water as well as sufficient nesting and enrichment materials. Cages were kept on ventilated racks under a 12 h light cycle at a temperature of 20-24 °C and relative humidity of 45-65%.<br><br>The TetO-ErbB2/Her2(human)-IRES-Cre (EIC), TetO-ErbB2Δ16IC/HER2Δ16(human)-IRES-Cre (Δ16IC), MMTV-Cre, MMTV-rtTA (MTB) and HER2flxExon16 (FE16) transgenic strains were bred and maintained on a pure FVB/N background (Charles River, FVB/Ncrl; Strain code: 207). Genomic DNA was extracted from tails of all mice using crude salt extraction and subsequently used for genotype confirmation using PCR.<br><br>At 8-12 weeks of age, doxycycline (2mg/mL, Wisent) was administered to bigenic mice via the drinking water to activate the reverse tetracycline transactivator (rtTA) transgene and drive expression of EIC or Δ16IC downstream from the TetO promoter specifically in the mammary epithelium. Experimental mice were monitored for tumor formation by weekly palpation and caliper measurements post-doxycycline induction. All mice were housed and handled at the Comparative Medicine and Animal Resource Centre in accordance with McGill University Animal Ethics Committee guidelines.<br><br>For allograft studies, mice were maintained on a pure FVB/N background (Charles River, FVB/Ncrl; Strain code: 207) and underwent surgery at 8-10 weeks of age.
Wild animals	This study did not use any wild animals.
Reporting on sex	This study takes into consideration that breast cancer predominantly affects women although we acknowledge that 1% of cases occur in men. Therefore, our study solely uses female experimental mice to recapitulate breast cancer in women. All experimental mice were females, on the FVB/N background. No data was collected on gender.
Field-collected samples	The study did not involve samples collected from the field.
Ethics oversight	Experiments involving mice were conducted in accordance with McGill University and Canadian Council on Animal Care (CCAC) ethical guidelines under a protocol (MCGL-5518) approved by the McGill University Downtown Campus Facility Animal Care Committee (FACC), a branch of the McGill University Animal Care Committee (UACC), Montreal, QC, Canada.

Note that full information on the approval of the study protocol must also be provided in the manuscript.

Plants

Seed stocks	no plant specimens were used in this study.
Novel plant genotypes	no plant specimens were used in this study.
Authentication	no plant specimens were used in this study.
