# Supplementary material for: HER2∆16 directs luminal cell identity and estrogen receptor signaling in HER2+ breast cancer
Source: Nat Commun. 2026 Jun 15;17:7532. doi: 10.1038/s41467-026-74435-9 (PMC13408682; doi:10.1038/s41467-026-74435-9)
Supplement: Supplementary file 3 — Transparent Peer Review file [file 41467_2026_74435_MOESM3_ESM.pdf]

nature portfolio

Peer Review File

HER2Δ16 directs luminal cell identity and estrogen receptor signaling in HER2+ breast cancer

Corresponding Author: Professor William Muller

This file contains all reviewer reports in order by version, followed by all author rebuttals in order by version.

Version 0:

Reviewer comments:

Reviewer #1

(Remarks to the Author)
This manuscript is a research article from Hailey Proud et al., which investigates the role of HER2 isoforms (more specifically HER2Δ16) in driving luminal cell identity, estrogen receptor (ER) expression, signaling, and endocrine response in breast cancer. The primary model adopted to carry out this investigation was based on syngeneic MMTV-HER2 genetically-modified mouse models (Control (full length) and HER2Δ16). The results suggest that HER2Δ16 drives the early onset of primary tumor while increasing lung metastasis incidence. These results corroborate previous studies as summarized in the following article: (PMID: 31261614). However, a more surprising observation of this manuscript derives from the phenotypic and molecular analyses demonstrating increased luminal phenotype of HER2Δ16 tumors. Overall, the manuscript is well-written but has various limitations that need to be addressed.
General comments:
1- The association of HER2Δ16 with ER signaling has been well-established in the manuscript. While cross-talk between the two factors is well-accepted, the mechanisms contributing to ER induction remain elusive in the study. Moreover, the co-expression of full-length HER2 and HER2Δ16 seems determinant in the ER phenotypic outcome.
2- Many results presented in the manuscript are confirmatory (e.g., Fig. 1). This includes experiments related to HER2 targeting.
3- Based on immunofluorescence images, it is clear that both control and HER2Δ16 tumors are expressing ER, despite the pronounced increase in the latter. Therefore, if pathological analyses were to be performed, both tumors would likely be classified as HER2+/ER+. While the manuscript mainly investigates the increased ER signaling, it remains intriguing how HER2Δ16 promotes both the luminal and EMT phenotype of cancer cells. Moreover, a dominant effect seems to occur in conditions where both full-length and HER2Δ16 are expressed.
Specific comments:
4- Fig.1 confirms the association of d16 with early onset and worse survival outcomes.
5- Fig 2GTamoxifen treatment reduces HERD16-expressing cell growth to the level of HER2. Therefore, using this as a biomarker for endocrine response remains limited
6- Fig.2a: Provide higher resolution images to help determine the localization of ER in tumors
7- Fig.4: Immune cells appear to increase in tamoxifen-treated mice. However, to ascertain that the phenotypic change exerts anti-tumor responses, functional assessment needs to be performed.
8- Figure 7 proposes an estrogen-independent mechanism or ER signaling activation in HER2Δ16 tumors. However, to what extent is the HER2Δ16-mediated ER signaling relevant in estrogen-independent conditions (e.g., menopause)? This can be tested in vivo and validated in vitro. Additionally, it is unclear how HER2Δ16 promotes ER expression. Based on qPCR experiments, ER may be transcriptionally regulated in HER2Δ16 tumors. Exploring the mechanism of activation will bring mechanistic novelty to this manuscript.
9- Is the mechanism of ER activation maintained in heterozygous conditions? If yes, this needs to be included in the summary figure (Fig.7c). Moreover, how does the author reconcile these findings with the negative crosstalk often observed between ER and Her2?
10- Erbb4 increased in HER2D16 tumors, suggesting a possible impact on ER signaling. How do you uncouple this from the direct impact of HER2D16 on ER signaling and increased luminal phenotype?

Reviewer #2

(Remarks to the Author)
In this manuscript the authors show that HER2Δ16 expression in MMTV-driven GEMM and knock-in mouse mammary tumor models promotes a shift to luminal differentiation states and shifts tumors toward ER-driven signaling, making the cells sensitive to endocrine therapy. They also show that this shift is also observed in human HER2+ breast cancer cell lines upon resistance to lapatinib, suggesting a compensatory reliance on ER signaling. Mouse mammary tumor models are generally ER negative and not dependent on estrogen signaling. Demonstrating the ER+ phenotype and ER dependency of this model would make it potentially useful for studies of ER+ breast cancer. Thus, the manuscript could be a valuable addition to the field. However, the ER positivity and dependency of the model is not conclusively proven.

Major comments
1. The authors show ER expression only by Esr1 RNA ISH, not at the protein level. They have to perform ER immunostaining on the tumors and/or immunoblot analyses.

2. Endocrine therapy can inhibit mammary tumor growth even in ER-negative models through the microenvironment, especially immune cells. Thus, to prove that the effect of tamoxifen is through targeting ER in the tumor cells the authors have to delete Esr1 in the model and also use ER degraders – tamoxifen is not a full ER antagonist.

3. A characterization of the FE16 wt/fx model should be done more thoroughly. The authors specify that “heterozygous deletion of exon 16 in this model is not sufficient to promote mammary tumorigenesis without additional pro-oncogenic mutations” (line 370), but some mice developed tumors with the same latency as the fx/fx. What other mutations does these tumors have then? What is the HER2 expression and amplification status in the FE16 wt/fx tumors. What proportion of ErbB2 wt and Δ16 is there? If HER2 Δ16 expression is intermediate compared to the fx/fx, what is the ER, CK8, EpCAM expression?

4. In the model with co-expression of full-length HER2 and ErbB2Δ16, what is the expression levels of both HER2 full-length and Δ16? Does it resemble what is present in the human cases (HER2Δ16-positive breast cancer cases, HER2Δ16 makes up 4-10% of total HER2 transcript).

5. For the lapatinib resistant model, is lapatinib selecting for cells with a prior higher expression of HER2Δ16? Is there a population already expressing higher levels of HER2Δ16 ER in these cells?

6. Fig. 7A, there is no total ER blotted. The levels of phospho-ER should be normalized to it. As its expression is higher in the HER2Δ16 tumors, the ratio may not be different, and the conclusion of an increased phosphorylation cannot be made. Same thing for all phosphorylation assessment, the quantification should be phosphorylated over total.

7. In cells in vitro (NMuMG) and in human resistant models, is there an increase of ERK signaling? Is this signaling important for the increase of ER expression, rather than its phosphorylation? If MAPK pathway is inhibited, would ER expression or phosphorylation change?

Minor comments
1. M1 and M2 macrophage nomenclature is outdated, it would be better to refer as pro- and anti-tumoral or indicate the marker expression.

2. Throughout the paper, the microscopy figures would benefit from including a zoomed inset (especially where it is required to for the conclusions like in Fig.1J, Fig. 3D).

3. Throughout the paper, it will be good to harmonize font size between panels. Some have text is too small to read properly (ex. qPCR bar graph).

4. Line 329 – reference to Supplementary Fig. 2A should be Supplementary Fig. 2B

Reviewer #3

(Remarks to the Author)
Summary
In this paper the authors study transgenic models of d16HER2-induced tumorigenesis and report that it has transcriptional differences with the wtHER2-induced tumors. Most importantly they report that d16HER2-induced tumors have active estrogen receptor signaling and take this further into compound transgenic models and human tumor cells lines to suggest that the d16HER2 transcript of HER2 underlies estrogen receptor expression and signaling and this underlies resistance to HER2 inhibitors and sensitization to tamoxifen.

General criticism
There is a lot of work done in this paper and a lot of time spent generating and studying these mice. I don’t have specific criticisms of the methodology and techniques. The experiments are well done and the data looks good. I am not going to offer criticisms of specific experiments and data items because I don’t think it’s relevant. My criticism is about the background, context, interpretation, relevance, and conclusions and lack of scientific objectivity. This paper continues along

an exhausted line of nearly three decades of a failed narrative to promote a role for d16HER2 in human cancer biology and therapeutics. The problem is that these are experiments that produce experimental results, many of which conflict with eachother, many that conflict with clinical evidence, and the reality is that the experimental work is disconnected from and not relevant to human cancer biology and therapeutics. This is merely experimental science for the purpose of experimental science. I think the conclusions of this work regarding the biology of d16HER2 in human cancers and its implications for the treatment of human cancers are unjustified.

d16HER2 narrative
The d16HER2 splice variant has been the subject of nearly three decades of studies. Many papers have now shown that d16HER2 is a more potent oncogene than wtHER2. There is no doubt about this experimental finding. The problem is that this experimental finding is not relevant to human cancer biology because these artifactual experimental models do not reproduce the human disease. The transgenic mouse model of d16HER2 mammary tumorigenesis has previously been published and develops tumors with accelerated kinetics compared to wtHER2 (PMID 21559085)(PMID 34131071)(PMID 27157621). So this is already well established. But while these models tell us about the high oncogenic potency of d16HER2, they do not inform us about human cancer biology, because the human cancers do not express d16HER2 at these high levels and in isolation. In fact this current paper by Proud et al does provide an important contribution, and that is the generation of a mouse transgenic model that uses Cre-lox to convert the endogenous Neu gene to an ex16-deleted version in mammary tissue, and this turns out NOT to be tumorigenic. This is despite the spin put on it here that in the homozygous genotype, and after several years of age, a very small subset of mice develop tumors (Figure 3). And these small subsets again have developed amplification and overexpression of d16HER2 and no longer are representative of human tumors which have low levels of d16HER2. Their compound genetic model of wtHER2 overexpression and d16HER2 knock-in shows that the co-expression of d16HER2 actually retards tumorigenesis, also arguing against a driver or accelerator role (Suppl Fig 4). The important contribution of these newer genetic models is that it confirms the artifactual nature of the d16HER2 transgene overexpression models and its irrelevance to human cancer. It shows that the low amount of d16HER2 in the HER2-amplified human cancers is not the tumorigenic driver, and as always suspected, the wtHER2 overexpression is the driver of the human disease. I commend the authors for performing this experiment, and I suggest the honest interpretation of the findings, rather than continued promotion of a false d16HER2 narrative.

Conflicting literature and lack of clinical relevance
This paper reports that d16HER2 expression is associated with induction of ESR1 and ER signaling. But in a clinical study of HER2 positive breast cancers, the higher co-expression of d16HER2 was associated with an ER negative subtype (PMID 19671734), and in another microarray gene expression study of a large number of breast tumors the expression of d16HER2 was associated with the ER negative state (PMID 23867476). In in vitro transfection studies of MCF7 cells, another group found that expression of d16HER2 reduces ER expression and induces endocrine resistance (PMID 20876285). The Muller group itself has previously done gene expression analysis of the d16HER2 transgenic mice and reported years ago that these tumors have gene expression profiles that resemble the basal subtype of human breast cancers, not the luminal ER positive type (PMID 27157621). All these data contradict the suggestions of this current paper by Proud. This study reports that d16HER2 expression induces ER expression and confers sensitivity to tamoxifen. There are other experiments reported where d16HER2 expression seems to confer resistance to tamoxifen (PMID 20876285)(PMID 21172025). This study by Proud reports that d16HER2 expression, by virtue of inducing ER signaling, confers resistance to HER2 targeted therapy. But other studies have reported that d16HER2-induced mouse tumors were more sensitive to trastuzumab compared to wtHER2-driven tumors (PMID 28903354), and a clinical study found a positive correlation between d16HER2 and trastuzumab response (PMID 25164009). All these findings contradict the conclusions of this current paper by Proud. Now I am not saying that all these other papers are right, and this paper by Proud et al is wrong. There are other d16HER2 papers with more disparate results and other conclusions. The point is that this field of d16HER2 experimental science is fraught with experimental artifactual findings, many which are conflicting with eachother, but more importantly many and most of the findings are tied to that specific experimental model and technique and are detached from and have no relevance to the human disease. This current paper by Proud is just another experimental d16HER2 data set and perhaps should be placed in the public domain for others to see, but it simply piles on top of a heap of studies, internally conflicting, all of which are almost surely irrelevant to human HER2-driven breast cancers.

Suggestion that mutations in mouse models are evidence of clinical relevance
These and other authors often cite the development of mutations in the mouse transgenic models as a correlate of and as supporting evidence for the importance of the d16HER2 variant in the human cancers. Juxtamembrane region deletions do develop in Neu transgenic mice (PMID 7935422) and huHER2 transgenic mice (PMID 15073130) as referenced here (line 358). But they develop in only half the tumors. So the other half of these tumors do not have such mutations and are driven by wildtype Neu or HER2. While it is reasonable to suggest that such mutations confer an advantage to the transgene, it is incorrect to say that overexpression of the wildtype genes is insufficient and to suggest that mutations are required to drive tumorigenesis (line 358) since half of such tumors do not have these mutations. At this time, these mutations are a peculiarity of the mouse models and not known to be required for tumorigenesis and the human tumors do not have such mutations, and attribution of these mouse mutations as a correlate of d16HER2 variant in human tumors is unjustified, and the newer genetic models in this paper actually confirm that.

d16 is HER2 baggage
The reality is that the HER2 gene is amplified and massively overexpressed in cancers. Along with such massive overexpression comes a lot of baggage; ie many events that ordinarily are low level events are also increased in parallel. This includes increased expression of alternative isoforms and truncation products such as d16HER2 and p95HER2, it includes promiscuous interaction partners not normally seen at low expression levels, it includes abnormal localization events, and it includes abnormal protein processing, glycosylation and other modifications. All these events are

consequences of massive overexpression of this gene and protein product. Many papers have been published about all these events that are much more pronounced and better detected in HER2-amplified cancers and many have suggested that these associated events underly the tumorigenic attributes of this oncogene. But all this body of work has been speculative, and an essential role for any of these “baggage” events has never been demonstrated, and it remains uncontested that overexpression of wtHER2 is sufficient for tumorigenesis and wtHER2 remains the principle form of this target that is relevant for therapeutics.

Reviewer #4

(Remarks to the Author)
I co-reviewed this manuscript with one of the reviewers who provided the listed reports. This is part of the Nature Communications initiative to facilitate training in peer review and to provide appropriate recognition for Early Career Researchers who co-review manuscripts.

Version 1:

Reviewer comments:

Reviewer #1

(Remarks to the Author)
The manuscript has improved, and several concerns have been addressed. However, as noted in the author responses, additional experiments are required before the manuscript can be considered for publication. In particular, the following comments must be addressed:
1- Experiments proposed in response to Comment #7 need to be carried out.
2- Response to Comment #8: Although the link between d16Her2 and ER activity (via MAPK) has been proposed, the mechanism driving the increased expression of ER in d16 tumors remains unclear. Since the d16-mediated luminal switch is a key finding, this issue must be addressed or acknowledged as a study limitation.
3- Experiments proposed in response to Comment #10 need to be completed.

Minor comments: The font size used for most figure annotations is very small. Improving figure readability would be beneficial.

Reviewer #2

(Remarks to the Author)
The authors have responded to each of the specific comments by the reviewers. The revised manuscript is significantly improved.

Reviewer #3

(Remarks to the Author)
The rebuttal arguments and revised manuscript do not change my opinion regarding this work.

In the rebuttal, the authors provide references that show d16HER2 detection in clinical samples and clinical correlations between d16HER2 and tumor aggressiveness, LN metastasis, and long term outcomes. These have been repeatedly stated for decades now, and perhaps at initial discovery decades ago, correlative data formed a preliminary basis to suggest a hypothesis regarding the functional importance of d16HER2 in human cancer biology. But no such proof has emerged, and this hypothesis has failed the test of time, and only greatly artifactual and non-representative experimental scientific models have been produced that function to push a false agenda. The clinical correlations can be due to a myriad of other factors such as HER2 levels, amplicon architecture, other tumor gene mutations, and many other factors. The rebuttal also cites evidence from other cancers including NSCLC and gastric cancers reporting d16HER2 expression, but they fail to mention that these are all also cases of HER2 amplification which constitutes the primary disease or resistance driver. These reports do nothing to strengthen the mechanistic hypothesis being pushed. The best summation of all the clinical and experimental evidence to date is that d16HER2 exists, but does not have biologically meaningful relevance as a disease driver or therapeutic target and the amplification and overexpression of HER2 is the principal disease driver. The current paper by Proud et al merely adds to the mass of inconclusive data in this realm. It should be placed in the public domain for the record, but I’m afraid there is nothing impactful to be gained by this work.

The rebuttal continues to argue for the case that this paper shows d16HER2 is a tumorigenic structural alteration, citing Fig 3 knockin model. But this again required the 50-fold gene amplification and massive overexpression of d16HER2 (Fig 3G), clearly demonstrating that the d16 structural event by itself was not sufficient to drive tumorigenesis. As is already very well established, overexpression is the transforming event of the HER2 oncogene and this knock-in experiment simply re-proved this already established hypothesis and disproved their contention that d16 deletion is a transforming alteration. The literature now has many mouse genetic models of this oncogene. It is very clear that the overexpression of the wildtype HER2/ERBB2 is tumorigenic, and overexpression of variant mutant forms is also tumorigenic, albeit with faster kinetics, however mutational alteration by itself is not tumorigenic (PMID 1359541, 15073130, 19364815, 21559085, 25164009). In the rebuttal the authors assert that the long latency and low penetrance of the genotype-phenotype should not disqualify this

mutation as a tumor driver. But it is not the latency and penetrance alone that disqualify the driver status; rather it is the required HER2 gene amplification that develops and that is the unimpeachable disease driver that cannot be ignored. In contrast to over-expression systems, the mutational knock-in of the endogenous ERBB2 (without overexpression) is not tumorigenic. The Muller group themselves have previously published such a model in 2000. They generated a knock-in mouse genetic model to introduce the activating point mutation in the transmembrane domain of ERBB2 and these mice developed tumors only late in life but importantly these were shown to have amplification of the mutant ERBB2 allele (PMID 10716706). The current d16HER2 work shows very similar results. In the previous work in 2000 they correctly concluded that the TM mutation is insufficient for tumorigenesis whereas in the current work they are attempting to spin the same negative result with the d16HER2 mutation work to suggest that the d16HER2 alteration is sufficient to drive tumorigenesis. I suggest the honest interpretation of the current work, which is that d16HER2 alteration is insufficient to drive tumorigenesis. I realize that the world of academia unfortunately does not reward negative results and that is a flaw of the system. But negative results are important, sometimes just as important as positive results. This work shows that d16HER2 is not tumorigenic by itself and the accompanying HER2 gene amplification is essential and sufficient for tumorigenesis. This is an important and revealing negative result. I suggest the honest conclusion of the work rather than the continued propagation of a false narrative.

The rebuttal tries to defend their assertion in the paper the somatic TM mutations observed in the mouse models of Neu tumorigenesis are also seen in human cancers. The incidence of HER2 mutations is now a well studied fact. A multitude of studies have determined the percentage of HER2-amplified breast cancers that also harbor somatic HER2 mutations and show that it’s rare, in the range of 1-2% and they are almost entirely within the hotspot regions of kinase domain and S310 (PMID 28164408)(PMID 27697991)(PMID 30301790)(and other studies). In their rebuttal, Proud et al highlight a really poor study of ctDNA (not tumor assays) reporting an exorbitantly high number of mutations spanning the entire HER2 gene, almost surely false, to support their claim that there are frequent mutations in the juxtamembrane region in HER2-amplified tumors as well (PMID 33145402). To rely on a false study to support the premise of your work does not engender credibility to the work. The scientific literature is so massive that you can do a google search and come up with virtually anything you are seeking. But a credible scientist should use judgement and context to select references.

The rebuttal tries unsuccessfully to rebut the myriad of papers in the literature with results conflicting with their conclusions regarding d16HER2 and ER signaling or drug resistance. There is no point in going back-and-forth with this line of debate. The field is fraught with artifactual findings, conflicting results, and ambiguity. The current manuscript simply piles on top of this body of ambiguity.

I’m afraid my conclusions about this work remain as they were in the first review. This paper continues along an exhausted line of nearly three decades of a failed narrative to promote a role for d16HER2 in human cancer biology and therapeutics. This work continues the pursuit of an agenda rather than the thread of science. I think the conclusions of this work regarding the biology of d16HER2 in human cancers and its implications for the treatment of human cancers are unjustified.

Reviewer #4

(Remarks to the Author)
I co-reviewed this manuscript with one of the reviewers who provided the listed reports. This is part of the Nature Communications initiative to facilitate training in peer review and to provide appropriate recognition for Early Career Researchers who co-review manuscripts.

Version 2:

Reviewer comments:

Reviewer #1

(Remarks to the Author)
Overall concerns have been addressed. However, the new results involving SUZ12 appear too preliminary, as the current discussion does not fully align with established findings.

More specifically, SUZ12, a component of the PRC2 complex, is often linked to decreased ER expression. However, the discussion of SUZ12’s potential role remains unclear (line 529-39). Although increased methylation of the SUZ12 promoter was observed in luminal B and embryonic stem cells (line 535-39), ChiP-X enrichment analysis indicates higher activity in D16 tumors. This ChiP-X finding appears to contradict the suggested methylation state. The paragraph can be clarified or the following sentences removed:

"This is evidenced by bulk RNA-sequencing of Δ16IC tumors which shows enrichment of SUZ12 in addition to ESR1 based on ChIP-X enrichment analysis (Supplementary Fig. 2B). Interestingly, higher levels of SUZ12 methylation have been reported within Luminal B tumors57. In embryonic stem cells, more differentiated CD24+ cell populations exhibited increased DNA methylation at promoters of SUZ12 target genes, suggesting that epigenetic regulation by SUZ12 is involved in maintaining luminal cell identity although this has yet to be studied in the context of HER2Δ16 expressing tumors".

Reviewer #2

(Remarks to the Author)

The authors have responses to each of the additional specific points of the reviewers and included additional experiments to strengthen the manuscript. The revised manuscript is improved and the limitations are acknowledged.

Reviewer #4

(Remarks to the Author)
I co-reviewed this manuscript with one of the reviewers who provided the listed reports. This is part of the Nature Communications initiative to facilitate training in peer review and to provide appropriate recognition for Early Career Researchers who co-review manuscripts.

Open Access This Peer Review File is licensed under a Creative Commons Attribution 4.0 International License, which permits use, sharing, adaptation, distribution and reproduction in any medium or format, as long as you give appropriate credit to the original author(s) and the source, provide a link to the Creative Commons license, and indicate if changes were made.
In cases where reviewers are anonymous, credit should be given to 'Anonymous Referee' and the source.
The images or other third party material in this Peer Review File are included in the article's Creative Commons license, unless indicated otherwise in a credit line to the material. If material is not included in the article's Creative Commons license and your intended use is not permitted by statutory regulation or exceeds the permitted use, you will need to obtain permission directly from the copyright holder.
To view a copy of this license, visit https://creativecommons.org/licenses/by/4.0/

William J. Muller, Ph.D.
Professor of Biochemistry
Rosalind and Morris Goodman Cancer Centre
McGill University
1160 Pine Avenue West
Montreal, QC, Canada H3A 1A3

November 21st, 2025

Dear *Nature Communications* Reviewers,

We sincerely thank all Reviewers for your insightful comments and feedback on our manuscript entitled '**HER2Δ16 directs luminal cell identity and estrogen receptor signaling in HER2+ breast cancer**'. Here, to address your concerns, we give a point-by-point clarification on each of the Reviewer comments and outline how we have addressed each concern in our revised manuscript to strengthen the scientific merit of our study. Our comment addresses below are in red.

Comments from Reviewers:

Reviewer #1 (Remarks to the Author):

This manuscript is a research article from Hailey Proud et al., which investigates the role of HER2 isoforms (more specifically HER2Δ16) in driving luminal cell identity, estrogen receptor (ER) expression, signaling, and endocrine response in breast cancer. The primary model adopted to carry out this investigation was based on syngeneic MMTV-HER2 genetically-modified mouse models (Control (full length) and HER2Δ16). The results suggest that HER2Δ16 drives the early onset of primary tumor while increasing lung metastasis incidence. These results corroborate previous studies as summarized in the following article: (PMID: 31261614).

However, a more surprising observation of this manuscript derives from the phenotypic and molecular analyses demonstrating increased luminal phenotype of HER2Δ16 tumors. Overall, the manuscript is well-written but has various limitations that need to be addressed.

General comments:

1- The association of HER2Δ16 with ER signaling has been well-established in the manuscript. While cross-talk between the two factors is well-accepted, the mechanisms contributing to ER induction remain elusive in the study. Moreover, the co-expression of full-length HER2 and HER2Δ16 seems determinant in the ER phenotypic outcome.

We would like to thank Reviewer #1 for their comments and agree that the mechanism of crosstalk between HER2Δ16 and ER signaling requires further exploration. We have amended the current manuscript to further evaluate the contribution of MAPK-signaling as a direct downstream event of HER2Δ16 activity which can contribute to both the luminal and EMT phenotype of HER2+ cancer cells. Across our HER2Δ16 transgenic model systems (Δ16IC, FE16$^{flx/flx}$) we observed robust phosphorylation of MAPK, which has been extensively linked to ER-activity through sustaining non-canonical ER pathway activation (**Fig. 7A-B**)[1, 2]. In patient datasets, high expression of MAPK predicts poor relapse-free survival when treated with endocrine therapy (**Fig. 7C**) and trends towards worse outcomes in those treated with trastuzumab (**Fig. 7D**). Specifically, patients stratified as HER2+/ER+ typically have worse overall survival when MAPK expression

McGill

William J. Muller, Ph.D.

Professor of Biochemistry
Rosalind and Morris Goodman Cancer Centre
McGill University
1160 Pine Avenue West
Montreal, QC, Canada H3A 1A3

is increased, and the ratio of MAPK expression relative to ESR1 levels is a strong prognostic indicator for response to tamoxifen (**Fig. 7E-F**). Within our human *in vitro* models, administration of the selective MEK inhibitor PD98059, significantly hinders cell proliferation and synergizes with tamoxifen, something not observed in full-length HER2 controls (**Fig. 7G**). We believe this strengthens the existing manuscript by providing one mechanism by which HER2Δ16 tumors may sustain ER-pathway activation, while reconciling the existing literature which shows that HER2Δ16 tumors may display resistance to tamoxifen in certain cellular contexts[3].

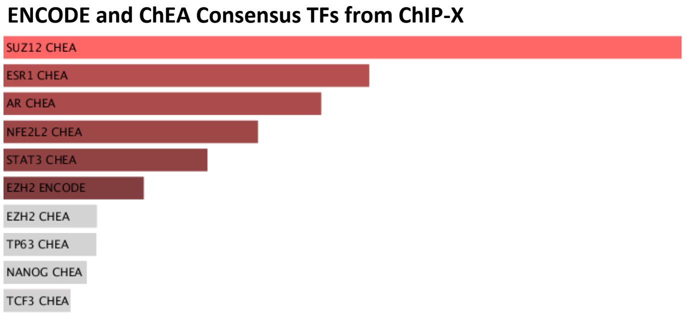

We do recognize that MAPK alone is not responsible for the full luminal phenotype present in HER2Δ16 tumors as we report multiple instances of transcriptional upregulation of ER which is not explained by MAPK activity. Although we believe it is outside the scope of the current manuscript, we do observe increased epigenetic signatures from core proteins within the PRC2 complex (such as SUZ12 and EZH2) which may be driving the different transcriptional programs observed in Δ16IC relative to EIC tumors. Currently, we have a manuscript in revision which further explores the precise mechanism of EZH2 regulation of luminal versus mesenchymal cell identity in wildtype-HER2 tumors.

2- Many results presented in the manuscript are confirmatory (e.g., Fig. 1). This includes experiments related to HER2 targeting.

Although we agree that the experiments in Figure 1 are confirmatory of the aggressive and pro-metastatic phenotype driven by HER2Δ16 tumors, the resulting finding that these tumors display a luminal-differentiated phenotype offers a novel contribution to the field. Furthermore, the development of effective transgenic systems for modeling ER+/HER2+ breast cancer is of high relevance, as few immunocompetent models can recapitulate this molecular subtype[4, 5]. We believe that this, combined with our findings that targeting ER in combination with HER2 or MAPK inhibition is effective in treating both murine and human HER2Δ16 expressing cells offers a novel contribution worthy of publishing (Fig. 2, 4, 6).

3- Based on immunofluorescence images, it is clear that both control and HER2Δ16 tumors are expressing ER, despite the pronounced increase in the latter. Therefore, if pathological analyses were to be performed, both tumors would likely be classified as HER2+/ER+. While the manuscript mainly investigates the increased ER signaling, it remains intriguing how HER2Δ16 promotes both the luminal and EMT phenotype of cancer cells. Moreover, a dominant effect seems to occur in conditions where both full-length and HER2Δ16 are expressed.

William J. Muller, Ph.D.

Professor of Biochemistry
Rosalind and Morris Goodman Cancer Centre
McGill University
1160 Pine Avenue West
Montreal, QC, Canada H3A 1A3

Although both EIC and Δ16IC tumors display *ESR1* transcript within the primary lesions, the addition of ER-protein staining within the manuscript reveals that the EIC model exhibits minimal ERα protein expression with <1% ER+ cells within the primary tumor (**Fig. 2C**). In contrast, Δ16IC tumors retain robust ER expression, up to 6% of the total HER2+ cell population, demonstrating that while EIC tumors remain stratified within the HER2+ molecular subtype the Δ16IC model more closely resembles an ER+/HER2+ tumor type (**Fig. 2C**).

We agree that the dual nature of Δ16IC tumors in promoting both luminal and EMT phenotypes is intriguing. However, we would also like to take this opportunity to emphasize that existing research has been performed on the mechanisms of EMT program activation in HER2Δ16 tumors. These studies identified that HER2Δ16 expression couples with several downstream oncogenic signal transduction pathways which increase expression of focal adhesion kinase (FAK), Phosphatidylinositol-3-Kinase/Protein Kinase B (PI3K/AKT), Mitogen-Activated Protein Kinase (MAPK) and Proto-oncogene tyrosine-protein kinase SRC (SRC), all of which have been extensively implicated in cancer cell migration, and induction of the EMT[6-9]. Furthermore, our endogenous model of HER2Δ16 (MMTV-Cre FE16$^{flx/flx}$) which places expression of this variant under the HER2 promoter within the mammary epithelium gives rise to ER+/HER2+ tumors which more closely recapitulate the normal physiological role of HER2Δ16 and demonstrate less of an EMT-phenotype compared to that observed in the Δ16IC overexpression model. For these reasons our focus was to explore the role of ER and epithelial cell expression within an intrinsically immunocompetent model, and the role it may have in promoting cancer cell growth and therapeutic resistance in these tumors. We have amended our discussion to elaborate on the dual luminal and EMT phenotype observed in HER2Δ16 tumors and the molecular mechanisms we believe are underlying this phenotype.

Specific comments:
4- Fig.1 confirms the association of d16 with early onset and worse survival outcomes.

We agree with the above comment that our research, combined with the existing literature establishes that HER2Δ16 associates with early onset and worse survival outcomes[6, 10-12].

5- Fig 2G Tamoxifen treatment reduces HERD16-expressing cell growth to the level of HER2. Therefore, using this as a biomarker for endocrine response remains limited.

Although in Figure 2G tamoxifen was unable to bring the proliferative capacity of NMuMG/HER2Δ16 cells below that of the wildtype-HER2 control, in several other human cell lines tamoxifen treatment was sufficient to hinder tumor growth equivalent to the effects of

McGill

William J. Muller, Ph.D.

Professor of Biochemistry
Rosalind and Morris Goodman Cancer Centre
McGill University
1160 Pine Avenue West
Montreal, QC, Canada H3A 1A3

lapatinib treatment in wildtype-HER2 or parental cells (Fig. 6C, 6E). Additionally, tamoxifen offered an additive effect when combined with either the MEK inhibitor PD98059 or lapatinib (Fig. 6C, 7G), arguing that in HER2Δ16-expressing cells the addition of tamoxifen in combination with inhibitors targeting HER2 or its downstream signaling axis is a more effective treatment approach.

6- Fig.2a: Provide higher resolution images to help determine the localization of ER in tumors

We recognize that the localization of ER in HER2Δ16 tumors was previously unclear in our model systems. We have amended Figure 2 to include a new panel showing immunofluorescent protein staining of EIC and Δ16IC tumors which distinctly shows nuclear ER localization (**Fig 2C**).

7- Fig.4: Immune cells appear to increase in tamoxifen-treated mice. However, to ascertain that the phenotypic change exerts anti-tumor responses, functional assessment needs to be performed.

We agree that the proposed manuscript would benefit from functional assessment of immune cell populations increased in tamoxifen-treated mice. We would like to propose additional characterization of tamoxifen-treated tumors to determine the activation of both T-cell and macrophage populations through immunofluorescent staining and RNA-ISH using the markers IL-6, Granzyme B and PD-1, which we would be happy to submit with the final version of the manuscript.

8 - Figure 7 proposes an estrogen-independent mechanism or ER signaling activation in HER2Δ16 tumors. However, to what extent is the HER2Δ16-mediated ER signaling relevant in estrogen-independent conditions (e.g., menopause)? This can be tested in vivo and validated in vitro. Additionally, it is unclear how HER2Δ16 promotes ER expression. Based on qPCR experiments, ER may be transcriptionally regulated in HER2Δ16 tumors. Exploring the mechanism of activation will bring mechanistic novelty to this manuscript.

Regarding the role of HER2Δ16 in promoting ER expression, this concern was addressed in general comment #1, please see above. Although evaluating the relevance of HER2Δ16-mediated signaling in estrogen-independent conditions is of interest for future study, repetition of *in vivo* experiments in animals lacking estrogen stimulus (such as in ovariectomized mice) lies beyond the current framework of the project.

9- Is the mechanism of ER activation maintained in heterozygous conditions? If yes, this needs to be included in the summary figure (Fig.7c). Moreover, how does the author reconcile these findings with the negative crosstalk often observed between ER and Her2?

McGill

William J. Muller, Ph.D.

Professor of Biochemistry
Rosalind and Morris Goodman Cancer Centre
McGill University
1160 Pine Avenue West
Montreal, QC, Canada H3A 1A3

Additional characterization of MMTV-Cre FE16$^{wt/flx}$ tumors revealed similar expression of ER and downstream transcriptional targets while *in vitro* experiments in human cell lines show that ER-activity is maintained when both isoforms are co-expressed, signifying that ER-activation is maintained in heterozygous conditions (Fig. 6, Supplementary Fig. 4). However, due to the abundant literature which dictates that HER2Δ16 preferentially forms stable homodimers, we believe that the current mechanism of action included in summary Figure 7C is reflective of both homozygous and heterozygous conditions where HER2Δ16 is expressed[7, 8, 13].

While there is a negative correlation between ER and HER2 observed in breast cancer cases, patients co-expressing both receptors still account for up to 10% of cases[14]. Furthermore, based on our results HER2Δ16 alone can initiate an ER-transcriptional program which was not observed in full-length HER2 expressing cells. As HER2Δ16 makes up a fraction of HER2 transcript the effects of HER2Δ16 expression may not be properly reflected in HER2+ patient datasets, nor would it be detected by standard histological subtyping. While it would be ideal to profile patient ER+/HER2+ tumor samples for the association of HER2Δ16 and ER it is unfortunately beyond the feasibility of the current state of the laboratory but warrants future investigation.

10- Erbb4 increased in HER2D16 tumors, suggesting a possible impact on ER signaling. How do you uncouple this from the direct impact of HER2D16 on ER signaling and increased luminal phenotype?

We agree with Reviewer #1 that elevated expression of ErbB4 may impact ER signaling within our model systems. To address this concern, we would suggest profiling both our murine tumors and human cell lines for ErbB4 expression. While this is in progress, preliminary data from RNA-sequencing of EIC and EIC/FE16$^{flx/flx}$ tumors shows that there is no difference in ErbB4 expression suggesting this phenotype may be limited to the Δ16IC model system and not reflected across the multiple HER2Δ16 models utilized within this study.

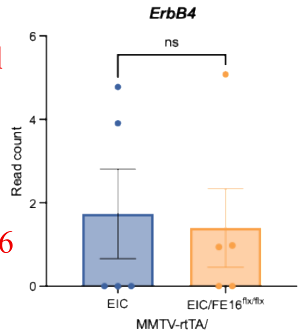

Reviewer #2 (Remarks to the Author):

In this manuscript the authors show that HER2Δ16 expression in MMTV-driven GEMM and knock-in mouse mammary tumor models promotes a shift to luminal differentiation states and shifts tumors toward ER-driven signaling, making the cells sensitive to endocrine therapy. They also show that this shift is also observed in human HER2+ breast cancer cell lines upon resistance to lapatinib, suggesting a compensatory reliance on ER signaling. Mouse mammary tumor models are generally ER negative and not dependent on estrogen signaling. Demonstrating the ER+ phenotype and ER dependency of this model would make it potentially useful for studies of ER+ breast cancer. Thus, the manuscript could be a valuable addition to the field.

William J. Muller, Ph.D.

Professor of Biochemistry
Rosalind and Morris Goodman Cancer Centre
McGill University
1160 Pine Avenue West
Montreal, QC, Canada H3A 1A3

However, the ER positivity and dependency of the model is not conclusively proven.

Major comments:
1. The authors show ER expression only by Esr1 RNA ISH, not at the protein level. They have to perform ER immunostaining on the tumors and/or immunoblot analyses.

We agree with Reviewer #2 that ER expression by RNA ISH is not sufficient to derive conclusions regarding ER-protein expression. We have since optimized staining conditions to detect murine ER within our transgenic models and have updated the manuscript to include quantification of ER protein within EIC, Δ16IC and FE16 models (**Fig. 2C,** Supplementary Fig. 4A).

2. Endocrine therapy can inhibit mammary tumor growth even in ER-negative models through the microenvironment, especially immune cells. Thus, to prove that the effect of tamoxifen is through targeting ER in the tumor cells the authors have to delete Esr1 in the model and also use ER degraders – tamoxifen is not a full ER antagonist.

Ongoing experiments within our laboratory have begun crossing our existing HER2Δ16 model systems with a conditional ESR1 knock-out model, allowing for ESR1 deletion specifically within the mammary epithelium. Unfortunately, as these transgenic crosses take several months to accomplish, they are outside the current scope of the manuscript. However, the effective anti-proliferative capacity of tamoxifen treatment *in vitro* within our murine and human cell lines would suggest that the observed delay in tumor growth *in vivo* is independent of the immune-modulating effects of tamoxifen. Additionally, treatment of MMTV-Cre FE16$^{flx/flx}$ tumor derived cell lines with the ER degrader fulvestrant, shows a significant delay in cell proliferation, similar to what was observed *in vivo* upon tamoxifen treatment.

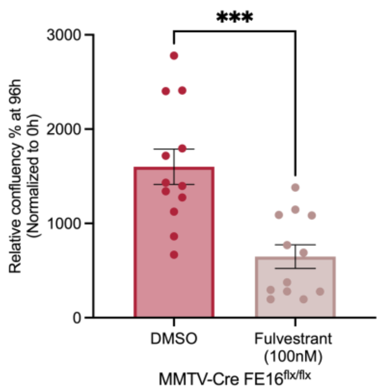

3. A characterization of the FE16 wt/fx model should be done more thoroughly. The authors specify that "heterozygous deletion of exon 16 in this model is not sufficient to promote mammary tumorigenesis without additional pro-oncogenic mutations" (line 370), but some mice developed tumors with the same latency as the fx/fx. What other mutations does these tumors have then? What is the HER2 expression and amplification status in the FE16 wt/fx tumors. What proportion of ErBB2 wt and Δ16 is there? If HER2 Δ16 expression is intermediate compared to the fx/fx, what is the ER, CK8, EpCAM expression?

We apologize for the oversimplification of the heterozygous FE16$^{wt/flx}$ phenotype and agree with Reviewer #2 that this requires further investigation. Accordingly, we have amended the manuscript to include characterization of these tumors. Within the arising FE16$^{wt/flx}$ tumors, we observe similar

McGill

William J. Muller, Ph.D.

Professor of Biochemistry
Rosalind and Morris Goodman Cancer Centre
McGill University
1160 Pine Avenue West
Montreal, QC, Canada H3A 1A3

elevation of ER expression as well as the epithelial markers CK8 and EpCAM (Supplementary Fig. 3A-B). Furthermore, we discerned no differences in downstream ER-transcriptional activity or the relative expression of *ErbB2Δ16* between FE16$^{wt/flx}$ and FE16$^{flx/flx}$ endpoint tumors (Supplementary Fig. 3C). Overall, this suggests that heterozygous expression of HER2Δ16 is preferentially selected and sufficient to drive ER+ tumorigenesis within this model, although co-expression of both isoforms decreased the frequency of tumor formation. Interestingly, FE16$^{wt/flx}$ tumors share similar amplification of the *ErbB2* gene, further suggesting this is a driving event supporting tumor initiation within this model (Supplementary Fig. 3D). This data, coupled with the degree of ER activity and expression of epithelial cell markers shared by both models, suggests that in instances of co-expression of both HER2 isoforms, HER2Δ16 is determinant in phenotypic outcome and that heterozygous deletion of HER2Δ16 can promote mammary tumorigenesis. As such we have updated the manuscript to reflect these new findings.

4. In the model with co-expression of full-length HER2 and ErbB2Δ16, what is the expression levels of both HER2 full-length and Δ16? Does it resemble what is present in the human cases (HER2Δ16-positive breast cancer cases, HER2Δ16 makes up 4-10% of total HER2 transcript).

Our novel EIC/FE16$^{flx/flx}$ model which co-expresses both full-length and ErbB2Δ16 splice isoforms maintains lower expression of HER2Δ16 relative to our human doxycycline-inducible model (Δ16IC). However, the levels of HER2Δ16 are still elevated relative to what is present in human cases. For this reason, we believe our human *in vitro* cell lines which can be characterized as HER2Δ16 low/high and fall within the clinically relevant 4-10% range are a better model system when assessing the contribution of either isoform rather than the transgenic models which represent an exaggerated system more attuned to studying the biochemical pathways preferentially regulated by HER2Δ16 expression (Supplementary Fig. 7A).

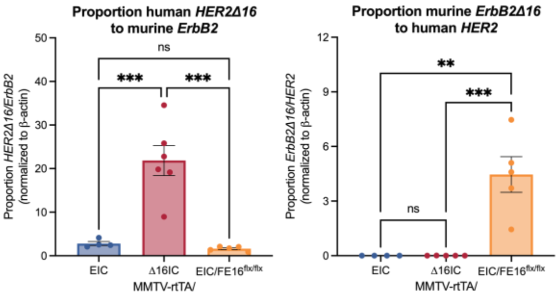

5. For the lapatinib resistant model, is lapatinib selecting for cells with a prior higher expression of HER2Δ16? Is there a population already expressing higher levels of HER2Δ16 ER in these cells?

Based on our characterization of the parental HER2+ human cell lines we do believe it is possible that lapatinib selected for cells with a prior higher expression of HER2Δ16 within the UACC812 cell line. This is because the UACC812 parental line maintained an intermediate level of HER2Δ16 transcript relative to other HER2+ cell lines, despite having a lower ratio of HER2Δ16 expression relative to the full-length isoform (Supplementary Fig. 7A). However, our revised manuscript incorporates a second lapatinib resistant cell line (SK-BR-3), where we believe it is unlikely that

McGill

William J. Muller, Ph.D.

Professor of Biochemistry
Rosalind and Morris Goodman Cancer Centre
McGill University
1160 Pine Avenue West
Montreal, QC, Canada H3A 1A3

lapatinib selected for cells with high HER2Δ16 expression. This is because SK-BR-3 cells maintain consistently lower total HER2Δ16 and relative HER2Δ16/HER2 levels compared to other HER2+ cell lines (Supplementary Fig. 7A). Additionally, lapatinib resistant cells derived from this model exhibit lower HER2Δ16 expression relative to HER2Δ16 high cell lines (SUM225CWN, ZR-7530) and the lapatinib resistant UACC812 line therefore, it is unlikely SK-BR-3 parental cells maintain high enough HER2Δ16 transcript levels to prompt immediate selection upon lapatinib treatment rather than promoting alternative HER2-splicing upon acquiring resistance (Supplementary Fig. 8B).

6. Fig. 7A, there is no total ER blotted. The levels of phospho-ER should be normalized to it. As its expression is higher in the HER2Δ16 tumors, the ratio may not be different, and the conclusion of an increased phosphorylation cannot be made. Same thing for all phosphorylation assessment, the quantification should be phosphorylated over total.

We agree with Reviewer #2 that normalizing phosphorylated relative to total protein expression would be a more accurate method of quantifying protein phosphorylation within our models. We have amended our figure panels accordingly for all immunoblots (**Fig. 3E, Fig. 7A**, Supplementary Fig. 5A). Unfortunately, we are unable to quantify phosphorylated ER relative to total ER within our samples as we are limited by a lack of commercially available antibody to specifically detect murine ER levels within bulk tumor lysates by immunoblotting. Similarly, there is no murine pER^{S118} antibody for immunofluorescent staining to complement total ER levels within our models. However, we can propose additional validation utilizing a phosphorylated-ER ELISA assay in our human cell lines to quantify the relative levels of pER^{S118} in our HER2Δ16 low and high cell lines to further assess ER phosphorylation.

7. In cells in vitro (NMuMG) and in human resistant models, is there an increase of ERK signaling? Is this signaling important for the increase of ER expression, rather than its phosphorylation? If MAPK pathway is inhibited, would ER expression or phosphorylation change?

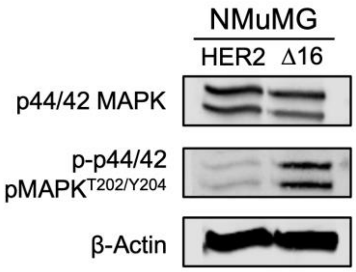

In our NMuMG/HER2Δ16 cell lines we do detect elevated levels of ERK phosphorylation (T202/Y204) relative to total ERK expression. In publicly available human datasets, the expression of MAPK, specifically p44 MAPK (MAPK1), corresponds with worse 5-year relapse free survival in breast cancer patients treated with endocrine therapy (**Fig. 7C**). Within ER+/HER2+ patient tumors, high MAPK1 expression significantly correlates with worse overall survival (**Fig. 7E**). This is particularly evident in cases when MAPK1 expression is high relative to ESR1, where tamoxifen treated patients demonstrate significantly worse relapse-free survival, which may be attributed to the estrogen-independent ER-signaling driven by MAPK1 phosphorylation, which is

Professor of Biochemistry
Rosalind and Morris Goodman Cancer Centre
McGill University
1160 Pine Avenue West
Montreal, QC, Canada H3A 1A3

known to confer tamoxifen resistance (**Fig. 7F)**[2, 15]. Upon treatment with the selective MEK inhibitor, PD98059, we show that NMuMG/HER2Δ16 cell lines exhibit decreased proliferation relative to DMSO treated controls and that there is an additive effect when combining MAPK pathway inhibition with tamoxifen, suggesting this combination is effective in reducing HER2Δ16 cell proliferation (**Fig 7G**).

Minor comments:

1. M1 and M2 macrophage nomenclature is outdated, it would be better to refer as pro- and anti-tumoral or indicate the marker expression.

We apologize for the use of outdated nomenclature and agree with Reviewer #2 that these populations would more accurately be described as pro- and anti-tumor macrophages. We have modified the manuscript to reflect this change.

2. Throughout the paper, the microscopy figures would benefit from including a zoomed inset (especially where it is required to for the conclusions like in Fig.1J, Fig. 3D).

We are happy to include a zoomed inset where applicable.

3. Throughout the paper, it will be good to harmonize font size between panels. Some have text is too small to read properly (ex. qPCR bar graph).

We are happy to adjust the labelling between panels to harmonize font size.

4. Line 329 – reference to Supplementary Fig. 2A should be Supplementary Fig. 2B

We thank Reviewer #2 for pointing out this discrepancy and have amended the manuscript accordingly.

Reviewer #3 (Remarks to the Author):

Summary

In this paper the authors study transgenic models of d16HER2-induced tumorigenesis and report that it has transcriptional differences with the wtHER2-induced tumors. Most importantly they report that d16HER2-induced tumors have active estrogen receptor signaling and take this further into compound transgenic models and human tumor cells lines to suggest that the d16HER2 transcript of HER2 underlies estrogen receptor expression and signaling and this underlies resistance to HER2 inhibitors and sensitization to tamoxifen.

Professor of Biochemistry
Rosalind and Morris Goodman Cancer Centre
McGill University
1160 Pine Avenue West
Montreal, QC, Canada H3A 1A3

General criticism:
There is a lot of work done in this paper and a lot of time spent generating and studying these mice. I don't have specific criticisms of the methodology and techniques. The experiments are well done and the data looks good. I am not going to offer criticisms of specific experiments and data items because I don't think it's relevant. My criticism is about the background, context, interpretation, relevance, and conclusions and lack of scientific objectivity. This paper continues along an exhausted line of nearly three decades of a failed narrative to promote a role for d16HER2 in human cancer biology and therapeutics. The problem is that these are experiments that produce experimental results, many of which conflict with eachother, many that conflict with clinical evidence, and the reality is that the experimental work is disconnected from and not relevant to human cancer biology and therapeutics. This is merely experimental science for the purpose of experimental science. I think the conclusions of this work regarding the biology of d16HER2 in human cancers and its implications for the treatment of human cancers are unjustified.

We would like to thank Reviewer #3 for taking the time to review the manuscript and discuss the surrounding literature involving HER2Δ16. Overall, while we agree there is much debate in the field centering around the role of HER2Δ16 in dictating resistance to HER2-targeted therapy and the implications for human cancers, it is incorrect to state that the HER2Δ16 splice variant has no relevance or impact to human disease. In fact, HER2Δ16 has been extensively studied across multiple solid cancers not limited to breast. Our group has more recently established that HER2Δ16 expression is present in 42% of naïve HER2+ primary breast tumors after analyzing patient samples from the FinXX clinical trial as well as being noted in 14 patients classified as triple-negative at the time of diagnosis[10]. Furthermore, survival analysis of HER2Δ16- positive and -negative patients revealed that HER2Δ16 expression associated with reduced recurrence-free survival when no adjuvant trastuzumab was given[10]. This agrees with the previous literature demonstrating detection of HER2Δ16 in 52% of HER2-positive breast tumors from an additional cohort of primary invasive breast cancer[6]. Again, HER2Δ16 is a prognostic indicator of poor outcome within this patient subset, 89% of patients with HER2Δ16-positive tumors presented with positive lymph nodes compared to only 12% of the HER2Δ16-negative group[6]. This clinically relevant finding is also supported by *in vitro* models of HER2+ human cancer, including our proposed manuscript, where HER2Δ16 transcript falls between 2-10% of total HER2 transcript across human cell lines from all breast cancer subtypes[16]. Total levels of HER2Δ16 are enriched within HER2+ cell lines, with the highest HER2Δ16 expressers from these established cell lines coinciding with our current findings within the manuscript (Supplementary Fig. 7)[16].

Moreover, there is further evidence for a role of HER2Δ16 in disease aggressiveness and poor prognostic outcome in other solid cancers. In non-small cell lung cancer (NSCLC) previous studies identified a clinical case demonstrating increasing allelic fractions of HER2Δ16 after treatment with Osimertinib, to which the patient was resistant[12]. This ability of HER2Δ16 to confer

Professor of Biochemistry
Rosalind and Morris Goodman Cancer Centre
McGill University
1160 Pine Avenue West
Montreal, QC, Canada H3A 1A3

Osimertinib resistance was confirmed using HER2Δ16-expressing NSCLC cell lines[12]. Further evidence for the role of HER2Δ16 in NSCLC is provided by our previous publication establishing that 4% of all NSCLC cases available through The Cancer Genome Atlas display enrichment of HER2Δ16[17]. Importantly, most cancer cases harboring HER2 splicing mutations contained relatively few mutations in other known oncogenes. Strikingly, missense alterations in splicing donor/acceptor sites across HER2 were almost exclusively confined to regions flanking exon 16, suggesting that HER2Δ16 is the predominant driver mutation within this cancer context[17]. There is also evidence for the clinical significance of HER2Δ16 in gastric cancer, where an examination of 110 patients with either locally advanced or metastatic HER2+ gastric cancer revealed that 48% of samples display elevated HER2Δ16 levels[11]. Overall survival of HER2Δ16-expressing patients upon treatment of trastuzumab was significantly reduced compared to HER2Δ16-negative patients and characterized by poor CD3+ T-cell infiltration and strong intratumoral expression of programmed death 1 and programmed death ligand 1[11]. This is supported by an additional case study demonstrating trastuzumab-pertuzumab resistance within HER2Δ16-positive colorectal cancer[18]. The observation that these tumors display reduced immune infiltration is reaffirmed by our own studies using our MMTV-rtTA Δ16IC model where we established that HER2Δ16 promotes an immune-cold microenvironment in breast cancer[10]. Collectively, this body of work demonstrates that there are strong clinical data and *in vitro* human models supporting HER2Δ16 expression and poor prognosis both within breast cancer and other solid tumor types, warranting its further study.

d16HER2 narrative:
The d16HER2 splice variant has been the subject of nearly three decades of studies. Many papers have now shown that d16HER2 is a more potent oncogene than wtHER2. There is no doubt about this experimental finding. The problem is that this experimental finding is not relevant to human cancer biology because these artifactual experimental models do not reproduce the human disease. The transgenic mouse model of d16HER2 mammary tumorigenesis has previously been published and develops tumors with accelerated kinetics compared to wtHER2 (PMID 21559085)(PMID 34131071)(PMID 27157621). So this is already well established. But while these models tell us about the high oncogenic potency of d16HER2, they do not inform us about human cancer biology, because the human cancers do not express d16HER2 at these high levels and in isolation. In fact this current paper by Proud et al does provide an important contribution, and that is the generation of a mouse transgenic model that uses Cre-lox to convert the endogenous Neu gene to an ex16-deleted version in mammary tissue, and this turns out NOT to be tumorigenic. This is despite the spin put on it here that in the homozygous genotype, and after several years of age, a very small subset of mice develop tumors (Figure 3). And these small subsets again have developed amplification and overexpression of d16HER2 and no longer are representative of human tumors which have low levels of d16HER2. Their compound genetic model of wtHER2 overexpression and d16HER2 knock-in shows that the co-expression of d16HER2 actually retards tumorigenesis, also arguing against a driver or accelerator role (Suppl

McGill

William J. Muller, Ph.D.

Professor of Biochemistry
Rosalind and Morris Goodman Cancer Centre
McGill University
1160 Pine Avenue West
Montreal, QC, Canada H3A 1A3

Fig 4). The important contribution of these newer genetic models is that it confirms the artifactual nature of the d16HER2 transgene overexpression models and its irrelevance to human cancer. It shows that the low amount of d16HER2 in the HER2-amplified human cancers is not the tumorigenic driver, and as always suspected, the wtHER2 overexpression is the driver of the human disease. I commend the authors for performing this experiment, and I suggest the honest interpretation of the findings, rather than continued promotion of a false d16HER2 narrative.

We acknowledge that the previous models used to study the oncogenic potency of HER2Δ16 utilize overexpression of the transgene and are not reflective of the levels of HER2Δ16 observed clinically. However, the human cell lines utilized within our proposed manuscript directly reflect the levels of HER2Δ16 expressed clinically and show a similar ER-responsive phenotype which can be effectively targeted using a combination of both endocrine and HER2 antagonists. Additionally, we must disagree with Reviewer #3 as the endogenous deletion of exon-16 in murine ErbB2 is in fact tumorigenic. Mice both heterozygous and homozygous for deletion of HER2Δ16 do develop spontaneous mammary tumors with homozygous mice exhibiting hyperplasia at 8-months of age (Fig. 3, Supplementary Fig 4.). Although we recognize that it takes several months for these tumors to develop within a subset of these mice, this aligns with the multitude of genetically-engineered ER+ mouse models widely accepted and utilized within the field[19]. In fact, the average latency of many of these models exceeds 400 days and display lower penetrance than that observed in our MMTV-Cre FE16$^{flx/flx}$ model[19]. Furthermore, these models cannot be used to study the ER-HER2 signaling axis.

To disregard this model due to its latency and/or penetrance would similarly dismiss many of the extensively published ER+ transgenic models utilized within the field. Within this model, HER2Δ16 does directly act as a tumorigenic driver as no tumor formation or equivalent amplification of HER2 is observed in control mice expressing only the endogenous Neu allele. Despite amplification or overexpression of HER2 remaining the hallmark of HER2+ breast cancer, these events significantly coincide with the increased prevalence of alternately spliced HER2 isoforms, such as HER2Δ16, whose prevalence within human cancers is clinically relevant and has been discussed above.

Conflicting literature and lack of clinical relevance:
This paper reports that d16HER2 expression is associated with induction of ESR1 and ER signaling. But in a clinical study of HER2 positive breast cancers, the higher co-expression of d16HER2 was associated with an ER negative subtype (PMID 19671734), and in another microarray gene expression study of a large number of breast tumors the expression of d16HER2 was associated with the ER negative state (PMID 23867476). In in vitro transfection studies of MCF7 cells, another group found that expression of d16HER2 reduces ER expression and induces endocrine resistance (PMID 20876285). The Muller group itself has previously done gene expression analysis of the d16HER2 transgenic mice and reported years ago that these

tumors have gene expression profiles that resemble the basal subtype of human breast cancers, not the luminal ER positive type (PMID 27157621). All these data contradict the suggestions of this current paper by Proud. This study reports that d16HER2 expression induces ER expression and confers sensitivity to tamoxifen. There are other experiments reported where d16HER2 expression seems to confer resistance to tamoxifen (PMID 20876285)(PMID 21172025). This study by Proud reports that d16HER2 expression, by virtue of inducing ER signaling, confers resistance to HER2 targeted therapy. But other studies have reported that d16HER2-induced mouse tumors were more sensitive to trastuzumab compared to wtHER2-driven tumors (PMID 28903354), and a clinical study found a positive correlation between d16HER2 and trastuzumab response (PMID 25164009). All these findings contradict the conclusions of this current paper by Proud. Now I am not saying that all these other papers are right, and this paper by Proud et al is wrong. There are other d16HER2 papers with more disparate results and other conclusions. The point is that this field of d16HER2 experimental science is fraught with experimental artifactual findings, many which are conflicting with eachother, but more importantly many and most of the findings are tied to that specific experimental model and technique and are detached from and have no relevance to the human disease. This current paper by Proud is just another experimental d16HER2 data set and perhaps should be placed in the public domain for others to see, but it simply piles on top of a heap of studies, internally conflicting, all of which are almost surely irrelevant to human HER2-driven breast cancers.

We strongly believe that HER2Δ16 is a physiologically relevant phenomena in human cancers which dictates therapeutic response, which we have thoroughly addressed above. We do recognize, however, that there is conflicting literature surrounding the ability of HER2Δ16 to confer sensitivity/resistance to HER2-targeted therapy[7, 20]. We would like to thank Reviewer #3 for bringing to our attention two independent studies stating that HER2Δ16 promotes trastuzumab sensitivity (PMID:20876285, PMID:21172025). However, we would like to point out that the human correlations between HER2Δ16 and response to trastuzumab were limited to one dataset (GSE22358) which reported a high degree (>50%) of TP53 mutations which was shown to impact therapeutic outcome, as well as being further stratifying HER2Δ16 tumors to specifically look at those with high pSrc expression[7]. Additionally, the transgenic models utilized by this group, among others is confounded by the coordinated expression of HER2Δ16 and fluorescent reporters such as luciferase, which are highly immunogenic and can lead to cellular changes within HER2Δ16 tumors which do not reflect physiological conditions[7, 8, 21]. We have shown evidence of this within our own research, with transgenic mice expressing HER2Δ16 in tandem with GFP displaying an accentuated EMT phenotype which we believe to be impacted by the expression of exogenous fluorescent reporters[16]. This is not to discredit the findings of these previous reports, but to instead emphasize the importance of using physiologically relevant models of HER2Δ16 expression in the context of assessing cellular identity within these models. In this regard, we believe that the MMTV-Cre FE16$^{flx/flx}$ model is of high utility to the scientific community, as it is the only model of HER2Δ16 expression which utilizes the endogenous murine

McGill

William J. Muller, Ph.D.

Professor of Biochemistry
Rosalind and Morris Goodman Cancer Centre
McGill University
1160 Pine Avenue West
Montreal, QC, Canada H3A 1A3

promoter. Furthermore, regardless of the role of HER2Δ16 expression, this model provides a preclinical platform to specifically look at ER+/HER2+ tumorigenesis within an immune-competent context, something which is currently limited within our field[4]. The relevance of this model extends beyond the role of HER2Δ16 in tumorigenesis and instead allows for the coordinated study of the HER2-ER signaling axis within aggressive HER2+ tumors. The pathways enriched by HER2Δ16 tumors such as the importance of MAPK and non-canonical ER signaling and the role of mRNA splice factors like SRSF3 in the development of ER+ tumors add additional insights to the field of HER2+/ER+ tumor biology, and are of clinical relevance based on the additional data provided (New Fig. 7B-G, New Supplementary Fig. 8).

Suggestion that mutations in mouse models are evidence of clinical relevance:
These and other authors often cite the development of mutations in the mouse transgenic models as a correlate of and as supporting evidence for the importance of the d16HER2 variant in the human cancers. Juxtamembrane region deletions do develop in Neu transgenic mice (PMID 7935422) and huHER2 transgenic mice (PMID 15073130) as referenced here (line 358). But they develop in only half the tumors. So the other half of these tumors do not have such mutations and are driven by wildtype Neu or HER2. While it is reasonable to suggest that such mutations confer an advantage to the transgene, it is incorrect to say that overexpression of the wildtype genes is insufficient and to suggest that mutations are required to drive tumorigenesis (line 358) since half of such tumors do not have these mutations. At this time, these mutations are a peculiarity of the mouse models and not known to be required for tumorigenesis and the human tumors do not have such mutations, and attribution of these mouse mutations as a correlate of d16HER2 variant in human tumors is unjustified, and the newer genetic models in this paper actually confirm that.

We agree with Reviewer #3 that in Neu and huHER2 transgenic mice although there is a selective advantage conferred by juxtamembrane region deletions which promotes tumorigenesis there are also cases in which tumors develop independent of these mutations. As such, we have amended our manuscript to avoid any overinterpretation of this finding (line 402). However, we disagree that these mutations are only a peculiarity of the mouse models as mutations within the juxtamembrane and other regions of HER2 have been definitively shown within human tumors. A previous study, analyzing the HER2 mutational landscape of 1184 patients with invasive breast cancer detected HER2 mutations in 8.9% of patients, with 4.3% of these mutations falling directly within the juxtamembrane domains[22]. Multivariate Cox regression analysis indicated that patients with HER2 mutations had a shorter progression-free survival (PFS) than HER2 wild-type patients upon first-line treatment with trastuzumab [22]. This reaffirms that indeed while HER2 mutations do not occur in all HER2+ cancer cases, they do make up a significant proportion of diagnosed cases and are implicated in therapeutic response.

McGill

William J. Muller, Ph.D.

Professor of Biochemistry
Rosalind and Morris Goodman Cancer Centre
McGill University
1160 Pine Avenue West
Montreal, QC, Canada H3A 1A3

d16 is HER2 baggage:

The reality is that the HER2 gene is amplified and massively overexpressed in cancers. Along with such massive overexpression comes a lot of baggage; ie many events that ordinarily are low level events are also increased in parallel. This includes increased expression of alternative isoforms and truncation products such as d16HER2 and p95HER2, it includes promiscuous interaction partners not normally seen at low expression levels, it includes abnormal localization events, and it includes abnormal protein processing, glycosylation and other modifications. All these events are consequences of massive overexpression of this gene and protein product. Many papers have been published about all these events that are much more pronounced and better detected in HER2-amplified cancers and many have suggested that these associated events underly the tumorigenic attributes of this oncogene. But all this body of work has been speculative, and an essential role for any of these "baggage" events has never been demonstrated, and it remains uncontested that overexpression of wtHER2 is sufficient for tumorigenesis and wtHER2 remains the principle form of this target that is relevant for therapeutics.

We do not disagree with Reviewer #3 that the HER2 gene is amplified across human cancers which coincides with genetic events (alternative isoforms, truncations, etc.) which occur in parallel. However, to disregard these genetic events as HER2 baggage ignores the importance of transcript diversity and its role in promoting varied protein functions, localization and of high clinical relevance, binding properties. The relevance of alternative splice isoforms is further emphasized by a recent publication profiling a collection of 561 primary breast cancer patient samples using mass spectrometry to expand the catalog of known HER2 protein-coding isoforms from 13 to 90 distinct variants[23]. This paper highlights the complex landscape of HER2 isoforms, and the array of alternate transcripts which are not assessed in routine practice. Importantly, assessment of HER2 isoform expression in human cell cultures which are sensitive or resistant to trastuzumab/antibody-drug conjugates (T-DM1 or T-DXd), found that drug-resistant cells shift their expression towards splice isoforms lacking antibody-binding domains[23]. This emphasizes the crucial role of alternative splicing in directing therapeutic response and the relevance of considering splice isoform diversity to further advance precision-targeted HER2+ cancer therapies.

In summary, we would like to thank Reviewer #3 for taking the time to debate and discuss the work surrounding HER2Δ16 in human breast. We are in agreement that therapeutic targeting of wtHER2 remains the gold-standard in treating HER2+ breast cancer, but to argue it is the sole relevant target vastly undermines the heterogeneity of HER2+ tumors and overlooks the high rates of intrinsic and acquired resistance which develop clinically[24]. Although HER2Δ16 is not the sole factor at play, it represents one alternately spliced isoform of HER2 that affects the molecular landscape of HER2 tumors and can influence sensitivity/resistance to different standard of care therapies. Furthermore, it remains evident that there are few immunocompetent ER+/HER2+ mouse models which permit *in vivo* study of ER+/HER2+ disease. As HER2Δ16 represents a constitutively active HER2 isoform, it facilitates the study of breast tumors co-driven by both ER

McGill

William J. Muller, Ph.D.

Professor of Biochemistry
Rosalind and Morris Goodman Cancer Centre
McGill University
1160 Pine Avenue West
Montreal, QC, Canada H3A 1A3

and HER2. The applications of these models extend outside the scope of HER2Δ16 biology and offer a substantial contribution to the field, useful in studying other aspects of HER2+/ER+ tumorigenesis and meriting scientific publication.

Reviewer #4 (Remarks to the Author):

I co-reviewed this manuscript with one of the reviewers who provided the listed reports. This is part of the Nature Communications initiative to facilitate training in peer review and to provide appropriate recognition for Early Career Researchers who co-review manuscripts.

We would like to thank Reviewer #4 for taking the time to review our manuscript and for providing their feedback.

Once again, we would like to sincerely thank our Reviewers for their insightful comments and constructive feedback. We are confident that this strengthened, revised manuscript successfully addresses your concerns to ensure that we provide a satisfactory research article for the readership of *Nature Communications*.

We thank you for your consideration and look forward to hearing from you soon.

Yours sincerely,

Professor William J. Muller, Ph.D
Canadian Research Chair in Molecular Oncology
Professor, Department of Biochemistry, McGill University
Rosalind and Morris Goodman Cancer Institute

William J. Muller, Ph.D.

Professor of Biochemistry
Rosalind and Morris Goodman Cancer Centre
McGill University
1160 Pine Avenue West
Montreal, QC, Canada H3A 1A3

References

1 McGlynn LM, Tovey S, Bartlett JM, Doughty J, Cooke TG, Edwards J. Interactions between MAP kinase and oestrogen receptor in human breast cancer. *Eur J Cancer* 2013; 49: 1176-1186.

2 Qi X, Zhi H, Lepp A, Wang P, Huang J, Basir Z *et al*. p38gamma mitogen-activated protein kinase (MAPK) confers breast cancer hormone sensitivity by switching estrogen receptor (ER) signaling from classical to nonclassical pathway via stimulating ER phosphorylation and c-Jun transcription. *J Biol Chem* 2012; 287: 14681-14691.

3 Cittelly DM, Das PM, Salvo VA, Fonseca JP, Burow ME, Jones FE. Oncogenic HER2Delta16 suppresses miR-15a/16 and deregulates BCL-2 to promote endocrine resistance of breast tumors. *Carcinogenesis* 2010; 31: 2049-2057.

4 Ozdemir BC, Sflomos G, Brisken C. The challenges of modeling hormone receptor-positive breast cancer in mice. *Endocr Relat Cancer* 2018; 25: R319-R330.

5 Mohibi S, Mirza S, Band H, Band V. Mouse models of estrogen receptor-positive breast cancer. *J Carcinog* 2011; 10: 35.

6 Mitra D, Brumlik MJ, Okamgba SU, Zhu Y, Duplessis TT, Parvani JG *et al*. An oncogenic isoform of HER2 associated with locally disseminated breast cancer and trastuzumab resistance. *Mol Cancer Ther* 2009; 8: 2152-2162.

7 Castagnoli L, Iezzi M, Ghedini GC, Ciravolo V, Marzano G, Lamolinara A *et al*. Activated d16HER2 homodimers and SRC kinase mediate optimal efficacy for trastuzumab. *Cancer Res* 2014; 74: 6248-6259.

8 Marchini C, Gabrielli F, Iezzi M, Zenobi S, Montani M, Pietrella L *et al*. The human splice variant Delta16HER2 induces rapid tumor onset in a reporter transgenic mouse. *PLoS One* 2011; 6: e18727.

9 Alajati A, Sausgruber N, Aceto N, Duss S, Sarret S, Voshol H *et al*. Mammary tumor formation and metastasis evoked by a HER2 splice variant. *Cancer Res* 2013; 73: 5320-5327.

10 Attalla SS, Boucher J, Proud H, Taifour T, Zuo D, Sanguin-Gendreau V *et al*. HER2Delta16 Engages ENPP1 to Promote an Immune-Cold Microenvironment in Breast Cancer. *Cancer Immunol Res* 2023; 11: 1184-1202.

11 Wang S, Zhao Y, Song Y, Qiao G, Di Y, Zhao J *et al*. ERBB2D16 Expression in HER2 Positive Gastric Cancer Is Associated With Resistance to Trastuzumab. *Front Oncol* 2022; 12: 855308.

12 Hsu CC, Liao BC, Liao WY, Markovets A, Stetson D, Thress K *et al*. Exon 16-Skipping HER2 as a Novel Mechanism of Osimertinib Resistance in EGFR L858R/T790M-Positive Non-Small Cell Lung Cancer. *J Thorac Oncol* 2020; 15: 50-61.

13 Siegel PM, Ryan ED, Cardiff RD, Muller WJ. Elevated expression of activated forms of Neu/ErbB-2 and ErbB-3 are involved in the induction of mammary tumors in transgenic mice: implications for human breast cancer. *EMBO J* 1999; 18: 2149-2164.

McGill

William J. Muller, Ph.D.

Professor of Biochemistry
Rosalind and Morris Goodman Cancer Centre
McGill University
1160 Pine Avenue West
Montreal, QC, Canada H3A 1A3

14 Pegram M, Jackisch C, Johnston SRD. Estrogen/HER2 receptor crosstalk in breast cancer: combination therapies to improve outcomes for patients with hormone receptor-positive/HER2-positive breast cancer. *NPJ Breast Cancer* 2023; 9: 45.

15 Chen M, Cui YK, Huang WH, Man K, Zhang GJ. Phosphorylation of estrogen receptor alpha at serine 118 is correlated with breast cancer resistance to tamoxifen. *Oncol Lett* 2013; 6: 118-124.

16 Turpin J, Ling C, Crosby EJ, Hartman ZC, Simond AM, Chodosh LA *et al*. The ErbB2DeltaEx16 splice variant is a major oncogenic driver in breast cancer that promotes a pro-metastatic tumor microenvironment. *Oncogene* 2016; 35: 6053-6064.

17 Smith HW, Yang L, Ling C, Walsh A, Martinez VD, Boucher J *et al*. An ErbB2 splice variant lacking exon 16 drives lung carcinoma. *Proc Natl Acad Sci U S A* 2020; 117: 20139-20148.

18 Ghatak S, Lin K, Lou E, Nelson AC. Trastuzumab-Pertuzumab resistance in a case of HER2-del16 expressing colorectal cancer—a case report. *Precision Cancer Medicine* 2020; 3.

19 Dabydeen SA, Furth PA. Genetically engineered ERalpha-positive breast cancer mouse models. *Endocr Relat Cancer* 2014; 21: R195-208.

20 Palladini A, Nicoletti G, Lamolinara A, Dall'Ora M, Balboni T, Ianzano ML *et al*. HER2 isoforms co-expression differently tunes mammary tumor phenotypes affecting onset, vasculature and therapeutic response. *Oncotarget* 2017; 8: 54444-54458.

21 Khan KA, Qureshi AA, Xu P, Kuo HY, Schumacher TN, Michael IP *et al*. Immunological Tolerance to Luciferase and Fluorescent Proteins Using Tol Mice Enables Development of Improved Tumor Models for Investigating Immunity and Metastasis. *Cancer Res* 2025; 85: 2165-2178.

22 Yi Z, Rong G, Guan Y, Li J, Chang L, Li H *et al*. Molecular landscape and efficacy of HER2-targeted therapy in patients with HER2-mutated metastatic breast cancer. *NPJ Breast Cancer* 2020; 6: 59.

23 Guardia GDA, Dos Anjos CH, Rangel-Pozzo A, Dos Santos FF, Birbrair A, Asprino PF *et al*. Alternative splicing generates HER2 isoform diversity underlying antibody-drug conjugate resistance in breast cancer. *Genome Res* 2025; 35: 1942-1958.

24 Rexer BN, Arteaga CL. Intrinsic and acquired resistance to HER2-targeted therapies in HER2 gene-amplified breast cancer: mechanisms and clinical implications. *Crit Rev Oncog* 2012; 17: 1-16.

William J. Muller, Ph.D.
Professor of Biochemistry
Rosalind and Morris Goodman Cancer Centre
McGill University
1160 Pine Avenue West
Montreal, QC, Canada H3A 1A3

April 4th, 2026

Dear Reviewers,

We sincerely thank the Reviewers for your insightful comments and feedback on our manuscript entitled ‘**HER2Δ16 directs luminal cell identity and estrogen receptor signaling in HER2+ breast cancer**’. Here, to address your concerns, we give a point-by-point clarification on each of the provided comments and outline how we have addressed all remaining concerns in our revised manuscript to strengthen the scientific merit of our study. Our comment addresses below are in red.

Comments from Reviewers:

Reviewer #1 (Remarks to the Author):

The manuscript has improved, and several concerns have been addressed. However, as noted in the author responses, additional experiments are required before the manuscript can be considered for publication. In particular, the following comments must be addressed:

1. Experiments proposed in response to Comment #7 need to be carried out.

The above comment refers to the request for functional assessment of anti-tumor immune cell responses in tamoxifen-treated $FE16^{flx/flx}$ tumors. We have now performed immunofluorescent staining for granzyme B expression within CD8+ cytotoxic T-cell populations from control or tamoxifen treated tumor cohorts and show a significant increase in T-cell activity within the tamoxifen treated group (New Supplementary Fig. 6A). Conversely, we performed immunofluorescent staining for the T-cell exhaustion marker PD1 and show a significant decrease in PD1+ CD3+ T-cells within the tamoxifen treated group (New Supplementary Fig. 6B). Together, this shows that not only is immune cell recruitment increased in tamoxifen treated tumors, but polarization towards an active anti-tumor immune response.

2. Response to Comment #8: Although the link between d16Her2 and ER activity (via MAPK) has been proposed, the mechanism driving the increased expression of ER in d16 tumors remains unclear. Since the d16-mediated luminal switch is a key finding, this issue must be addressed or acknowledged as a study limitation.

We agree with Reviewer #1 that the mechanisms utilized by HER2Δ16 to drive ER expression and shift tumors towards a luminal phenotype remains to be fully explored. We have amended our current discussion to both address this issue as a study limitation and provide further insight on how HER2Δ16 may be promoting a transcriptional program which confers luminal identity, offering epigenetic regulation through SUZ12 as one potential driver of this distinct HER2Δ16 transcriptional profile (included in New Supplementary Fig. 2B).

McGill

William J. Muller, Ph.D.

Professor of Biochemistry
Rosalind and Morris Goodman Cancer Centre
McGill University
1160 Pine avenue West
Montreal, QC, Canada H3A 1A3

Experiments proposed in response to Comment #10 need to be completed

The above comment refers to determining whether *ErbB4* expression is responsible for the increased ER activity and luminal phenotype across HER2Δ16 model systems. We have now profiled all HER2Δ16 transgenic model systems and human HER2+ cell lines for expression of *ErbB4* (New Supplementary Fig. 11). Although we confirm that ErbB4 expression is increased in Δ16IC tumors through bulk RNA-sequencing, we show no such correlations within EIC/FE16$^{flx/flx}$ or FE16$^{flx/flx}$ tumors (New Supplementary Fig. 11B). Within these models, ErbB4 remained undetectable at the RNA level through quantitative methods such as qRT-PCR and RNA ISH. Furthermore, no protein expression could be detected in any of these HER2Δ16 models (including Δ16IC tumors) through immunohistochemical staining suggesting ErbB4 is not largely implicated in the HER2Δ16 driven phenotype or increased ER-activity observed in these systems.

In human cell lines, we show that *ERBB4* transcript levels remain low and not significantly different between HER2Δ16 low (SK-BR-3) and HER2Δ16 high (ZR-75-30) cell lines (New Supplemental Fig. 11C). The JIMT-1 cell line was excluded from this analysis as it had no detectable *ERBB4* expression through qRT-PCR. The UACC812 cell line which expresses moderate levels of HER2Δ16 similarly showed no increase in *ERBB4* expression relative to SK-BR-3 cells. One exception is the SUM225CWN cell line, which has high HER2Δ16 expression, and does display elevated *ERBB4* transcript levels compared to the other cell lines utilized, suggesting within this individual case, *ERBB4* may contribute to the ER-phenotype observed.

Altogether, although ErbB4 levels can be increased in HER2Δ16 tumors, this phenomenon is not observed across the extensive catalog of transgenic models and human cell lines featured within this study, with ErbB4 transcript levels remaining low across model systems and unlikely to be the driving factor behind the ability of HER2Δ16 to confer a luminal identity.

Minor comments: The font size used for most figure annotations is very small. Improving figure readability would be beneficial.

We thank Reviewer #1 for bringing this to our attention. We have now amended all main and supplemental figures to increase annotation and axes sizes where possible. Additionally, we have changed the figures to show exact p-values for each statistical test performed.

Reviewer #2 (Remarks to the Author):

The authors have responded to each of the specific comments by the reviewers. The revised manuscript is significantly improved.

McGill

William J. Muller, Ph.D.

Professor of Biochemistry
Rosalind and Morris Goodman Cancer Centre
McGill University
1160 Pine avenue West
Montreal, QC, Canada H3A 1A3

We would again like to thank Reviewer #2 for taking the time to review our manuscript and for providing their feedback. We hope the additional revisions provided will only further improve the proposed manuscript.

Reviewer #3 (Remarks to the Author):

The rebuttal arguments and revised manuscript do not change my opinion regarding this work. In the rebuttal, the authors provide references that show d16HER2 detection in clinical samples and clinical correlations between d16HER2 and tumor aggressiveness, LN metastasis, and long-term outcomes. These have been repeatedly stated for decades now, and perhaps at initial discovery decades ago, correlative data formed a preliminary basis to suggest a hypothesis regarding the functional importance of d16HER2 in human cancer biology. But no such proof has emerged, and this hypothesis has failed the test of time, and only greatly artifactual and non-representative experimental scientific models have been produced that function to push a false agenda.

We would like to start by thanking Reviewer #3 for the time spent reviewing the revised work and articulating their viewpoint on HER2Δ16 in the field of cancer biology. We would like to again highlight our previous published research surrounding the clinical impact of HER2Δ16 as conducted in collaboration with the FINXX clinical trial. Within this patient cohort, survival analysis of HER2Δ16 positive and negative patients revealed that HER2Δ16 expression was associated with reduced recurrence-free survival (PMID: 37311021). Utilizing the same Δ16IC model as our current manuscript, we show that the transcriptional signature driven by Δ16IC expression is associated with worse overall survival in HER2-enriched patients stratified by PAM50 clustering (PMID: 37311021).

The clinical correlations can be due to a myriad of other factors such as HER2 levels, amplicon architecture, other tumor gene mutations, and many other factors. The rebuttal also cites evidence from other cancers including NSCLC and gastric cancers reporting d16HER2 expression, but they fail to mention that these are all also cases of HER2 amplification which constitutes the primary disease or resistance driver. These reports do nothing to strengthen the mechanistic hypothesis being pushed. The best summation of all the clinical and experimental evidence to date is that d16HER2 exists, but does not have biologically meaningful relevance as a disease driver or therapeutic target and the amplification and overexpression of HER2 is the principal disease driver. The current paper by Proud et al merely adds to the mass of inconclusive data in this realm. It should be placed in the public domain for the record, but I'm afraid there is nothing impactful to be gained by this work.

Within the same FINXX cohort outlined above, HER2Δ16 expression was noted in 14 patients otherwise classified as the triple-negative breast cancer subtype at the time of diagnosis which shows that HER2Δ16 alternative splicing events can occur independent of *ERBB2* amplification (PMID: 37311021). Additionally, within NSCLC cases (as mentioned above) *ERBB2*

amplification does occur in 43% (17/40) of tumors containing ERBB2 splice site alterations affecting exon 16 (PMID: 32727899). However, it still stands to reason that another 57% of these cases had no genetic amplification and importantly, few other mutations in known driving oncogenes (PMID: 32727899). Within the same dataset, 4 patients (3 ovarian cancer and 1 NSCLC) harbored only a splice site alteration in *ERBB2* and no other driver mutations (PMID: 32727899). Although *ERBB2* amplification is a critically relevant event in HER2+ tumorigenesis, alternative splicing of HER2Δ16 does not require amplification to occur.

We do not wish to further debate whether HER2Δ16 is a disease driver in HER2+ breast cancer in the absence of *ERBB2* amplification as this does not change the key hypothesis of the manuscript. Despite HER2+ breast cancer often coinciding with genetic amplification of the *ERBB2* locus, within the same paired cell lines, both murine NMuMG cells and human SK-BR-3 or UACC 812 cells show that increased HER2Δ16 expression either through genetic overexpression or naturally acquired through lapatinib-resistance results in upregulation of ESR1 and downstream ER targets (Fig. 2E-G, Fig. 6D-E, Supplementary Fig. 10B). This is independent of *ERBB2* amplification. Furthermore, expression of FE16$^{flx/flx}$ in combination with the wildtype (EIC) HER2 construct results in ER-positivity in the absence of any *ERBB2* amplification (Fig. 5, Supplementary Fig. 7B). Evaluating the scientific evidence provided by the manuscript, this shows that HER2Δ16 expression directly correlates with ER-positivity irrespective of amplification status.

Finally, as genetic amplification of *ERBB2* is a principal disease driver as summarized above in the response of Reviewer #3 this further highlights the value of the FE16$^{flx/flx}$ model as a tool to study human disease. Specifically, co-amplification of *ERBB2* amplicon members Stard3 and Grb7, as well as it's positive ER-status make the FE16$^{flx/flx}$ model a useful platform for studying HER2+/ER+ disease independent of the role of HER2Δ16, which we believe remains a major contribution to the field.

The rebuttal continues to argue for the case that this paper shows d16HER2 is a tumorigenic structural alteration, citing Fig 3 knockin model. But this again required the 50-fold gene amplification and massive overexpression of d16HER2 (Fig 3G), clearly demonstrating that the d16 structural event by itself was not sufficient to drive tumorigenesis. As is already very well established, overexpression is the transforming event of the HER2 oncogene and this knock-in experiment simply re-proved this already established hypothesis and disproved their contention that d16 deletion is a transforming alteration. The literature now has many mouse genetic models of this oncogene. It is very clear that the overexpression of the wildtype HER2/ERBB2 is tumorigenic, and overexpression of variant mutant forms is also tumorigenic, albeit with faster kinetics, however mutational alteration by itself is not tumorigenic (PMID 1359541, 15073130, 19364815, 21559085, 25164009). In the rebuttal the authors assert that the long latency and low penetrance of the genotype-phenotype should not disqualify this mutation as a tumor driver. But it is not the latency and penetrance alone that disqualify the driver status; rather it is the required HER2 gene amplification that develops and that is the unimpeachable disease driver that cannot

be ignored. In contrast to over-expression systems, the mutational knock-in of the endogenous ERBB2 (without overexpression) is not tumorigenic. The Muller group themselves have previously published such a model in 2000. They generated a knock-in mouse genetic model to introduce the activating point mutation in the transmembrane domain of ERBB2 and these mice developed tumors only late in life but importantly these were shown to have amplification of the mutant ERBB2 allele (PMID 10716706). The current d16HER2 work shows very similar results. In the previous work in 2000 they correctly concluded that the TM mutation is insufficient for tumorigenesis whereas in the current work they are attempting to spin the same negative result with the d16HER2 mutation work to suggest that the d16HER2 alteration is sufficient to drive tumorigenesis. I suggest the honest interpretation of the current work, which is that d16HER2 alteration is insufficient to drive tumorigenesis. I realize that the world of academia unfortunately does not reward negative results and that is a flaw of the system. But negative results are important, sometimes just as important as positive results. This work shows that d16HER2 is not tumorigenic by itself and the accompanying HER2 gene amplification is essential and sufficient for tumorigenesis. This is an important and revealing negative result. I suggest the honest conclusion of the work rather than the continued propagation of a false narrative.

Again, we agree that it is difficult to uncouple the effects of HER2Δ16 expression and amplification of the *ERBB2* locus as this is a frequent and critical event in HER2+ breast cancer. However as mentioned above, the manuscript provides several key experiments which allow us to assess the impact of HER2Δ16 on the luminal identity of cancer cells independent of amplification status. To find common ground, we have amended the manuscript to discuss the impact of *ERBB2* amplification further in detail while minimizing the role of HER2Δ16 in driving tumorigenesis. We also highlight the necessary advancements in the field, mainly the generation of blocking antibodies or antisense oligonucleotides specific for HER2Δ16, which would be necessary to fully define the contribution of HER2Δ16 in HER2+ tumor phenotypes irrespective of *ERBB2* amplification to experimentally answer the ongoing concerns of Reviewer #3. Although these experiments would be useful in uncoupling the impact of HER2Δ16 and *ERBB2* amplification we reiterate that this is unnecessary for the outcomes and major findings of the paper which instead focus on the role of HER2Δ16 in driving an ER-positive transcriptional program within established HER2+ tumors.

The rebuttal tries to defend their assertion in the paper the somatic TM mutations observed in the mouse models of Neu tumorigenesis are also seen in human cancers. The incidence of HER2 mutations is now a well studied fact. A multitude of studies have determined the percentage of HER2-amplified breast cancers that also harbor somatic HER2 mutations and show that it's rare, in the range of 1-2% and they are almost entirely within the hotspot regions of kinase domain and S310 (PMID 28164408)(PMID 27697991)(PMID 30301790)(and other studies). In their rebuttal, Proud et al highlight a really poor study of ctDNA (not tumor assays) reporting an exorbitantly high number of mutations spanning the entire HER2 gene, almost surely false, to support their claim that there are frequent mutations in the juxtamembrane region in HER2-amplified tumors as

Professor of Biochemistry
Rosalind and Morris Goodman Cancer Centre
McGill University
1160 Pine avenue West
Montreal, QC, Canada H3A 1A3

well (PMID 33145402). To rely on a false study to support the premise of your work does not engender credibility to the work. The scientific literature is so massive that you can do a google search and come up with virtually anything you are seeking. But a credible scientist should use judgement and context to select references.

The rebuttal tries unsuccessfully to rebut the myriad of papers in the literature with results conflicting with their conclusions regarding d16HER2 and ER signaling or drug resistance. There is no point in going back-and-forth with this line of debate. The field is fraught with artifactual findings, conflicting results, and ambiguity. The current manuscript simply piles on top of this body of ambiguity.

We agree that we cannot continue to debate the myriad of literature surrounding HER2Δ16 which offers conflicting results. Instead, we are only able to comment on the scientific merit of our submitted manuscript and those previously published utilizing these models which again reaffirm the phenotype that HER2Δ16 expression associates with an ER-transcriptional program and luminal cell identity. We continue to show this phenotype across multiple transgenic mouse models (Δ16IC, EIC/FE16$^{flx/flx}$, FE16$^{flx/flx}$) and several human cell lines (ZR-75-30, SUM225CWN, SK-BR-3 LR, UACC 812 LR) and have added further breast tumor microarray data to the manuscript to strengthen this claim (New Supplementary Fig. 9).

I'm afraid my conclusions about this work remain as they were in the first review. This paper continues along an exhausted line of nearly three decades of a failed narrative to promote a role for d16HER2 in human cancer biology and therapeutics. This work continues the pursuit of an agenda rather than the thread of science. I think the conclusions of this work regarding the biology of d16HER2 in human cancers and its implications for the treatment of human cancers are unjustified.

We would like to recognize that as no experimental revisions were proposed, we have focused on amending our discussion/emphasis of the study's findings and strengthening the empirical data surrounding HER2Δ16 expression in ER+ tumors. To that end, we have summarized the amended changes below.

1. Using breast tumor microarray samples from patients clinically scored as either HER2+/ER- or HER2+/ER+ we show that variable expression of HER2Δ16 was noted in about 55% of clinically scored HER2+/ER- patients, compared to 67% within HER2+/ER+ patients (New Supplementary Fig. 9). These statistics are similar to what was previously reported regarding HER2Δ16 expression within HER2+ tumors from the FINXX clinical trial (PMID: 37311021). This provides further evidence that HER2Δ16 is associated with ER positivity in clinical samples from HER2+ patients.

William J. Muller, Ph.D.

Professor of Biochemistry
Rosalind and Morris Goodman Cancer Centre
McGill University
1160 Pine avenue West
Montreal, QC, Canada H3A 1A3

2. We do acknowledge that though valuable tools in the study of human disease, transgenic systems are unable to fully mimic the genetic heterogeneity of HER2+ tumors. Due to the variation of HER2Δ16 expression within patients, it is difficult to determine the critical threshold of HER2Δ16 expression required to illicit intracellular or microenvironmental changes in HER2+ tumors. Furthermore, as it has been highlighted in the response of Reviewer #3, HER2+ breast cancer often coincides with amplification of the *ERBB2* locus, making it difficult to uncouple the effects of HER2Δ16 expression and other genetic amplification events. We have amended the manuscript to discuss this further in detail and to highlight the necessary advancements in the field, mainly the generation of blocking antibodies or antisense oligonucleotides specific for HER2Δ16, which would be necessary in order to fully define the contribution of HER2Δ16 in HER2+ tumor phenotypes irrespective of *ERBB2* amplification.

3. We recognize that the body of work surrounding the role of HER2Δ16 in driving tumorigenesis and therapeutic resistance remains conflicting. We are unable to harmonize the extensive body of work exploring the role of HER2Δ16 in solid malignancies conducted by other groups, and can only offer the empirical evidence provided in this manuscript which shows that in three independent transgenic models expressing HER2Δ16 (Δ16IC, EIC/FE16$^{flx/flx}$, FE16$^{flx/flx}$), two human cell lines expressing high HER2Δ16 levels (ZR-75-30, SUM225CWN) two paired parental and laptinib resistant cell lines exhibiting upregulation of HER2Δ16 (SK-BR-3, UACC 812) and human breast tumor microarray samples that HER2Δ16 expression significantly associates with an ER-positive transcriptional program and luminal cell identity relative to wildtype controls. As the molecular mechanisms promoted by HER2Δ16 to drive this program are not fully understood, we have made concessions to tone down the claimed role of HER2Δ16 in therapeutic resistance and highlight the dual role of HER2Δ16 connected to both luminal and EMT associated traits within primary tumors as further research is required.

We hope that the changes outlined will allow us to reach a mutually agreed resolution to the ongoing discussion surrounding the revised manuscript and avoid further debate on the field surrounding HER2Δ16 in cancer biology. We feel the scientific contributions of the manuscript, mainly the body of empirical evidence supporting the role of HER2Δ16 in luminal identity and ER-positivity, and the novelty of the FE16$^{flx/flx}$ model in permitting the study of ER+/HER2+ disease, valuable additions to the field. We again thank Reviewer #3 for the time spent reviewing the submitted manuscript and hope our concessions will aid in reaching a final resolution.

Reviewer #4 (Remarks to the Author): an early career researcher co-reviewer with Reviewer #2

I co-reviewed this manuscript with one of the reviewers who provided the listed reports. This is part of the Nature Communications initiative to facilitate training in peer review and to provide appropriate recognition for Early Career Researchers who co-review manuscripts.

McGill

William J. Muller, Ph.D.

Professor of Biochemistry
Rosalind and Morris Goodman Cancer Centre
McGill University
1160 Pine avenue West
Montreal, QC, Canada H3A 1A3

We would again like to thank Reviewer #4 for taking the time to review our manuscript and for providing their feedback.

Once again, we would like to sincerely thank our Reviewers for their insightful comments and constructive feedback. We are confident that this strengthened, revised manuscript successfully addresses your concerns to ensure that we provide a satisfactory research article for the readership of *Nature Communications*.

We thank you for your consideration and look forward to hearing from you soon.

Yours sincerely,

Professor William J. Muller, Ph.D
Canadian Research Chair in Molecular Oncology
Professor, Department of Biochemistry, McGill University
Rosalind and Morris Goodman Cancer Institute

McGill

William J. Muller, Ph.D.

Professor of Biochemistry
Rosalind and Morris Goodman Cancer Centre
McGill University
1160 Pine Avenue West
Montreal, QC, Canada H3A 1A3

May 17th, 2026

Dear Reviewers,

We once again thank you for your feedback on our manuscript entitled '**HER2Δ16 directs luminal cell identity and estrogen receptor signaling in HER2+ breast cancer**'. Here, to address any final concerns, we give a point-by-point clarification and outline how we have made the requested changes to strengthen the scientific merit of our study. Our comment addresses below are in red.

Comments from Reviewers:

Reviewer #1 (Remarks to the Author):

Overall concerns have been addressed. However, the new results involving SUZ12 appear too preliminary, as the current discussion does not fully align with established findings.

More specifically, SUZ12, a component of the PRC2 complex, is often linked to decreased ER expression. However, the discussion of SUZ12's potential role remains unclear (line 529-39). Although increased methylation of the SUZ12 promoter was observed in luminal B and embryonic stem cells (line 535-39), ChiP-X enrichment analysis indicates higher activity in D16 tumors. This ChiP-X finding appears to contradict the suggested methylation state. The paragraph can be clarified or the following sentences removed:

"This is evidenced by bulk RNA-sequencing of Δ16IC tumors which shows enrichment of SUZ12 in addition to ESR1 based on ChIP-X enrichment analysis (Supplementary Fig. 2B). Interestingly, higher levels of SUZ12 methylation have been reported within Luminal B tumors57. In embryonic stem cells, more differentiated CD24+ cell populations exhibited increased DNA methylation at promoters of SUZ12 target genes, suggesting that epigenetic regulation by SUZ12 is involved in maintaining luminal cell identity although this has yet to be studied in the context of HER2Δ16 expressing tumors"

We agree with the above comment and have subsequently removed the above sentences (previously Lines 533-539) from the final draft of the manuscript.

Reviewer #2 (Remarks to the Author):

The authors have responses to each of the additional specific points of the reviewers and included additional experiments to strengthen the manuscript. The revised manuscript is improved and the limitations are acknowledged.

We once again thank Reviewer #2 for their time in reviewing this manuscript.

McGill

William J. Muller, Ph.D.

Professor of Biochemistry
Rosalind and Morris Goodman Cancer Centre
McGill University
1160 Pine avenue West
Montreal, QC, Canada H3A 1A3

Reviewer #4 (Remarks to the Author):

I co-reviewed this manuscript with one of the reviewers who provided the listed reports. This is part of the Nature Communications initiative to facilitate training in peer review and to provide appropriate recognition for Early Career Researchers who co-review manuscripts.

We once again thank Reviewer #4 for their time in reviewing this manuscript.

Once again, we would like to sincerely thank our Reviewers for their insightful comments and constructive feedback throughout the revision process. We are confident that this final revised manuscript will provide a satisfactory research article for the readership of *Nature Communications*.

We thank you for your consideration and look forward to hearing from you soon.

Yours sincerely,

Professor William J. Muller, Ph.D
Canadian Research Chair in Molecular Oncology
Professor, Department of Biochemistry, McGill University
Rosalind and Morris Goodman Cancer Institute
